# Synthesis of Cyclic *N*-Acyl Amidines by [3 + 2] Cycloaddition of *N*-Silyl Enamines and Activated Acyl Azides

**DOI:** 10.3390/molecules27051696

**Published:** 2022-03-04

**Authors:** Dong Geun Jo, Changeun Kim, Sinjae Lee, Sooyeon Yun, Seewon Joung

**Affiliations:** Department of Chemistry, Mokpo National University, Muan 58554, Korea; whehdrms156@naver.com (D.G.J.); kcg3379@naver.com (C.K.); tlswo7318@naver.com (S.L.); yunsoo1402@naver.com (S.Y.)

**Keywords:** [3 + 2] cycloaddition, *N*-silyl enamine, B(C_6_F_5_)_3_, tetrahydroisoquinoline, acyl azide, spectroscopic analysis

## Abstract

In this study, we describe the synthesis of cyclic *N*-acyl amidines from readily available *N*-heteroarenes. The synthetic methodology utilized the versatile *N*-silyl enamine intermediates from the hydrosilylation of *N*-heteroarenes for the [3 + 2] cycloaddition reaction step. We evaluated various acyl azides and selected an electronically activated acyl azide, thereby achieving a reasonable yield of cyclic *N*-acyl amidines. We analyzed the relationship between the reactivity of each step and the electronic nature of substrates using in situ nuclear magnetic resonance spectroscopy. In addition, we demonstrated gram-scale synthesis using the proposed methodology.

## 1. Introduction

**Scheme 1:**
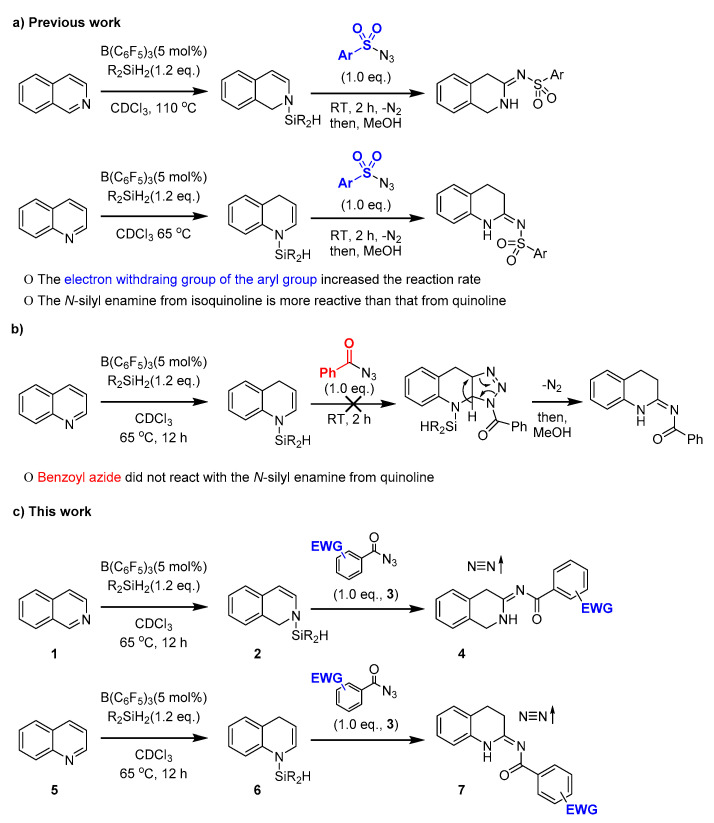
Reactivity of *N*-silyl enamines and organic azides. (**a**) Our previous work on the synthesis of cyclic (*Z*)-sulfonyl amidine via dearomative hydrosilylation and [3 + 2] cycloaddition cascade; (**b**) Limitation of the reactivity of acyl azide; (**c**) Cyclic (*Z*)-acyl amidine synthesis using activated acyl azides.

The development of synthetic pathways toward cyclic amidine derivatives is a research area of growing interest. Cyclic amidine derivatives have been shown to have interesting biological activities [1,2,3,4,5,6,7,8]; however, methods for their synthesis have several limitations [9,10,11]. Thus, several research groups have been developing synthetic methodologies for these derivatives [12,13]. Although most precedent pathways use cyclic amides as starting materials [11,12,13], our group recently reported a novel synthetic strategy that uses the readily available *N*-heteroarenes as starting materials and proceeds via a versatile *N*-silyl enamine intermediate (Scheme 1a) [14,15]. We achieved this intermediate through the B(C_6_F_5_)_3_ catalyzed dearomative hydrosilylation of *N*-heteroarene, which is considered an emerging area in organic synthesis [16,17,18,19]. In addition, although the reactivity of the first dearomative hydrosilylation has been widely reported [16,20], the reactivity of *N*-silyl enamine in the second [3 + 2] cycloaddition is relatively unexplored.

Versatile triazole intermediates are typically formed during the [3 + 2] cycloaddition reactions of enamine derivatives and organic azides. These intermediates have been utilized in various synthetic methodologies, such as amidine synthesis with a rearrangement involving nitrogen extrusion [21]. However, reports on the reactivity of *N*-silyl enamine are relatively rare, which is most likely due to the intrinsic instability of its N-Si bond at ambient conditions [22,23]. Nevertheless, the in situ use of *N*-silyl enamine is still an attractive strategy, in which the silyl group is used as the transient protecting group for the fragile but useful free enamine for organic synthesis [14,15].

Our previous works demonstrated [14,15] the wide substrate scope of *N*-heteroarenes; however, the scope of organic azides has been mainly limited to sulfonyl azides. Sulfonyl azides exhibit powerful reactivity; however, their reaction with acyl azides to synthesize the desired acyl amidine product has not yet been realized (Scheme 1b) [14]. Interestingly, we recently found that the electron-withdrawing groups of sulfonyl azides increased the reactivity of the cycloaddition reaction [15]. This prompted us to develop a [3 + 2] cycloaddition reaction that uses the electron-withdrawing group on the less reactive acyl azides (Scheme 1c). Herein, we report the synthesis of cyclic acyl amidines from *N*-heteroarenes via dearomative hydrosilylation and the unique [3 + 2] cycloaddition of the resulting *N*-silyl enamine intermediate and the electronically activated acyl azides.

## 2. Results and Discussion

**Scheme 2:**
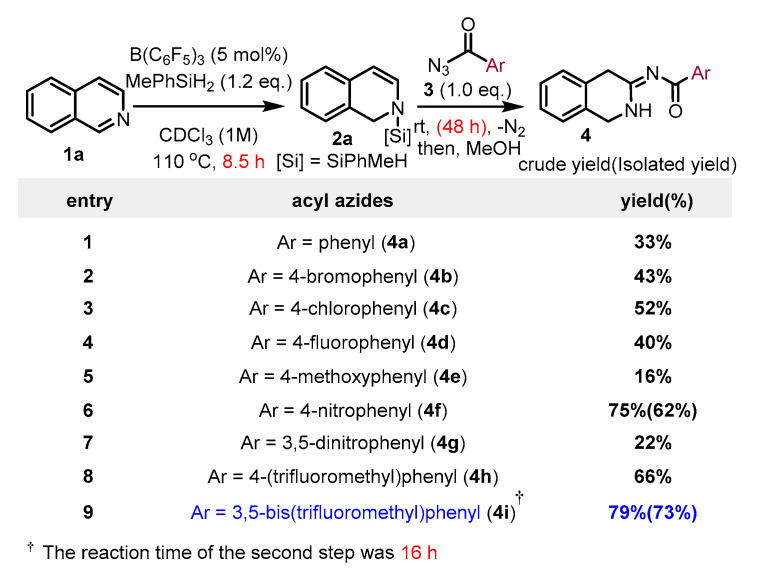
The reactivity of acyl azides **3** toward *N*-silyl enamine **2a** from isoquinoline **1a**.

The reactivity of *N*-silyl enamine from isoquinoline **1a** was found to be promising; thus, we began our study with isoquinoline [15]. Using the previously optimized conditions for the dearomative mono-hydrosilylation of **1a**, we obtained the *N*-silyl enamine intermediate with good yield in a nuclear magnetic resonance (NMR) cell. The results from the following addition of acyl azides with different substituents are listed in Scheme 2. In contrast to the reaction of *N*-silyl enamine from quinoline [14], the *N*-silyl enamine **2a** from **1a** reacted with benzoyl azide **3a** to produce the acyl amidine product **4a** with a reasonable crude yield in 48 h. Acyl azides with halogen substituents (**3b**–**3d**) also worked well with *N*-silyl enamine **2a** to achieve cyclic acyl amidines **4b**–**4d**. However, the electron-rich azide **3e** was less reactive than the electron-poor azides **3b**–**3d**, which is consistent with our previous reports on sulfonyl azides [14,15]. We therefore examined more electron-poor azides (**3f**–**3i**) with nitro or trifluoromethyl substituents to improve the reactivity of acyl azides (**4f**–**4i**). Indeed, the strong electron-withdrawing substituents improved the reactivity of acyl azides. However, the acyl amidine products **4f**–**4h** were relatively unstable due to hydrolysis during the work-up and purification process. Therefore, the 3,5-bis(trifluoromethyl)benzoyl azide **3i** was considered to be the appropriate acyl azide to achieve the stable amidine product **4i**. Notably, the conversion of **3i** to **4i** was achieved within a short timeframe of 16 h. We also confirmed the (*Z*) configuration of *N*-acyl amidine using an X-ray diffraction analysis of a single crystal of **4i** (CCDC 2142037).

**Scheme 3:**
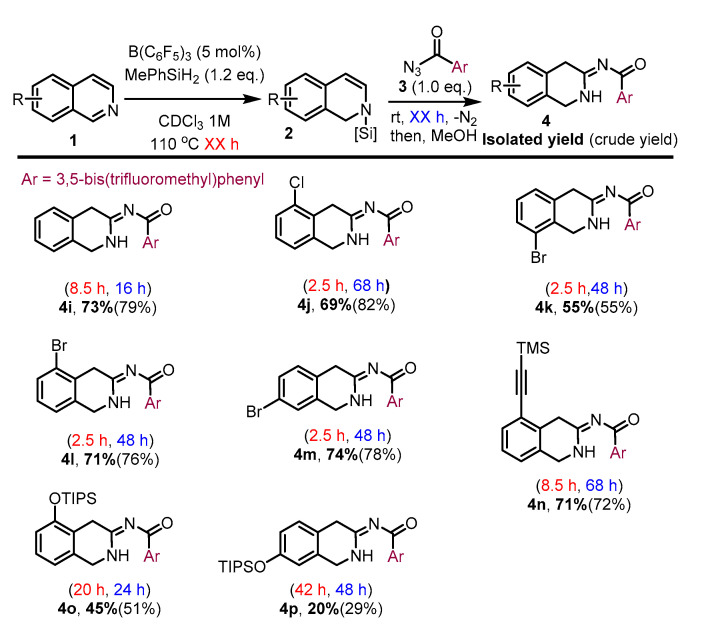
Substrate scope of isoquinolines **1** for the synthesis of acyl amidines **4**.

Using the optimized azide **3i**, we explored the substrate scope of the isoquinolines **1** (Scheme 3). The 5-chloroisoquinoline **1j** reacted with **3i** to form the cyclic amidine **4j**. Interestingly, the reactivity in each step of the cascade pathway was distinct from that of the reaction of **1a**. The conversion in the first dearomatization step was completed within 2.5 h because the electron-poor quinoline derivative was more reactive toward the Lewis acid-catalyzed hydrosilylation. However, the second cycloaddition step was much slower with the electron-poor *N*-silyl enamine **2j** than **2a**. This suggested that the *N*-silyl enamine **2** acted as a nucleophile in the second step. Notably, the acyl amidine **4j** was obtained with good yield due to the cascade synthetic approach. Meanwhile, the reaction of the isoquinolines with a bromo substituent at different positions (**1k**–**1m**) proceeded smoothly, producing acyl amidines **4k**–**4m** in 48 h. Isoquinoline **1n** with an alkyne substituent at the 5-position reacted well with **3i** to afford **4n** in a good yield. The reactions of isoquinolines **1o**–**1p** with an electron-donating substituent also produced cyclic acyl amidines **4o**–**4p**; however, the yields were moderate due to the low reactivity of **1o**–**1p** toward the first hydrosilylation step. The second [3 + 2] cycloaddition step was quite fast with an electron rich substrate.

**Scheme 4:**
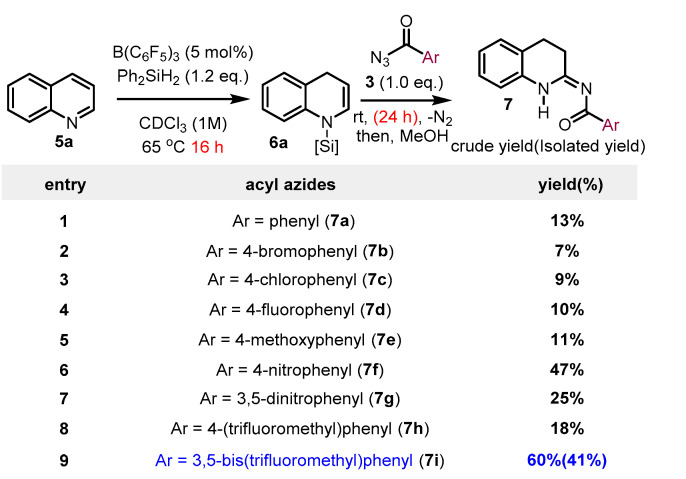
The reactivity of acyl azides **3** toward *N*-silyl enamine **6a** from quinoline **5a**.

Next, we surveyed the reactivity of various acyl azides **3** for the *N*-silyl enamine **6a** from quinoline **5a** (Scheme 4). Although the reactivity of **6a** was not sufficient for benzoyl azide **3a** in 2 h [14], the cyclic acyl amidine **7a** was obtained within 24 h; however, the yield was low, at 13%. Meanwhile, from the screening of acyl azides **3b**–**3i** with different electronic natures, acyl azides with 4-nitro (**3f**) and 3,5-bis(trifluoromethyl) (**3i**) were effective toward **6a**, leading to a moderate to good yield of the cyclic amidines **7f** and **7i**, respectively. Acyl azides with a strong electron-withdrawing 3,5-dinitro substituent (**3g**) were converted efficiently to N-silyl enamine 6a; however, the resulting acyl amidine **7g** was unstable under ambient conditions. Relatively poor electron-withdrawing (**3b**–**3d** and **3h**) and electron-donating **3e** acyl azides were unable to convert **6a** to cyclic amidines **7** with reasonable yields. Therefore, the acyl azide **3i** was considered the most appropriate acyl azide for the synthesis of **7** from **5a** via Scheme 4. In addition, the *N*-silyl enamine **6a**, which was unreactive toward benzoyl azide **3a** can now be utilized for the synthesis of acyl amidines **7**.

**Scheme 5:**
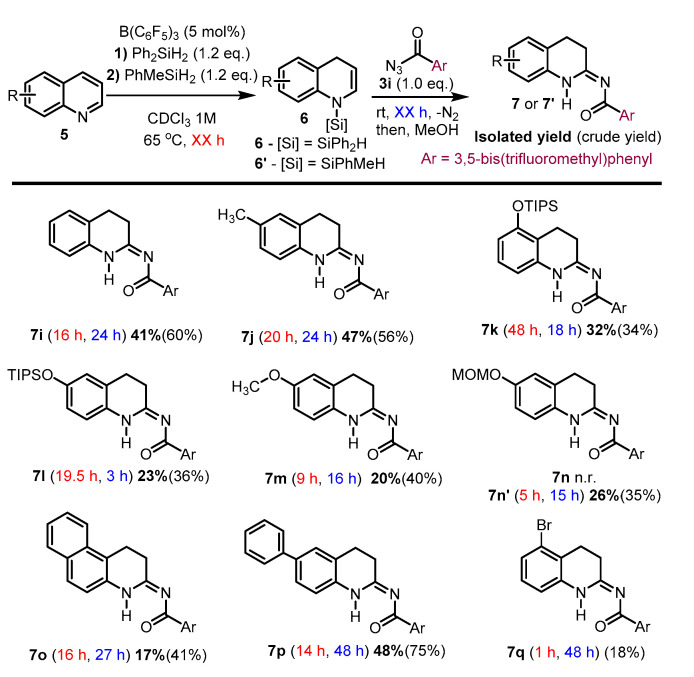
Substrate scope of quinolines **5** for the acyl amidine synthesis of **7** or **7′**.

We investigated the substrate scope of quinolines **5** using the optimized acyl azide **3i** (Scheme 5). First, an *N*-silyl enamine **6** from **5** was produced with diphenylsilane (Ph_2_SiH_2_) [14]. The reactions of the electron rich *N*-silyl enamines (**6j**–**6p**) and electron-poor azide **3i** resulted in cyclic acyl amidines (**7j**–**7p**) with low to moderate yields. The *N*-silyl enamines with electron-donating substituents (**6j**–**6p**) were sufficiently reactive in the [3 + 2] cycloaddition step; however, the conversions in the first hydrosilylation step were relatively slow. Especially, the 6-methoxymethyloxyquinoline **5n** was not converted to **6n** with Ph_2_SiH_2_. Therefore, we decided to use the more reactive methylphenylsilane (MePhSiH_2_) for conversion in the first hydrosilylation of **5n** to achieve *N*-silyl enamine 6n’ with reasonable yield [15]. An isolable amount of acyl amidine **7n′** was subsequently obtained. We note that the acyl azide **3i** was, however, ineffective toward other *N*-silyl enamines with an electron-withdrawing group. For example, the cycloaddition of **3i** and bromo substituted *N*-silyl enamine **6q** was not accomplished due to the low reactivity of **6q** toward the cycloaddition step.

**Scheme 6:**
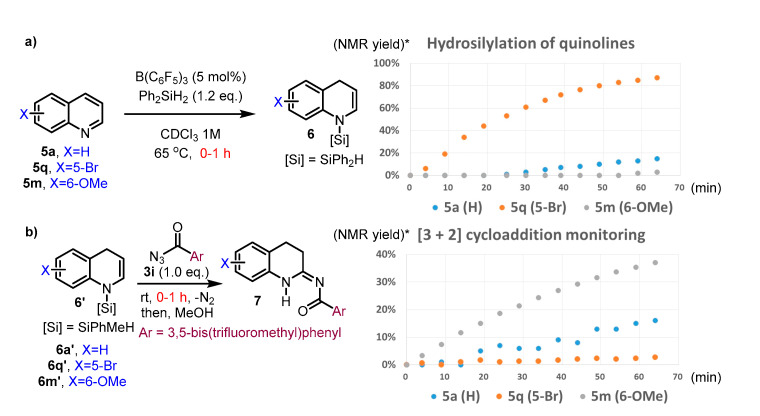
Relative rates of hydrosilylation and [3 + 2] cycloaddition: (**a**) Relationship between the relative rate of first de-aromative hydrosilylation and substituents on quinoline; (**b**) Relationship between the relative rate of [3 + 2] cycloaddition and substituents on quinoline. * NMR yields were determined by using 1,2-tetrachloroethane as an internal standard.

The reaction rate in each step was found to be relatively slow; thus, the electronic effect of the quinolines on the reaction rate could be observed through in situ NMR monitoring. (Supplementary Materials). We compared the initial reaction progression through ^1^H NMR using quinolines containing different substituents (Scheme 6). The borane catalyzed hydrosilylation step of the quinolines (**5a**, **5m**, and **5q**) was first investigated with Ph_2_SiH_2_. The initial reaction rate of 5-bromoquinoline (**5q**) was faster than 6-methoxyquinoline (**5m**) as expected. This indicated that the rate-determining step in the hydrosilylation was the dearomative borohydride addition [24]. Next, we examined the [3 + 2] cycloaddition step with *N*-silyl enamines **6′**. All of the proposed *N*-silyl enamines (**6a′**, **6m′**, and **6q′**) were generated with MePhSiH_2_ via the proper conversion of **5a**, **5m**, and **5q**. During the NMR reaction monitoring, we observed a faster cycloaddition of the electron-rich *N*-silyl enamine **6m′** than the electron-poor *N*-silyl enamine **6q′**. This result verified the correlation between the electron density of *N*-silyl enamine **6′** and the reaction rate of the cycloaddition step.

**Scheme 7:**
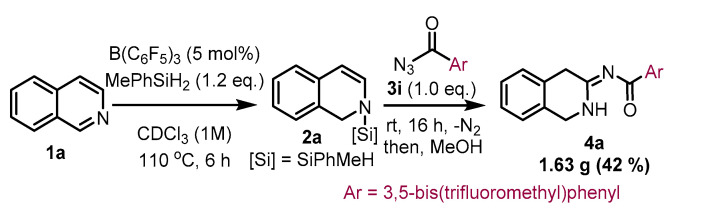
Gram-scale reaction of the proposed cyclic acyl amidine synthesis.

The synthetic applicability of the presented cyclic acyl amidine synthesis was then explored (Scheme 7). A 10 mmol initial scale reaction of **1a** smoothly produced 1.63 g (42% yield) of cyclic amidine **4a**. This result demonstrated the scalability of our methodology for the potential preparation of practical amounts of useful cyclic acyl amidines.

## 3. Conclusions

We successfully prepared cyclic acyl amidines from versatile *N*-silyl enamines and acyl azides. An electronically activated acyl azide (**3i**) was found to be the most optimal acyl azide for the [3 + 2] cycloaddition step. The substrate scope from the *N*-silyl enamine from isoquinolines to quinolines is wide. The progression of the initial reaction was monitored using NMR, which clearly demonstrated the relationship between the electron density of *N*-silyl enamine and reactivity in each synthetic step. Finally, the synthetic utility of the proposed methodology was demonstrated via the gram-scale reaction.

## 4. Experimental Section

### 4.1. General Considerations

Unless otherwise stated, all catalytic reactions were carried out under an argon atmosphere. Chloroform-*d* was purchased from Cambridge Isotope Laboratories, Inc. (Tewksbury, MA, USA), degassed and used as a solvent without additional purification for optimization, as a substrate scope. Tris(pentafluorophenyl)borane was purchased from TCI korea (Seoul, Korea) and Acros (ThermoFisher Korea, Seoul, Korea), and was stored at −15 °C. All other reagents were directly used as purchased without further purification unless otherwise stated.

Analytical thin-layer chromatography (TLC) was performed on pre-coated silica gel 60 F254 plates (Intertechnologies, Seoul, Korea). Visualization on TLC was achieved by the use of UV light (254 nm, Collégien, France), exposure to treatment with acidic *p*-anisaldehyde, phosphomolybdic acid and potassium permanganate stain followed by heating. Column chromatography was undertaken on silica gel (400–630 mesh) using a proper eluent. ^1^H NMR (Jeol, Tokyo, Japan) was recorded using Jeol ECZ-500R (500 MHz) for the characterization of compounds. Chemical shifts were quoted in parts per million (ppm), referenced to tetramethylsilane: 0.00 ppm (singlet). Furthermore, ^13^C{^1^H} NMR (Jeol, Tokyo, Japan) was recorded on Jeol ECZ-500R (125 MHz) and was fully decoupled by broad-band proton decoupling. Chemical shifts were reported in ppm referenced to the center of a triplet at 77.0 ppm of CDCl_3_. Infrared (IR, PerkinElmer Korea, Seoul, Korea) spectra were recorded using a Perkin Elmer Frontier ATR-FT-IR spectrometer, ν_max_ in cm^−1^. High resolution mass spectra (Jeol, Tokyo, Japan) were obtained by using EI and FAB method from Korea Basic Science Institute (Daegu). X-ray diffraction (Bruker, Billerica, MA, USA) data were collected using a Bruker D8 QUEST coated with Parabar oil under a stream of N_2_ (g) at 173 K.

### 4.2. Substrate Scope of the Isoquinoline for the Synthesis of Acyl Amidine

Step 1: To a B(C_6_F_5_)_3_ catalyst (0.025 mmol, 5 mol%) in an NMR tube CDCl_3_ (0.5 mL) and silanes (0.6 mmol, 1.2 equiv.) were added at room temperature, H_2_ bubbles were observed and TCE (0.3 mmol) or mesitylene was added as internal standard. Isoquinolines (**1a, 1i**–**1p**) (0.5 mmol, 1.0 equiv.) was subsequently added to the above solution and quickly shaken once before heating up to 110 °C in an oil bath for the indicated reaction time. The mixture was subjected to an NMR to verify the conversion and yields of reactions.

Step 2: In the crude reaction mixture from the first step, acyl azide **3i** (0.5 mmol, 1.0 equiv.) was added at room temperature and the NMR was indicated in the reaction time. The resulting mixture was quenched by MeOH addition, silica filter, and DCM wash. The resulting crude mixture was purified by column chromatography.

(*Z*)-*N*-(1,4-Dihydroisoquinolin-3(2*H*)-ylidene)-3,5-bis(trifluoromethyl)benzamide (Scheme 3, **4i**): Compound **4i** was prepared from **1a** and **3i** according to the above general procedure with 8.5 h for step 1 and 16 h for step 2; eluent: ethyl acetate:hexane = 2:8; yield: 193.1 mg (73%); yellowish solid; ^1^H NMR (500 MHz, CDCl_3_) δ 11.92 (s, 1H), 8.64 (s, 2H), 7.88 (s, 1H), 7.27–7.18 (m, 3H), 7.17–7.13 (m, 1H), 4.56 (t, *J* = 2.2 Hz, 2H), 3.78 (t, *J* = 2.3 Hz, 2H); ^13^C NMR (125 MHz, CDCl_3_) δ 176.5, 171.1, 139.7, 131.4 (q, 2C, *J* = 33.6 Hz), 130.6, 130.2, 129.5 (2C), 128.1, 127.8, 127.2, 125.5, 124.9 (p, *J* = 3.5 Hz), 123.4 (q, 2C, *J* = 272.8 Hz), 45.3, 36.6; IR (cm^−1^) 1738, 1607, 1488, 1312, 1281, 1119, 911, 742, 682; HRMS (EI): Calculated for C_18_H_12_F_6_N_2_O [M]^+^: 386.0854, Found: 386.0851.

(*Z*)-*N*-(5-Chloro-1,4-dihydroisoquinolin-3(2*H*)-ylidene)-3,5-bis(trifluoromethyl)benzamide (Scheme 3, **4j**): Compound **4j** was prepared from **1j** and **3i** according to the above general procedure with 2.5 h for step 1 and 68 h for step 2; eluent: ethyl acetate:hexane = 2:8; yield: 156 mg (69%); White yellow solid; ^1^H NMR (500 MHz, CDCl_3_) δ 12.12 (s, 1H), 8.73 (d, *J* = 1.8 Hz, 2H), 7.98 (s, 1H), 7.38 (dd, *J* = 8.0, 1.1 Hz, 1H), 7.24 (d, *J* = 7.8 Hz, 1H), 7.14 (d, *J* = 7.6 Hz, 1H), 4.69 (t, *J* = 2.4 Hz, 2H), 3.94 (t, *J* = 2.4 Hz, 2H); ^13^C NMR (125 MHz, CDCl_3_) δ 176.6, 169.9, 139.6, 133.4, 131.5, 131.4 (q, 2C, *J* = 33.4 Hz), 129.5 (d, 2C, *J* = 3.7 Hz), 128.6, 128.4, 128.2, 124.9 (p, *J* = 3.5 Hz), 123.8, 123.3 (q, 2C, *J* = 272.8 Hz), 45.2, 33.5; IR (cm^−1^) 1614, 1578, 1287, 1111, 911, 777, 701, 681; HRMS (EI): Calculated for C_18_H_11_ClF_6_N_2_O [M]^+^: 420.0464, Found: 420.0460. 

(*Z*)-*N*-(8-Bromo-1,4-dihydroisoquinolin-3(2*H*)-ylidene)-3,5-bis(trifluoromethyl)benzamide (Scheme 3, **4k**):Compound **4k** was prepared from **1k** and **3i** according to the above general procedure with 2.5 h for step 1 and 48 h for step 2; eluent: ethyl acetate:hexane = 1:9; yield: 107.0 mg (55%); White yellow solid; ^1^H NMR (500 MHz, CDCl_3_) δ 12.03 (s, 1H), 8.72 (s, 2H), 7.98 (s, 1H), 7.53 (dd, *J* = 7.6, 1.5 Hz, 1H), 7.26–7.19 (m, 2H), 4.73 (t, *J* = 2.3 Hz, 2H), 3.90 (t, *J* = 2.3 Hz, 2H); ^13^C NMR (125 MHz, CDCl_3_) δ 176.6, 170.1, 139.5, 132.6, 131.4 (q, 2C, *J* = 33.4 Hz), 131.2, 129.8, 129.5 (q, 2C, *J* = 2.75 Hz), 129.4, 126.9, 125.2–124.8 (m), 123.3 (q, 2C, *J* = 272.7 Hz), 121.4, 45.8, 36.2; IR (cm^−1^) 1603, 1329, 1283, 1244, 1121, 774, 682; HRMS (EI): Calculated for C_18_H_11_BrF_6_N_2_O [M]^+^: 463.9959, Found: 463.9956.

(*Z*)-*N*-(5-Bromo-1,4-dihydroisoquinolin-3(2*H*)-ylidene)-3,5-bis(trifluoromethyl)benzamide (Scheme 3, **4l**): Compound **4l** was prepared from **1l** and **3i** according to the above general procedure with 2.5 h for step 1 and 48 h for step 2; eluent: ethyl acetate:hexane = 2:8; yield: 164.8 mg (70.9%); yellowish solid; ^1^H NMR (500 MHz, CDCl_3_) δ 12.13 (s, 1H), 8.74 (s, 2H), 7.99 (s, 1H), 7.60 (dd, J = 5.5, 3.7 Hz, 1H), 7.20 (s, 1H), 7.19 (d, *J* = 1.9 Hz, 1H), 4.71 (s, 2H), 3.95 (t, *J* = 2.4 Hz, 2H); ^13^C NMR (125 MHz, CDCl_3_) δ 176.7, 170.2, 139.6, 132.0, 131.7, 131.5 (q, 2C, *J* = 33.4 Hz), 130.2, 129.6 (d, 2C, *J* = 4.0 Hz), 128.6, 125.1–124.8 (m), 124.6, 124.4 (q, 2C, *J* = 274 Hz), 123.8, 45.4, 36.4; IR (cm^−1^) 1614, 1487, 1325, 1285, 1164, 1119, 1109, 911, 885, 682; HRMS (EI): Calculated for C_18_H_11_BrF_6_N_2_O [M]^+^: 463.9959, Found: 463.9961.

(*Z*)-*N*-(7-Bromo-1,4-dihydroisoquinolin-3(2*H*)-ylidene)-3,5-bis(trifluoromethyl)benzamide (Scheme 3, **4m**): Compound **4m** was prepared from **1m** and **3i** according to the above general procedure with 2.5 h for step 1 and 48 h for step 2; eluent: DCM:hexane = 8:2; yield: 171.6 mg (74%); White yellow solid; ^1^H NMR (500 MHz, CDCl_3_) δ 12.00 (s, 1H), 8.72 (s, 2H), 7.98 (s, 1H), 7.47 (dd, *J* = 8.1, 2.0 Hz, 1H), 7.42 (d, *J* = 1.9 Hz, 1H), 7.18 (d, *J* = 8.1 Hz, 1H), 4.62 (s, 2H), 3.81 (s, 2H); ^13^C NMR (125 MHz, CDCl_3_) δ 176.6, 170.4, 139.5, 132.3, 131.4 (q, 2C, *J* = 33.6 Hz), 131.2, 129.6, 129.5 (q, 2C, *J* = 2.6 Hz), 129.4, 128.5, 125.1–124.8 (m, *J* = 3.7 Hz), 124.4 (q, 2C, *J* = 270.5 Hz), 120.9, 44.8, 36.1; IR (cm^−1^)1613, 1584, 1334, 1268, 1122, 841, 699, 680; HRMS (EI): Calculated for C_18_H_11_BrF_6_N_2_O [M]^+^: 463.9959, Found: 463.9956.

(*Z*)-3,5-Bis(trifluoromethyl)-*N*-(5-((trimethylsilyl)ethynyl)-1,4-dihydroisoquinolin-3(2*H*)-ylidene)benzamide (Scheme 3, **4n**): Compound **4n** was prepared from **1n** and **3i** according to the above general procedure in 8.5 h for step 1 and 68 h for step 2; eluent: ethyl acetate:hexane = 15:85; yield: 170.7 mg (71%); Yellow solid; ^1^H NMR (500 MHz, CDCl_3_) δ 12.06 (s, 1H), 8.75 (s, 2H), 7.98 (s, 1H), 7.48 (dd, *J* = 7.7, 1.4 Hz, 1H), 7.24 (d, J = 7.7 Hz, 1H), 7.18 (d, *J* = 7.6, 1.3 Hz, 1H), 4.65 (s, 2H), 4.01 (s, 2H), 0.34 (s, 9H); ^13^C NMR (125 MHz, CDCl_3_) δ 176.5, 170.6, 139.6, 132.4, 131.7, 131.4 (q, 2, *J* = 33.4 Hz), 130.1, 129.5 (q, 2C, *J* = 3.8 Hz), 126.9, 125.5, 124.9 (p, *J* = 3.6 Hz), 123.3 (q, 2C, *J* = 272.8 Hz), 122.4, 101.7, 101.1, 45.2, 34.8, −0.1 (s, 3C); IR (cm^−1^) 1610, 1310, 1278, 1174, 1111, 842, 761, 696, 680; HRMS (EI): Calculated for C_23_H_20_F_6_N_2_OSi [M]^+^: 482.1249, Found: 482.1253.

(*Z*)-3,5-Bis(trifluoromethyl)-*N*-(5-((triisopropylsilyl)oxy)-1,4-dihydroisoquinolin-3(2*H*)-ylidene)benzamide (Scheme 3, **4o**): Compound **4o** was prepared from **1o** and **3i** according to the above general procedure with 20 h for step 1 and 24 h for step 2; eluent: ethyl acetate:hexane = 1:3; yield: 122.5 mg (45%); Yellow solid; ^1^H NMR (500 MHz, CDCl_3_) δ 12.05 (s, 1H), 8.73 (s, 2H), 7.97 (s, 1H), 7.15 (t, *J* = 7.9 Hz, 1H), 6.82 (d, 1H), 6.80 (d, 1H), 4.65 (t, *J* = 2.5 Hz, 2H), 3.83 (t, *J* = 2.5 Hz, 2H), 1.44–1.32 (m, 3H), 1.16 (d, *J* = 7.5 Hz, 18H); ^13^C NMR (125 MHz, CDCl_3_) δ 176.5, 171.1, 153.3, 139.9, 131.3 (q, 2C, *J* = 33.7 Hz), 131.2, 129.5 (s, 2C), 127.7, 124.8, 123.4 (q, 2C, *J* = 272.6 Hz), 120.9, 117.6, 116.9, 45.2, 30.9, 18.0 (s, 6C), 13.0 (s,3C); IR (cm^−1^) 1615, 1587, 1463, 1311, 1273, 1127, 881, 771, 680; HRMS (EI): Calculated for C_27_H_32_F_6_N_2_O_2_Si [M]^+^: 558.2137, Found: 558.2139.

(*Z*)-3,5-Bis(trifluoromethyl)-*N*-(7-((triisopropylsilyl)oxy)-1,4-dihydroisoquinolin-3(2*H*)-ylidene)benzamide (Scheme 3, **4p**): Compound **4p** was prepared from **1p** and **3i** according to the above general procedure with 42 h for step 1 and 48 h for step 2; eluent: acetone:hexane = 1:3; yield: 55.8 mg (20%); Yellow solid; ^1^H NMR (500 MHz, CDCl_3_) δ 12.01 (s, 1H), 8.72 (s, 2H), 7.97 (s, 1H), 7.13 (d, *J* = 8.3 Hz, 1H), 6.85 (dd, J = 8.3, 2.5 Hz, 1H), 6.76 (d, *J* = 2.4 Hz, 1H), 4.58 (s, 2H), 3.79 (s, 2H), 1.31–1.21 (m, 3H), 1.11 (d, *J* = 7.4 Hz, 18H); ^13^C NMR (125 MHz, CDCl_3_) δ 176.5, 171.5, 155.3, 139.8, 131.4 (q, 2C, *J* = 33.5 Hz), 131.2, 129.5 (d, 2C, *J* = 4.2 Hz), 128.7, 125.0–124.6 (m), 123.4 (q, 2C, *J* = 272.8 Hz), 122.6, 119.7, 116.6, 45.3, 35.8, 17.9 (s, 6C), 12.6 (s, 3C); IR (cm^−1^) 1607, 1274, 1130, 971, 881, 821, 680; HRMS (EI): Calculated for C_27_H_32_F_6_N_2_O_2_Si [M]^+^: 558.2137, Found: 558.2134. 

### 4.3. Substrate Scope of the Quinoline for the Acyl Amidine Synthesis

Step 1: To a B(C_6_F_5_)_3_ catalyst (0.025 mmol, 5 mol%) in an NMR tube CDCl_3_ (0.5 mL) and silanes (0.6 mmol, 1.2 equiv.) were added at room temperature, H_2_ bubbles were observed and TCE (0.3 mmol) or mesitylene was added as internal standard. Quinoline (**5i**–**5q**) (0.5 mmol, 1.0 equiv.) was subsequently added to the above solution and quickly shaken once before heating to 65 °C in an oil bath for the indicated reaction time. The mixture was subjected to NMR to verify the conversion and yields of reactions.

Step 2: In the crude reaction mixture from the first step acyl azide **3i** (0.5 mmol, 1.0 equiv.) was added at room temperature in NMR for the indicated reaction time. The resulting mixture was quenched by MeOH addition, silica filter, and DCM wash. The resulting crude mixture was purified by column chromatography. 

(*Z*)-*N*-(3,4-Dihydroquinolin-2(1*H*)-ylidene)-3,5-bis(trifluoromethyl)benzamide (Scheme 5, **7i**): Compound **7i** was prepared from **5i** and **3i** according to the above general procedure with 16 h for step 1 and 24 h for step 2; eluent: ethyl acetate:hexane = 5:95; yield: 799.0 mg (41%); White solid; ^1^H NMR (500 MHz, CDCl_3_) *δ* 13.03 (s, 1H), 8.75 (s, 2H), 8.00 (s, 1H), 7.27 (td, *J* = 7.4, 1.2 Hz, 1H), 7.22 (d, *J* = 7.5 Hz, 1H), 7.14 (td, *J* = 7.4, 1.2 Hz, 1H), 6.98 (d, *J* = 7.8 Hz, 1H), 3.06–2.99 (m, 2H), 2.95–2.89 (m, 2H); ^13^C NMR (125 MHz, CDCl_3_) *δ* 176.8, 168.4, 139.3, 134.7, 131.5 (q, *J* = 33.5 Hz, 2C), 129.7 (q, *J* = 3.8 Hz, 2C), 128.4, 127.9, 125.6, 125.5, 125.2 (p, *J* = 3.8 Hz), 123.3 (q, *J* = 272.7 Hz, 2C), 117.6, 30.3, 23.9; IR (cm^−1^) 1573, 1349, 1281, 1268, 1254, 1119, 908,756, 700, 683; HRMS (EI): Calculated for C_18_H_12_F_6_N_2_O [M]^+^: 386.0854, Found: 386.0852.

(*Z*)-*N*-(6-Methyl-3,4-dihydroquinolin-2(1*H*)-ylidene)-3,5-bis(trifluoromethyl)benzamide (Scheme 5, **7j**): Compound **7j** was prepared from **5j** and **3i** according to the above general procedure with 20 h for step 1 and 24 h for step 2; eluent: ethyl acetate:hexane = 5:95; yield: 93.1 mg (47%); Yellow solid; ^1^H NMR (500 MHz, CDCl_3_) *δ* 13.03 (s, 1H), 8.73 (s, 2H), 7.98 (s, 1H), 7.03 (dd, *J* = 8.0, 1.9 Hz, 1H), 7.00 (s, 1H), 6.85 (d, *J* = 7.9 Hz, 1H), 3.00 –2.93 (m, 2H), 2.91–2.86 (m, 2H), 2.32 (s, 3H); ^13^C NMR (125 MHz, CDCl_3_) *δ* 176.7, 168.1, 139.4, 135.5, 132.2, 131.5 (q, *J* = 33.5 Hz, 2C), 129.6 (q, *J* = 3.8 Hz, 2C), 129.1, 128.3, 125.3, 125.0 (p, *J* = 3.8 Hz), 123.3 (q, *J* = 272.8 Hz, 2C), 117.4, 30.4, 23.9, 20.9.; IR (cm^−1^) 1605, 1566, 1343, 1268, 1233,1252, 1163, 1120, 909, 808, 682; HRMS (EI): Calculated for C_19_H_14_F_6_N_2_O [M]^+^: 400.1010, Found: 400.1012.

(*Z*)-3,5-Bis(trifluoromethyl)-*N*-(5-((triisopropylsilyl)oxy)-3,4-dihydroquinolin-2(1*H*)-ylidene)benzamide (Scheme 5, **7k**): Compound **7k** was prepared from **5k** and **3i** according to the above general procedure with 48 h for step 1 and 18 h for step 2; eluent: ethyl acetate:hexane = 5:95; yield: 89.2 mg (32%); Yellow liquid; ^1^H NMR (500 MHz, CDCl_3_) *δ* 12.98 (s, 1H), 8.76 (s, 2H), 8.00 (s, 1H), 7.09 (t, *J* = 8.0 Hz, 1H), 6.67 (dd, *J* = 8.3, 1.0 Hz, 1H), 6.59 (d, *J* = 2 Hz, 1H), 3.01 (dd, *J* = 8.8, 6.8 Hz, 2H), 2.87 (dd, *J* = 2, 2.2 Hz 2H), 1.39–1.23 (m, 3H), 1.13 (d, *J* = 7.5 Hz, 18H).; ^13^C NMR (125 MHz, CDCl_3_) *δ* 176.9, 168.3, 153.7, 139.5, 135.9, 131.6 (q, *J* = 33.5 Hz, 2C), 130.3 (d, *J* = 4.4 Hz), 129.7 (q, *J* = 3.7 Hz, 2C), 128.1–127.8 (m), 125.5 (q, *J* = 271.2 Hz, 2C), 125.2 (q, *J* = 3.7 Hz), 116.0, 115.8, 110.5, 30.0, 18.0 (s, 6C), 13.0 (s,3C); IR (cm^−1^) 1738, 1602, 1573, 1503, 1345, 1269, 1243, 1169, 1125, 958, 883, 803, 679, 663; HRMS (EI): Calculated for C_27_H_32_F_6_N_2_O_2_Si [M]^+^: 558.2137, Found: 558.2139.

(*Z*)-3,5-Bis(trifluoromethyl)-*N*-(6-((triisopropylsilyl)oxy)-3,4-dihydroquinolin-2(1*H*)-ylidene)benzamide (Scheme 5, **7l**): Compound **7l** was prepared from **5l** and **3i** according to the above general procedure with 19.5 h for step 1 and 3 h for step 2; eluent: DCM:hexane = 2:8; yield: 62.2 mg (23%); Yellow solid; ^1^H NMR (500 MHz, CDCl_3_) *δ* 13.11 (s, 1H), 8.74 (s, 2H), 7.99 (s, 1H), 6.84 (d, *J* = 8.4 Hz, 1H), 6.79–6.72 (m, 2H), 2.99–2.92 (m, 2H), 2.88 (ddd, *J* = 8.2, 7.0, 1.9 Hz, 2H), 1.33–1.21 (m, 3H), 1.11 (d, *J* = 7.4 Hz, 18H); ^13^C NMR (125 MHz, CDCl_3_) *δ* 176.7, 167.8, 154.1, 139.6, 131.6 (q, *J* = 33.4 Hz, 2C), 129.7 (q, *J* = 3.8 Hz, 2C), 128.3, 127.0, 125.1 (p, *J* = 3.7 Hz), 123.4 (q, *J* = 272.7 Hz, 2C), 120.0, 118.9, 118.6, 30.3, 24.2, 18.0 (s, 6C), 12.7 (s, 3C); IR (cm^−1^) 1568, 1346, 1266, 1246, 1171, 1128, 883, 800, 679, 661; HRMS (EI): Calculated for C_27_H_32_F6N_2_O_2_Si [M]^+^: 558.2137, Found: 558.2134.

(*Z*)-*N*-(6-Methoxy-3,4-dihydroquinolin-2(*1H)*-ylidene)-3,5-bis(trifluoromethyl)benzamide (Scheme 5, **7m**): Compound **7m** was prepared from **5m** and **3i** according to the above general procedure with 9 h for step 1 and 16 h for step 2; eluent: ethyl acetate:hexane = 35:65; yield: 40.5 mg (20%); White solid ^1^H NMR (500 MHz, CDCl_3_) *δ* 13.04 (s, 1H), 8.73 (s, 2H), 7.99 (s, 1H), 6.91 (d, *J* = 8.5 Hz, 1H), 6.80–6.71 (m, 2H), 3.81 (s, 3H), 2.98 (dd, *J* = 8.9, 6.3 Hz, 2H), 2.91–2.85 (m, 2H); ^13^C NMR (125 MHz, CDCl_3_) *δ* 176.6, 167.6, 157.4, 139.5, 131.5 (q, *J* = 33.6 Hz, 2C), 129.6 (d, *J* = 4.1 Hz, 2C), 128.1, 127.0, 125.2–124.8 (m), 123.3 (q, *J* = 272.7 Hz, 2C), 118.6, 114.2, 112.7, 55.5, 30.2, 24.3; IR (cm^−1^) 1585, 1567, 1503, 1343, 1278, 1240, 1162, 1119, 1044, 911, 801, 706, 699, 682; HRMS (EI): Calculated for C_19_H_14_F_6_N_2_O_2_ [M]^+^: 416.0959, Found: 416.0955.

(*Z*)-*N*-(6-(Methoxymethoxy)-3,4-dihydroquinolin-2(1*H*)-ylidene)-3,5-bis(trifluoromethyl)benzamide (Scheme 5, **7n’**): Compound **7n’** was prepared from **5n** and **3i** according to the above general procedure with 5 h for step 1 and 15 h for step 2; eluent: ethyl acetate:hexane = 15:85; yield: 106.0 mg (26%); White yellow solid; ^1^H NMR (500 MHz, CDCl_3_) *δ* 13.06 (s, 1H), 8.74–8.70 (m, 2H), 7.97 (d, *J* = 2.0 Hz, 1H), 6.94–6.86 (m, 3H), 5.14 (s, 2H), 3.47 (s, 3H), 2.97 (dd, *J* = 8.9, 6.3 Hz, 2H), 2.90–2.83 (m, 2H); ^13^C NMR (125 MHz, CDCl_3_) *δ* 176.8, 167.9, 155.1, 139.5, 131.6 (q, 2C, *J* = 33.5 Hz), 129.7 (t, 2C, *J* = 3.9 Hz), 129.2, 127.1, 125.3–125.0 (m), 123.4 (q, 2C, *J* = 272.8 Hz), 118.7, 116.5, 115.7, 94.7, 56.1, 30.3, 24.3; IR (cm^−1^) 1598, 1563, 1343, 1268, 1236, 1122, 1025, 910, 820, 798, 701, 681; HRMS (EI): Calculated for C_20_H_16_F_6_N_2_O_3_ [M]^+^: 446.1065, Found: 446.1068.

(*Z*)-*N*-(1,4-Dihydrobenzo[f]quinolin-3(2*H*)-ylidene)-3,5-bis(trifluoromethyl)benzamide (Scheme 5, **7o**): Compound **7o** was prepared from **5o** and **3i** according to the above general procedure with 16 h for step 1 and 27 h for step 2; eluent: ethyl acetate:hexane = 1:9; yield: 36.7 mg (17%); White solid; ^1^H NMR (500 MHz, CDCl_3_) *δ* 13.10 (s, 1H), 8.78–8.74 (m, 2H), 7.99 (s, 1H), 7.95 (dd, *J* = 8.5, 1.1 Hz, 1H), 7.83 (dd, *J* = 8.1, 1.3 Hz, 1H), 7.78 (d, *J* = 8.6 Hz, 1H), 7.58 (ddd, *J* = 8.4, 6.8, 1.3 Hz, 1H), 7.47 (ddd, *J* = 8.1, 6.8, 1.1 Hz, 1H), 7.14 (d, *J* = 8.6 Hz, 1H), 3.39 (dd, *J* = 8.9, 7.2 Hz, 2H), 3.05 (dd, *J* = 8.8, 7.2 Hz, 2H); ^13^C NMR (125 MHz, CDCl_3_) *δ* 175.0, 167.9, 139.3, 131.8, 131.6, 131.6 (q, *J* = 33.6 Hz, 2C), 131.2, 129.7 (d, *J* = 4.1 Hz, 2C), 128.8, 128.6, 127.3, 125.4, 125.3–125.1 (m), 123.3 (q, *J* = 272.8 Hz, 2C), 122.8, 119.2, 117.6, 30.0, 19.8; IR (cm^−1^) 1581, 1338, 1269, 1250, 1158, 1127, 937, 903, 807, 780, 743, 682; HRMS (EI): Calculated for C_22_H_14_F_6_N_2_O [M]^+^: 436.1010, Found: 436.1012.

(*Z*)-*N*-(6-Phenyl-3,4-dihydroquinolin-2(1*H*)-ylidene)-3,5-bis(trifluoromethyl)benzamide (Scheme 5, **7p**): Compound **7p** was prepared from **5p** and **3i** according to the above general procedure with 14 h for step 1 and 48 h for step 2; eluent: ethyl acetate:hexane = 1:9; yield: 110.7 mg (48%); Lemon yellow solid; ^1^H NMR (500 MHz, CDCl_3_) *δ* 13.10 (s, 1H), 8.78–8.74 (m, 2H), 8.01 (d, *J* = 2.0 Hz, 1H), 7.61–7.54 (m, 2H), 7.48 (dd, *J* = 8.1, 2.1 Hz, 1H), 7.47–7.43 (m, 3H), 7.40–7.33 (m, 1H), 7.04 (d, *J* = 8.1 Hz, 1H), 3.08 (dd, *J* = 8.8, 6.5 Hz, 2H), 2.98–2.92 (m, 2H); ^13^C NMR (125 MHz, CDCl_3_) *δ* 176.9, 168.2, 140.0, 139.3 138.7, 133.9, 131.6 (q, J = 33.7 Hz), 129.7 (d, *J* = 4.2 Hz, 2C), 128.9 (s, 2C), 127.5, 127.1, 126.8, 126.6 (s, 2C), 125.9, 125.4–125.0 (m), 122.2 (q, *J* = 274.1 Hz,2C), 117.9, 30.4, 24.1; IR (cm^−1^) 1557, 1563, 1276, 1248, 1122, 908, 816, 758, 681; HRMS (EI): Calculated for C_24_H_16_F_6_N_2_O [M]^+^: 462.1167, Found: 462.1171.

### 4.4. Crystallographic Data

CCDC 2142037 contains the supplementary crystallographic data for this paper. These data can be obtained free of charge via www.ccdc.cam.ac.uk/data_request/cif (accessed on 14 January 2022), or by emailing data_request@ccdc.cam.ac.uk (accessed on 14 January 2022), or by contacting The Cambridge Crystallographic Data Centre, 12 Union Road, Cambridge CB2 1EZ, UK; fax: +44 1223 336033.

## Supplementary Materials

The following supporting information can be downloaded, File S1: NMR spectra of all new compounds and reaction monitoring data, and X-ray crystallographic data for **4i** (PDF).

## Sample Availability

Samples of the compounds are not available from the authors.
