# Supplementary material for: Synthesis of Cyclic N-Acyl Amidines by [3 + 2] Cycloaddition of N-Silyl Enamines and Activated Acyl Azides
Source: Molecules. 2022 Mar 4;27(5):1696. doi: 10.3390/molecules27051696 (PMC8912012; doi:10.3390/molecules27051696)
Supplement: Supplementary file 1 [file molecules-27-01696-s001.zip › molecules-1617226-supplementary.pdf]

Supporting Information

Synthesis of cyclic *N*-acyl amidines by [3 + 2] cycloaddition of *N*-silyl enamines and activated acyl azides

*Dong Geun Jo, Changeun Kim, Sinjae Lee, Sooyeon Yun, and Seewon Joung**

Department of Chemistry, Mokpo National University, Jeonnam 58554, Republic of Korea

*Email: seewonjoung@gmail.com

Table of Contents

I. General Considerations……………………………………………………………………………S2

II. Synthesis of starting materials……………………………………………………………………S3

III. Reactivity of the electron withdrawing acyl azides 3 to-ward *N*-silyl enamine 2 from isoquinoline 1a (Scheme 2)……………………………………………………………………S8

IV. Substrate scope of the isoquinoline for the synthesis of acyl amidine (Scheme 3)……………S9

V. The reactivity of acyl azides 3 toward *N*-silyl enamine 6a from quinoline 5a (Scheme 4)……**S13**

VI. Substrate scope of the quinoline for the acyl amidine synthesis (Scheme 5)…………………**S14**

VII. The relative rates of hydrosilylation and [3 + 2] cycloaddition (Scheme 6)…………………**S18**

VIII. Gram scale reaction……………………………………………………………………………**S35**

IX. References ……………………………………………………………………………………**S36**

X. Copies of Spectroscopic Data …………………………………………………………………**S37**

XI. Crystallographic Data …………………………………………………………………………**S75**

I. General Considerations

Unless otherwise stated, all catalytic reactions were carried out under argon atmosphere. Chloroform-*d* purchased from Cambridge Isotope Laboratories, Inc. was degassed and used as a solvent without additional purification for optimization, substrate scope. Tris(pentafluorophenyl)borane was purchased from TCI and Acros, and was stored at -15 ^{o}C. All other reagents were directly used as purchased without further purification unless otherwise stated.

Analytical thin layer chromatography (TLC) was performed on pre-coated silica gel 60 F254 plates. Visualization on TLC was achieved by the use of UV light (254 nm), exposure to treatment with acidic *p*-anisaldehyde, phosphomolybdic acid, potassium permanganate stain followed by heating. Column chromatography was undertaken on silica gel (400-630 mesh) using a proper eluent. ^{1}H NMR was recorded on Jeol ECZ-500R (500 MHz) for characterization of compounds. Chemical shifts were quoted in parts per million (ppm) referenced to tetramethylsilane: 0.00 ppm (singlet). ^{13}C{^{1}H} NMR was recorded on Jeol ECZ-500R (125 MHz) and was fully decoupled by broad band proton decoupling. Chemical shifts were reported in ppm referenced to the center of a triplet at 77.0 ppm of $CDCl_3$. Infrared (IR) spectra were recorded on Perkin Elmer Frontier ATR-FT-IR spectrometer, ν_{max} in cm^{-1}. High resolution mass spectra were obtained by using EI and FAB method from Korea Basic Science Institute (Daegu). X-ray diffraction data was collected on a Bruker D8 QUEST coated with Parabar oil under a stream of N_2 (g) at 173 K.

II. Synthesis of starting materials[S1,S2]

Synthesis of 5-(triisopropylsilyl)oxy quinoline[S3]

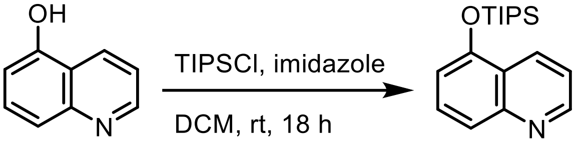

To a solution of 5-hydroxyquinoline (435 mg, 3.0 mmol, 1.0 equiv.) in DCM (7.5 mL) were added imidazole (408 mg, 6.0 mmol, 2.0 equiv.) and triisopropylsilyl chloride (0.9 mL, 4.5 mmol, 1.5 equiv.). The reaction mixture was stirred for overnight at room temperature. The resulting mixture was quenched with saturated $NaHCO_3$ solution (20 mL) and then, extracted with EtOAc (20 mL x 3). The solution was dried over $MgSO_4$, filtered, and evaporated. The crude residue was purified by a silica gel column chromatography (EtOAc/*n*-hexane = 15/85).

5-(Triisopropylsilyl)oxy quinoline (5k)

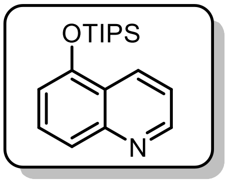

According to the above general procedure with 20 h; eluent: ethyl acetate/hexane = 2/8; yield: 841.3 mg (93%); colorless liquid; **^{1}H NMR** (500 MHz, $CDCl_3$) δ 8.90 (d, J = 4.2, 1.8 Hz, 1H), 8.58 (d, J = 8.4, 1.7, 0.9 Hz, 1H), 7.71 (d, J = 8.5 Hz, 1H), 7.54 (dd, J = 8.5, 7.6 Hz, 1H), 7.39 (dd, J = 8.4, 4.2 Hz, 1H), 6.93 (d, J = 7.8, 0.9 Hz, 1H), 1.43 (hept, J = 7.4 Hz, 3H), 1.16 (d, J = 7.5 Hz, 18H); **^{13}C NMR** (125 MHz, $CDCl_3$) δ 151.8, 150.5, 149.5, 131. 3, 129.4, 122.8, 121.9, 120.2, 112.2, 18.0 (s, 6C), 13.0 (s, 3C); **IR** (cm^{-1}) 2943, 2865, 1588, 1467, 1393, 1269, 1085, 915, 881, 795, 783 **HRMS** (EI): Calculated for $C_{18}H_{27}NOSi$ $[M]^+$: 301.1862, Found: 301.1861.

Synthesis of 6-(methoxymethoxy)quinoline[S4]

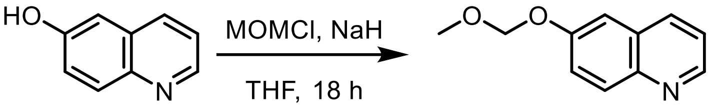

To a stirred solution of 6-hydroxyquinoline (290 mg, 2.0 mmol, 1.0 equiv.) in THF (11.6 mL) was added sodium hydride (60 wt% in mineral oil, 104 mg, 2.6 mmol, 1.3 equiv.) at 0 ℃. After hydrogen evolution was ceased, the methoxymethyl chloride ether (258 μL, 3.2 mmol, 1.6 equiv.) was added to the reaction mixture. The resulting mixture was warmed up to room temperature and stirred for 18h. The resulting mixture was quenched with water and extracted twice with CH_2Cl_2. The resulting organic layer was washed with water twice, dried over $MgSO_4$, filtered, and evaporated. The resulting crude mixture was purified by a silica gel column chromatography (acetone/DCM = 1/9).

6-(Methoxymethoxy)quinoline (5n)

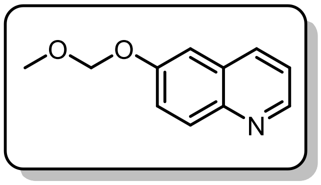

According to the above general procedure with 2 h; eluent: acetone/dichloromethane = 1/9; yield: 725.8 mg (87%); yellow liquid; **^{1}H NMR** (500 MHz, $CDCl_3$) δ 8.79 (dd, J = 4.3, 1.6 Hz, 1H), 8.07 (d, J = 8.3 Hz, 1H), 8.03 (d, J = 9.2 Hz, 1H), 7.44 (dd, J = 9.2, 2.7 Hz, 1H), 7.38 – 7.34 (m, 2H), 5.31 (s, 2H), 3.53 (s, 3H); **^{13}C NMR** (125 MHz, $CDCl_3$) δ 155.1, 148.4, 144.7, 135.1, 130.9, 129.1, 122.5, 121.3, 109.1, 94.6, 56.2; **IR** (cm^{-1}) 1486, 1121, 890, 843, 797, 782, 764, 697, 483; **HRMS** (EI): Calculated for $C_{11}H_{11}NO_2$ $[M]^+$: 189.0790, Found: 189.0789.

1n, 1o, 1p, 5l, and 5p could be synthesized according to the reported procedures of our previous works.[S1,S2]

Synthesis of carbonyl azides (3a-3i)[S5]

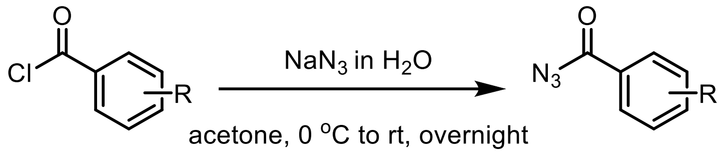

To a solution of sodium azide (910 mg, 1.4 equiv.) in water (21 mL) was added a solution of benzoyl chloride (10 mmol, 1.0 equiv.) in acetone (15 mL) dropwise at 0 ℃. The resulting mixture was warmed up to room temperature and stirred for overnight. Evaporated acetone under reduced pressure and extracted with EtOAc. The resulting organic layer was washed with water twice, dried over $MgSO_4$, filtered, and evaporated. The resulting crude mixture was purified by a silica gel column chromatography (hexane).

Benzoyl azide (3a)

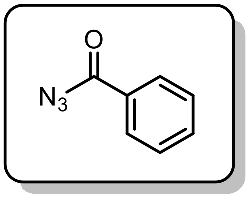

According to the above general procedure with 16 h; eluent: ethyl acetate:hexane = 1:9; yield: 1.46 g (99%); colorless liquid; **^{1}H NMR** (500 MHz, $CDCl_3$) δ 8.03 (dd, J = 8.1, 1.5 Hz, 2H), 7.65 – 7.58 (m, 1H), 7.49 – 7.42 (m, 2H).; **^{13}C NMR** (125 MHz, $CDCl_3$) δ 172.5, 134.3, 130.6, 129.4 (s, 2C), 128.6 (s, 2C); **IR** (cm^{-1}) 2130, 1690, 1233, 1173, 983, 693; **HRMS** (EI): Calculated for $C_7H_5N_3O$ $[M]^+$: 147.0433, Found: 147.0434.

4-Bromobenzoyl azide (3b)

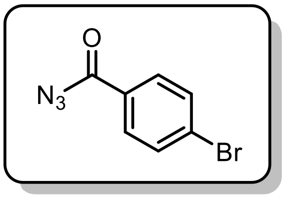

According to the above general procedure with 16 h; eluent: ethyl acetate:hexane = 5:95; yield: 2.03 g (90%); white yellow solid; **^{1}H NMR** (500 MHz, $CDCl_3$) δ 7.92 – 7.85 (m, 2H), 7.64 – 7.57 (m, 2H); **^{13}C NMR** (125 MHz, $CDCl_3$) δ 171.8, 132.0 (s, 2C), 130.9 (s, 2C), 129.7, 129.5; **IR** (cm^{-1}) 2129, 1675, 1580, 1397, 1232, 1989, 839, 738, 675, 466; **HRMS** (EI): Calculated for $C_7H_4BrN_3O$ $[M]^+$: 224.9538, Found: 224.9535

4-Chlorobenzoyl azide (3c)

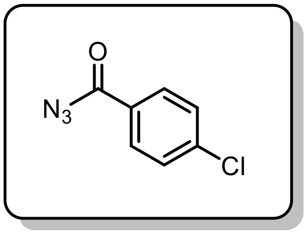

According to the above general procedure with 16 h; eluent: ethyl acetate:hexane = 2:98; yield: 1.80 g (99%); white yellow solid; **^{1}H NMR** (500 MHz, $CDCl_3$) δ 7.99 – 7.93 (m, 2H), 7.47 – 7.40 (m, 2H); **^{13}C NMR** (125 MHz, $CDCl_3$) δ 171.6, 141.0, 130.8 (s, 2C), 129.0 (s, 3C); **IR** (cm^{-1}) 2174, 2132, 1678, 1250, 1086, 1166, 991, 841, 742, 675; **HRMS** (EI): Calculated for $C_7H_4ClN_3O$ $[M]^+$: 181.0043, Found: 181.0041.

4-Fluorobenzoyl azide (3d)

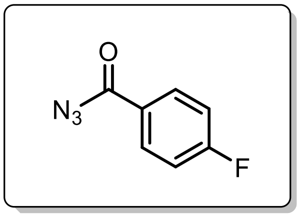

According to the above general procedure with 16 h; eluent: ethyl acetate:hexane = 2:98; yield: 1.63 g (99%); colorless liquid; **^{1}H NMR** (500 MHz, $CDCl_3$) δ 8.09 – 8.02 (m, 2H), 7.17 – 7.09 (m, 2H); **^{13}C NMR** (125 MHz, $CDCl_3$) δ 171.3, 167.6, 165.6, 132.1 (d, 2C, J = 9.6 Hz), 126.9 (d, 2C, J = 2.9 Hz), 115.8; **IR** (cm^{-1}) 2132, 1685, 1596, 1505, 1230, 1170, 1151, 988, 848, 748, 675, 615, 501; **HRMS** (EI): Calculated for $C_7H_4FN_3O$ $[M]^+$: 165.0338, Found: 165.0337.

4-Methoxybenzoyl azide (3e)

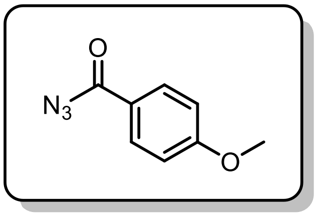

According to the above general procedure with 16 h; eluent: ethyl acetate:hexane = 1:9; yield: 1.43 g (81%); White yellow solid; **^{1}H NMR** (500 MHz, $CDCl_3$) δ 8.02 – 7.95 (m, 2H), 6.96 – 6.89 (m, 2H), 3.87 (s, 3H); **^{13}C NMR** (125 MHz, $CDCl_3$) δ 171.7, 164.6, 131.7(s, 2C), 123.1, 113.9(s, 2C), 55.5; **IR** (cm^{-1}) 2134, 1677, 1247, 1196, 1167, 1011, 984, 846, 752, 684, 618, 509; **HRMS** (EI): Calculated for $C_8H_7N_3O_2$ $[M]^+$: 177.0538, Found: 177.0540.

4-Nitrobenzoyl azide (3f)

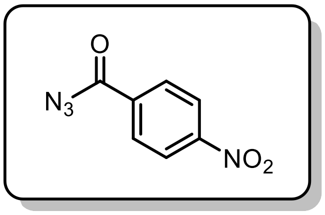

According to the above general procedure with 16.5 h; eluent: ethyl acetate:hexane = 25:75; yield: 1806.1 mg (94%); yellow solid; **^{1}H NMR** (500 MHz, $CDCl_3$) δ 8.35 – 8.29 (m, 2H), 8.27 – 8.19 (m, 2H); **^{13}C NMR** (125 MHz, $CDCl_3$) δ 170.9, 151.2, 135.7, 130.5 (s, 2C), 123.8 (s, 2C); **IR** (cm^{-1}) 2181, 2139, 1693, 1537, 1350, 1228, 1179, 990, 844, 707; **HRMS** (EI): Calculated for $C_7H_4N_4O_3$ $[M]^+$: 192.0283, Found: 192.0284.

3,5-dinitrobenzoyl azide (3g)

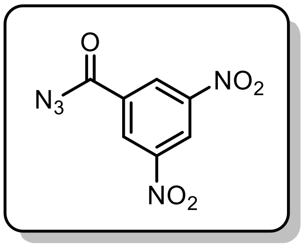

According to the above general procedure with 1 h and 20 mmol scale eluent: DCM:hexane = 4:6; yield: 3.74 g (79%); white yellow solid; **^{1}H NMR** (500 MHz, $CDCl_3$) δ 9.28 (t, J = 2.2 Hz, 1H), 9.17 (d, J = 2.1 Hz, 2H); **^{13}C NMR** (125 MHz, $CDCl_3$) δ 168.7, 148.8, 134.0, 129.1 (s, 2C), 123.3 (s, 2C); **IR** (cm^{-1}) 3103, 2151, 1686, 1537, 1345, 1255, 1164, 921, 729, 712; **HRMS** (EI): Calculated for $C_7H_5N_3O_5$ $[M-N_2+H_2]^+$: 211.0229, Found: 211.0228.

4-(Trifluoromethyl)benzoyl azide (3h)

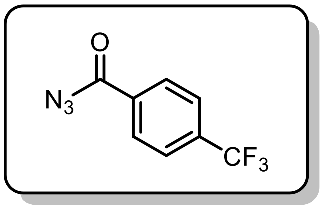

According to the above general procedure with 16 h and 8 mmol scale eluent: ethyl acetate:hexane = 25:75; yield: 1.33 g (77%); white solid; **^{1}H NMR** (500 MHz, $CDCl_3$) δ 8.15 (d, J = 8.1 Hz, 2H), 7.73 (d, J = 8.2 Hz, 2H); **^{13}C NMR** (125 MHz, $CDCl_3$) δ 171.5, 135.6 (q, J = 32.9 Hz), 133.6, 129.8 (s, 2C), 125.7 (q, 2C, J = 3.7 Hz), 123.4 (q, J = 272.9 Hz); **IR** (cm^{-1}) 2135, 1691, 1412, 1321, 1249, 1166, 1108, 1063, 991, 856, 762, 687; **HRMS** (EI): Calculated for $C_8H_4F_3N_3O$ $[M]^+$: 215.0306, Found: 215.0303.

3,5-Bis(trifluoromethyl)benzoyl azide (3i)

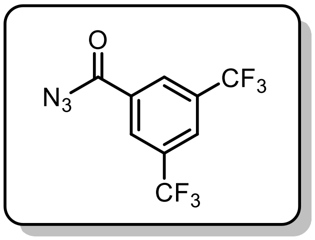

According to the above general procedure with 16 h; eluent: ethyl acetate:hexane = 25:75; yield: 2.37 g (83.8%); colorless liquid; **^{1}H NMR** (500 MHz, $CDCl_3$) δ 8.47 (d, J = 1.6 Hz, 2H), 8.10 (s, 1H).; **^{13}C NMR** (125 MHz, $CDCl_3$) δ 170.1, 132.6, 132.5 (q, 2C, J = 34.3 Hz), 129.5 (q, 2C, J = 3.7 Hz), 127.6 – 127.3 (m), 122.7 (q, 2C, J = 273.1 Hz); **IR** (cm^{-1}) 2136, 1694, 1599, 1278, 1214, 1130, 993, 913, 850, 782, 744, 706, 680, 616; **HRMS** (EI): Calculated for $C_9H_3F_6N_3O$ $[M]^+$: 283.0180, Found: 283.0183.

III. Reactivity of the electron withdrawing acyl azides 3 toward *N*-silyl enamine 2 from isoquinoline 1a (Scheme 2)

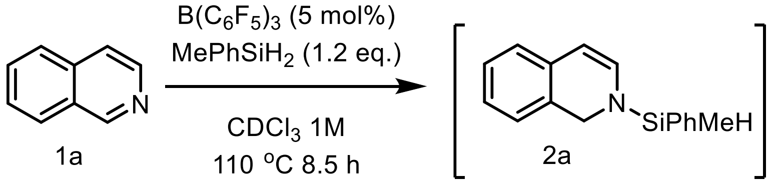

Step 1: To a $B(C_6F_5)_3$ catalyst (0.025 mmol, 5 mol%) in NMR tube was added $CDCl_3$ (0.5 mL) and silanes (0.6 mmol, 1.2 equiv.) at room temperature, H_2 bubbles were observed and TCE (0.3 mmol) or mesitylene was added as internal standard. Isoquinoline **1a** (0.5 mmol, 1.0 equiv.) was subsequently added to the above solution and quickly shaken once before heating up to 110 °C in oil bath for 8.5 h. The mixture was subjected to NMR to check conversion and yields of reactions.

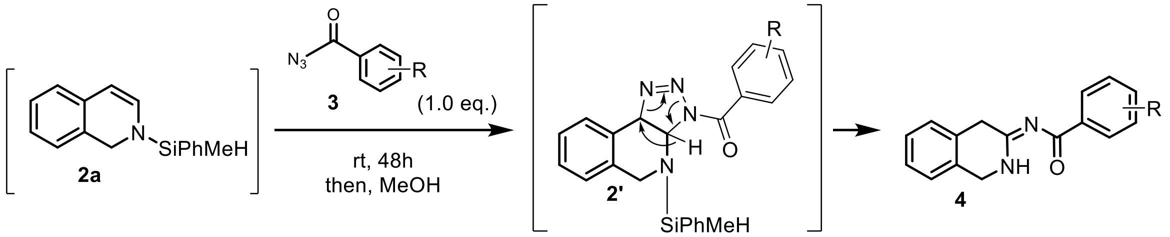

Step 2: In the crude reaction mixture from the first step was added acyl azide (**3a-3i**) (0.5 mmol, 1.0 equiv.) at room temperature and took NMR after 48 h. The resulting mixture was quenched by MeOH addition, silica filter, and DCM wash. The resulting crude mixture was subjected to NMR to check crude yields of reactions.

IV. Substrate scope of the isoquinoline for the synthesis of acyl amidine (Scheme 3)

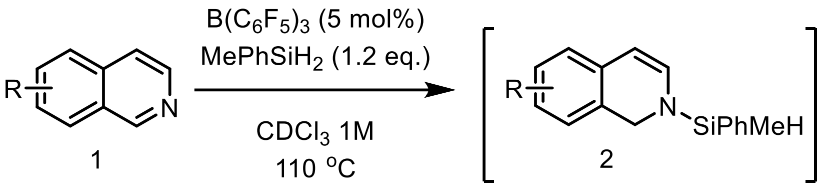

Step 1: To a $B(C_6F_5)_3$ catalyst (0.025 mmol, 5 mol%) in NMR tube was added $CDCl_3$ (0.5 mL) and silanes (0.6 mmol, 1.2 equiv.) at room temperature, H_2 bubbles were observed and TCE (0.3 mmol) or mesitylene was added as internal standard. Isoquinolines (**1a, 1i-1p**) (0.5 mmol, 1.0 equiv.) was subsequently added to the above solution and quickly shaken once before heating up to 110 °C in oil bath for indicated reaction time. The mixture was subjected to NMR to check conversion and yields of reactions.

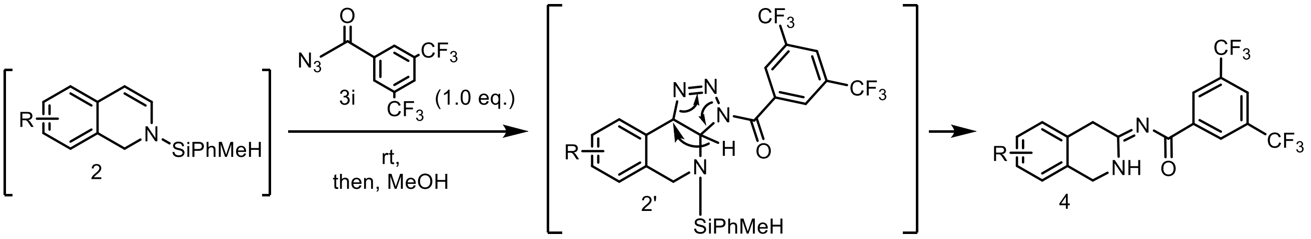

Step 2: In the crude reaction mixture from the first step was added acyl azide **3i** (0.5 mmol, 1.0 equiv.) at room temperature and took NMR in indicated reaction time. The resulting mixture was quenched by MeOH addition, silica filter, and DCM wash. The resulting crude mixture was purified by column chromatography.

(*Z*)-*N*-(1,4-Dihydroisoquinolin-3(2*H*)-ylidene)-3,5-bis(trifluoromethyl)benzamide (Scheme 3, 4i)

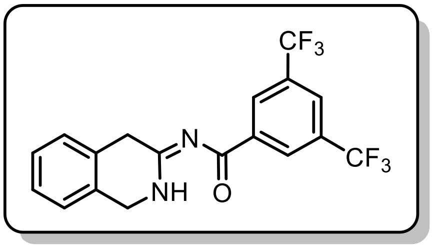

Compound **4i** was prepared from **1a** and **3i** according to the above general procedure with 8.5 h for step 1 and 16 h for step 2; eluent: ethyl acetate:hexane = 2:8; yield: 193.1 mg (73%); yellowish solid; **^{1}H NMR** (500 MHz, $CDCl_3$) δ 11.92 (s, 1H), 8.64 (s, 2H), 7.88 (s, 1H), 7.27 – 7.18 (m, 3H), 7.17 – 7.13 (m, 1H), 4.56 (t, J = 2.2 Hz, 2H), 3.78 (t, J = 2.3 Hz, 2H); **^{13}C NMR** (125 MHz, $CDCl_3$) δ 176.5, 171.1, 139.7, 131.4 (q, 2C, J = 33.6 Hz), 130.6, 130.2, 129.5 (2C), 128.1, 127.8, 127.2, 125.5, 124.9 (p, J = 3.5 Hz), 123.4 (q, 2C, J = 272.8 Hz), 45.3, 36.6; **IR** (cm^{-1}) 1738, 1607, 1488, 1312, 1281, 1119, 911, 742, 682; **HRMS** (EI): Calculated for $C_{18}H_{12}F_6N_2O$ $[M]^+$: 386.0854, Found: 386.0851.

(*Z*)-*N*-(5-Chloro-1,4-dihydroisoquinolin-3(2*H*)-ylidene)-3,5-bis(trifluoromethyl)benzamide (Scheme 3, 4j)

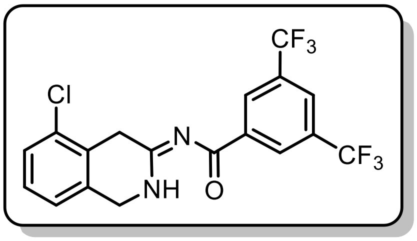

Compound **4j** was prepared from **1j** and **3i** according to the above general procedure with 2.5 h for step 1 and 68 h for step 2; eluent: ethyl acetate:hexane = 2:8; yield: 156 mg (69%); White yellow solid; **^{1}H NMR** (500 MHz, $CDCl_3$) δ 12.12 (s, 1H), 8.73 (d, J = 1.8 Hz, 2H), 7.98 (s, 1H), 7.38 (dd, J = 8.0, 1.1 Hz, 1H), 7.24 (d, J = 7.8 Hz, 1H), 7.14 (d, J = 7.6 Hz, 1H), 4.69 (t, J = 2.4 Hz, 2H), 3.94 (t, J = 2.4 Hz, 2H); **^{13}C NMR** (125 MHz, $CDCl_3$) δ 176.6, 169.9, 139.6, 133.4, 131.5, 131.4 (q, 2C, J = 33.4 Hz), 129.5 (d, 2C, J = 3.7 Hz), 128.6, 128.4, 128.2, 124.9 (p, J = 3.5 Hz), 123.8, 123.3 (q, 2C, J = 272.8 Hz), 45.2, 33.5; **IR** (cm^{-1}) 1614, 1578, 1287, 1111, 911, 777, 701, 681; **HRMS** (EI): Calculated for $C_{18}H_{11}ClF_6N_2O$ $[M]^+$: 420.0464, Found: 420.0460.

(*Z*)-*N*-(8-Bromo-1,4-dihydroisoquinolin-3(2*H*)-ylidene)-3,5-bis(trifluoromethyl)benzamide (Scheme 3, 4k)

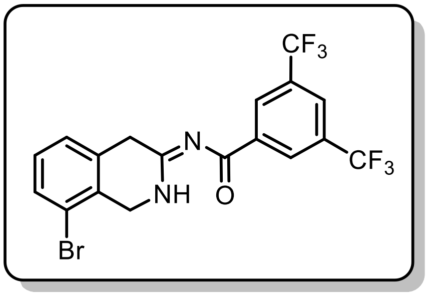

Compound **4k** was prepared from **1k** and **3i** according to the above general procedure with 2.5 h for step 1 and 48 h for step 2; eluent: ethyl acetate:hexane = 1:9; yield: 107.0 mg (55%); White yellow solid; **^{1}H NMR** (500 MHz, $CDCl_3$) δ 12.03 (s, 1H), 8.72 (s, 2H), 7.98 (s, 1H), 7.53 (dd, J = 7.6, 1.5 Hz, 1H), 7.26 – 7.19 (m, 2H), 4.73 (t, J = 2.3 Hz, 2H), 3.90 (t, J = 2.3 Hz, 2H); **^{13}C NMR** (125 MHz, $CDCl_3$) δ 176.6, 170.1, 139.5, 132.6, 131.4 (q, 2C, J = 33.4 Hz), 131.2, 129.8, 129.5 (q, 2C, J =2.75 Hz), 129.4, 126.9, 125.2 – 124.8 (m), 123.3 (q, 2C, J = 272.7 Hz), 121.4, 45.8, 36.2; **IR** (cm^{-1}) 1603, 1329, 1283, 1244, 1121, 774, 682; **HRMS** (EI): Calculated for $C_{18}H_{11}BrF_6N_2O$ $[M]^+$: 463.9959, Found: 463.9956.

(*Z*)-*N*-(5-Bromo-1,4-dihydroisoquinolin-3(2*H*)-ylidene)-3,5-bis(trifluoromethyl)benzamide (Scheme 3, 4l)

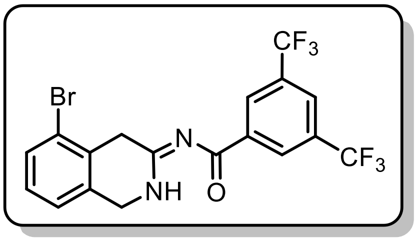

Compound **4l** was prepared from **1l** and **3i** according to the above general procedure with 2.5 h for step 1 and 48 h for step 2; eluent: ethyl acetate:hexane = 2:8; yield: 164.8 mg (70.9%); yellowish solid; **^{1}H NMR** (500 MHz, $CDCl_3$) δ 12.13 (s, 1H), 8.74 (s, 2H), 7.99 (s, 1H), 7.60 (dd, J = 5.5, 3.7 Hz, 1H), 7.20 (s, 1H), 7.19 (d, J = 1.9 Hz, 1H), 4.71 (s, 2H), 3.95 (t, J = 2.4 Hz, 2H); **^{13}C NMR** (125 MHz, $CDCl_3$) δ 176.7, 170.2, 139.6, 132.0, 131.7, 131.5 (q, 2C, J = 33.4 Hz), 130.2, 129.6 (d, 2C, J = 4.0 Hz), 128.6, 125.1 – 124.8 (m), 124.6, 124.4 (q, 2C, J = 274 Hz), 123.8, 45.4, 36.4; **IR** (cm^{-1}) 1614, 1487, 1325, 1285, 1164, 1119, 1109, 911,

885, 682; **HRMS** (EI): Calculated for $C_{18}H_{11}BrF_6N_2O$ $[M]^+$: 463.9959, Found: 463.9961.

(*Z*)-*N*-(7-Bromo-1,4-dihydroisoquinolin-3(2*H*)-ylidene)-3,5-bis(trifluoromethyl)benzamide (Scheme 3, 4m)

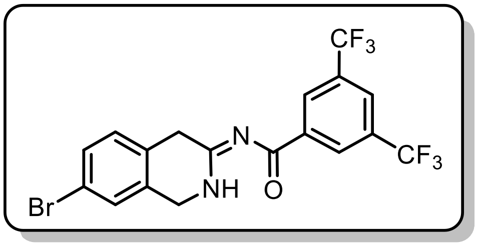

Compound **4m** was prepared from **1m** and **3i** according to the above general procedure with 2.5 h for step 1 and 48 h for step 2; eluent: DCM:hexane = 8:2; yield: 171.6 mg (74%); White yellow solid; **^{1}H NMR** (500 MHz, $CDCl_3$) δ 12.00 (s, 1H), 8.72 (s, 2H), 7.98 (s, 1H), 7.47 (dd, J = 8.1, 2.0 Hz, 1H), 7.42 (d, J = 1.9 Hz, 1H), 7.18 (d, J = 8.1 Hz, 1H), 4.62 (s, 2H), 3.81 (s, 2H); **^{13}C NMR** (125 MHz, $CDCl_3$) δ 176.6, 170.4, 139.5, 132.3, 131.4 (q, 2C, J = 33.6 Hz), 131.2, 129.6, 129.5 (q, 2C, J = 2.6 Hz), 129.4, 128.5, 125.1 – 124.8 (m, J = 3.7 Hz), 124.4 (q, 2C, J = 270.5 Hz), 120.9, 44.8, 36.1; **IR** (cm^{-1})1613, 1584, 1334, 1268, 1122, 841, 699, 680; **HRMS** (EI): Calculated for $C_{18}H_{11}BrF_6N_2O$ $[M]^+$: 463.9959, Found: 463.9956.

(*Z*)-3,5-Bis(trifluoromethyl)-*N*-(5-((trimethylsilyl)ethynyl)-1,4-dihydroisoquinolin-3(2*H*)-ylidene)benzamide (Scheme 3, 4n)

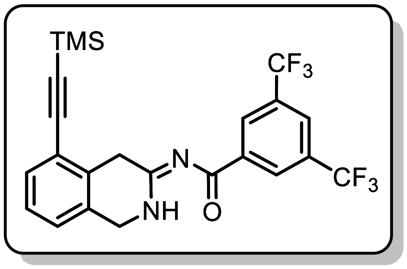

Compound **4n** was prepared from **1n** and **3i** according to the above general procedure in 8.5 h for step 1 and 68 h for step 2; eluent: ethyl acetate:hexane = 15:85; yield: 170.7 mg (71%); Yellow solid; **^{1}H NMR** (500 MHz, $CDCl_3$) δ 12.06 (s, 1H), 8.75 (s, 2H), 7.98 (s, 1H), 7.48 (dd, J = 7.7, 1.4 Hz, 1H), 7.24 (d, J = 7.7 Hz, 1H), 7.18 (d, J = 7.6, 1.3 Hz, 1H), 4.65 (s, 2H), 4.01 (s, 2H), 0.34 (s, 9H); **^{13}C NMR** (125 MHz, $CDCl_3$) δ 176.5, 170.6, 139.6, 132.4, 131.7, 131.4 (q, 2, J = 33.4 Hz), 130.1, 129.5 (q, 2C, J = 3.8 Hz), 126.9, 125.5, 124.9 (p, J = 3.6 Hz), 123.3 (q, 2C, J = 272.8 Hz), 122.4, 101.7, 101.1, 45.2, 34.8, -0.1 (s, 3C); **IR** (cm^{-1}) 1610, 1310, 1278, 1174, 1111, 842, 761, 696, 680; **HRMS** (EI): Calculated for $C_{23}H_{20}F_6N_2OSi$ $[M]^+$: 482.1249, Found: 482.1253.

(*Z*)-3,5-Bis(trifluoromethyl)-*N*-(5-((triisopropylsilyl)oxy)-1,4-dihydroisoquinolin-3(2*H*)-ylidene)benzamide (Scheme 3, 4o)

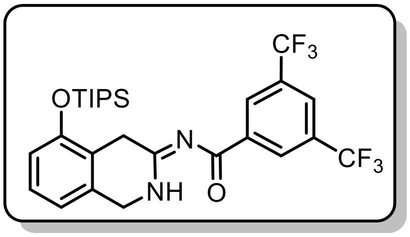

Compound **4o** was prepared from **1o** and **3i** according to the above general procedure with 20 h for step 1 and 24 h for step 2; eluent: ethyl acetate:hexane = 1:3; yield: 122.5 mg (45%); Yellow solid; **^{1}H NMR** (500 MHz, $CDCl_3$) δ 12.05 (s, 1H), 8.73 (s, 2H), 7.97 (s, 1H), 7.15 (t, J = 7.9 Hz, 1H), 6.82 (d, 1H), 6.80 (d, 1H), 4.65 (t, J = 2.5 Hz, 2H), 3.83 (t, J = 2.5 Hz, 2H), 1.44 – 1.32 (m, 3H), 1.16 (d, J = 7.5 Hz, 18H); **^{13}C NMR** (125 MHz, $CDCl_3$) δ 176.5, 171.1, 153.3, 139.9, 131.3 (q, 2C, J = 33.7 Hz), 131.2, 129.5 (s, 2C), 127.7, 124.8, 123.4

(q, 2C, J = 272.6 Hz), 120.9, 117.6, 116.9, 45.2, 30.9, 18.0 (s, 6C), 13.0 (s,3C); **IR** (cm^{-1}) 1615, 1587, 1463, 1311, 1273, 1127, 881, 771, 680; **HRMS** (EI): Calculated for $C_{27}H_{32}F_6N_2O_2Si$ $[M]^+$: 558.2137, Found: 558.2139.

(*Z*)-3,5-Bis(trifluoromethyl)-*N*-(7-((triisopropylsilyl)oxy)-1,4-dihydroisoquinolin-3(2*H*)-ylidene)benzamide (Scheme 3, 4p)

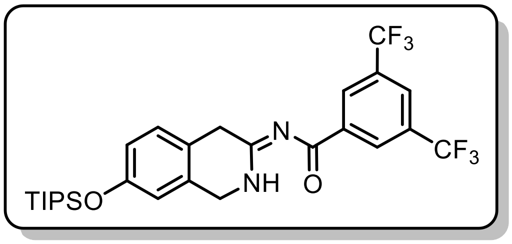

Compound **4p** was prepared from **1p** and **3i** according to the above general procedure with 42 h for step 1 and 48 h for step 2; eluent: acetone:hexane = 1:3; yield: 55.8 mg (20%); Yellow solid; **^{1}H NMR** (500 MHz, $CDCl_3$) δ 12.01 (s, 1H), 8.72 (s, 2H), 7.97 (s, 1H), 7.13 (d, J = 8.3 Hz, 1H), 6.85 (dd, J = 8.3, 2.5 Hz, 1H), 6.76 (d, J = 2.4 Hz, 1H), 4.58 (s, 2H), 3.79 (s, 2H), 1.31 – 1.21 (m, 3H), 1.11 (d, J = 7.4 Hz, 18H); **^{13}C NMR** (125 MHz, $CDCl_3$) δ 176.5, 171.5, 155.3, 139.8, 131.4 (q, 2C, J = 33.5 Hz), 131.2, 129.5 (d, 2C, J = 4.2 Hz), 128.7, 125.0 – 124.6 (m), 123.4 (q, 2C, J = 272.8 Hz), 122.6, 119.7, 116.6, 45.3, 35.8, 17.9 (s, 6C), 12.6 (s, 3C); **IR** (cm^{-1}) 1607, 1274, 1130, 971, 881, 821, 680; **HRMS** (EI): Calculated for $C_{27}H_{32}F_6N_2O_2Si$ $[M]^+$: 558.2137, Found: 558.2134.

V. The reactivity of acyl azides 3 toward *N*-silyl enamine 6a from quinoline 5a (Scheme 4)

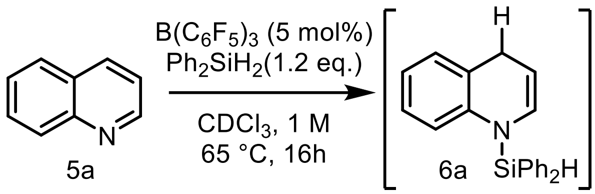

Step 1: To a $B(C_6F_5)_3$ catalyst (0.025 mmol, 5 mol%) in NMR tube was added $CDCl_3$ (0.5 mL) and silanes (0.6 mmol, 1.2 equiv.) at room temperature, H_2 bubbles were observed and TCE (0.3 mmol) or mesitylene was added as internal standard. Quinoline **5a** (0.5 mmol, 1.0 equiv.) was subsequently added to the above solution and quickly shaken once before heating up to 65 °C in oil bath for 16 h. The mixture was subjected to NMR to check conversion and yields of reactions.

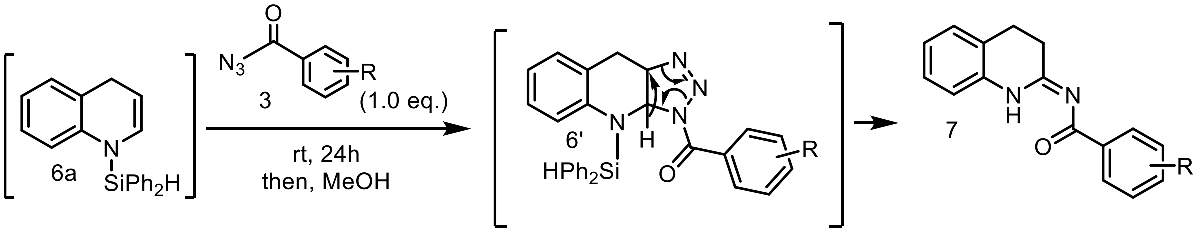

Step 2: In the crude reaction mixture from the first step was added acyl azide (**3a-3i**) (0.5 mmol, 1.0 equiv.) at room temperature and took NMR after 24 h. The resulting mixture was quenched by MeOH addition, silica filter, and DCM wash. The resulting crude mixture was subjected to NMR to check crude yields of reactions.

VI. Substrate scope of the quinoline for the acyl amidine synthesis (Scheme 5)

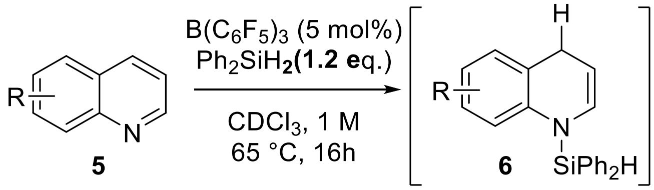

Step 1: To a $B(C_6F_5)_3$ catalyst (0.025 mmol, 5 mol%) in NMR tube was added $CDCl_3$ (0.5 mL) and silanes (0.6 mmol, 1.2 equiv.) at room temperature, H_2 bubbles were observed and TCE (0.3 mmol) or mesitylene was added as internal standard. Quinoline (**5i-5q**) (0.5 mmol, 1.0 equiv.) was subsequently added to the above solution and quickly shaken once before heating up to 65 °C in oil bath for indicated reaction time. The mixture was subjected to NMR to check conversion and yields of reactions.

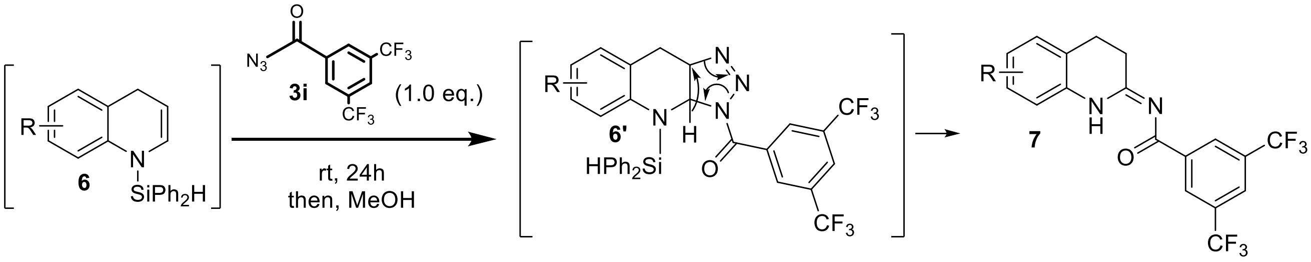

Step 2: In the crude reaction mixture from the first step was added acyl azide **3i** (0.5 mmol, 1.0 equiv.) at room temperature and took NMR in indicated reaction time. The resulting mixture was quenched by MeOH addition, silica filter, and DCM wash. The resulting crude mixture was purified by column chromatography.

(*Z*)-*N*-(3,4-Dihydroquinolin-2(1*H*)-ylidene)-3,5-bis(trifluoromethyl)benzamide (Scheme 5, 7i)

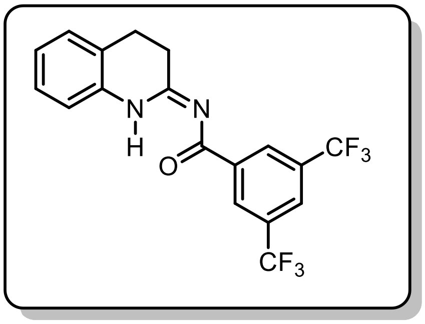

Compound **7i** was prepared from **5i** and **3i** according to the above general procedure with 16 h for step 1 and 24 h for step 2; eluent: ethyl acetate:hexane = 5:95; yield: 799.0 mg (41%); White solid; **^{1}H NMR** (500 MHz, $CDCl_3$) δ 13.03 (s, 1H), 8.75 (s, 2H), 8.00 (s, 1H), 7.27 (td, J = 7.4, 1.2 Hz, 1H), 7.22 (d, J = 7.5 Hz, 1H), 7.14 (td, J = 7.4, 1.2 Hz, 1H), 6.98 (d, J = 7.8 Hz, 1H), 3.06 – 2.99 (m, 2H), 2.95 – 2.89 (m, 2H); **^{13}C NMR** (125 MHz, $CDCl_3$) δ 176.8, 168.4, 139.3, 134.7, 131.5 (q, J = 33.5 Hz, 2C), 129.7 (q, J = 3.8 Hz, 2C), 128.4, 127.9, 125.6, 125.5, 125.2 (p, J = 3.8 Hz), 123.3 (q, J = 272.7 Hz, 2C), 117.6, 30.3, 23.9; **IR** (cm^{-1}) 1573, 1349, 1281, 1268, 1254, 1119, 908,756, 700, 683; **HRMS** (EI): Calculated for $C_{18}H_{12}F_6N_2O$ $[M]^+$: 386.0854, Found: 386.0852.

(*Z*)-*N*-(6-Methyl-3,4-dihydroquinolin-2(1*H*)-ylidene)-3,5-bis(trifluoromethyl)benzamide (Scheme 5, 7j)

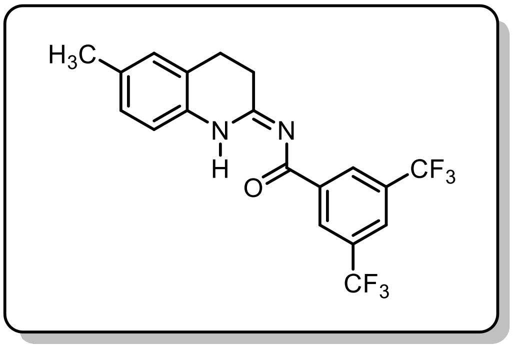

Compound **7j** was prepared from **5j** and **3i** according to the above general procedure with 20 h for step 1 and 24 h for step 2; eluent: ethyl acetate:hexane = 5:95; yield: 93.1 mg (47%); Yellow solid; **^{1}H NMR** (500 MHz, $CDCl_3$) δ 13.03 (s, 1H), 8.73 (s, 2H), 7.98 (s, 1H), 7.03 (dd, J = 8.0, 1.9 Hz, 1H), 7.00 (s, 1H), 6.85 (d, J = 7.9 Hz, 1H), 3.00 – 2.93 (m, 2H), 2.91 – 2.86 (m, 2H), 2.32 (s, 3H); **^{13}C NMR** (125 MHz, $CDCl_3$) δ 176.7, 168.1, 139.4, 135.5, 132.2, 131.5 (q, J = 33.5 Hz, 2C), 129.6 (q, J = 3.8 Hz, 2C), 129.1, 128.3, 125.3, 125.0 (p, J = 3.8 Hz), 123.3 (q, J = 272.8 Hz, 2C), 117.4, 30.4, 23.9, 20.9.; **IR** (cm^{-1}) 1605, 1566, 1343, 1268, 1233,1252, 1163, 1120, 909, 808, 682; **HRMS** (EI): Calculated for $C_{19}H_{14}F_6N_2O$ $[M]^+$: 400.1010, Found: 400.1012.

(*Z*)-3,5-Bis(trifluoromethyl)-*N*-(5-((triisopropylsilyl)oxy)-3,4-dihydroquinolin-2(1*H*)-ylidene)benzamide (Scheme 5, 7k)

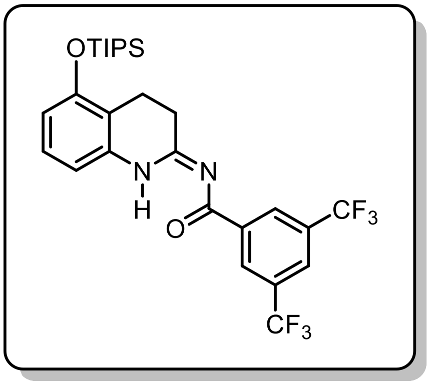

Compound **7k** was prepared from **5k** and **3i** according to the above general procedure with 48 h for step 1 and 18 h for step 2; eluent: ethyl acetate:hexane = 5:95; yield: 89.2 mg (32%); Yellow liquid; **^{1}H NMR** (500 MHz, $CDCl_3$) δ 12.98 (s, 1H), 8.76 (s, 2H), 8.00 (s, 1H), 7.09 (t, J = 8.0 Hz, 1H), 6.67 (dd, J = 8.3, 1.0 Hz, 1H), 6.59 (d, J = 2 Hz, 1H), 3.01 (dd, J = 8.8, 6.8 Hz, 2H), 2.87 (dd, J = 2, 2.2 Hz 2H), 1.39 – 1.23 (m, 3H), 1.13 (d, J = 7.5 Hz, 18H).; **^{13}C NMR** (125 MHz, $CDCl_3$) δ 176.9, 168.3, 153.7, 139.5, 135.9, 131.6 (q, J = 33.5 Hz, 2C), 130.3 (d, J = 4.4 Hz), 129.7 (q, J = 3.7 Hz, 2C), 128.1 – 127.8 (m), 125.5 (q, J = 271.2 Hz, 2C), 125.2 (q, J = 3.7 Hz), 116.0, 115.8, 110.5, 30.0, 18.0 (s, 6C), 13.0 (s,3C); **IR** (cm^{-1}) 1738, 1602, 1573, 1503, 1345, 1269, 1243, 1169, 1125, 958, 883, 803, 679, 663; **HRMS** (EI): Calculated for $C_{27}H_{32}F_6N_2O_2Si$ $[M]^+$: 558.2137, Found: 558.2139.

(*Z*)-3,5-Bis(trifluoromethyl)-*N*-(6-((triisopropylsilyl)oxy)-3,4-dihydroquinolin-2(1*H*)-ylidene)benzamide (Scheme 5, 7l)

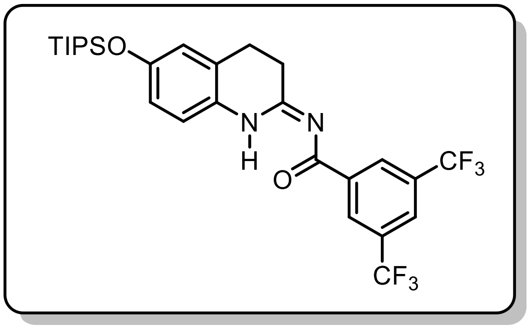

Compound **7l** was prepared from **5l** and **3i** according to the above general procedure with 19.5 h for step 1 and 3 h for step 2; eluent: DCM:hexane = 2:8; yield: 62.2 mg (23%); Yellow solid; **^{1}H NMR** (500 MHz, $CDCl_3$) δ 13.11 (s, 1H),

8.74 (s, 2H), 7.99 (s, 1H), 6.84 (d, J = 8.4 Hz, 1H), 6.79 – 6.72 (m, 2H), 2.99 – 2.92 (m, 2H), 2.88 (ddd, J = 8.2, 7.0, 1.9 Hz, 2H), 1.33 – 1.21 (m, 3H), 1.11 (d, J = 7.4 Hz, 18H); **^{13}C NMR** (125 MHz, $CDCl_3$) δ 176.7, 167.8, 154.1, 139.6, 131.6 (q, J = 33.4 Hz, 2C), 129.7 (q, J = 3.8 Hz, 2C), 128.3, 127.0, 125.1 (p, J = 3.7 Hz), 123.4 (q, J = 272.7 Hz, 2C), 120.0, 118.9, 118.6, 30.3, 24.2, 18.0 (s, 6C), 12.7 (s, 3C); **IR** (cm^{-1}) 1568, 1346, 1266, 1246, 1171, 1128, 883, 800, 679, 661; **HRMS** (EI): Calculated for $C_{27}H_{32}F6N_2O_2Si$ $[M]^+$: 558.2137, Found: 558.2134.

(*Z*)-*N*-(6-Methoxy-3,4-dihydroquinolin-2(*1H)*-ylidene)-3,5-bis(trifluoromethyl)benzamide (Scheme 5, 7m)

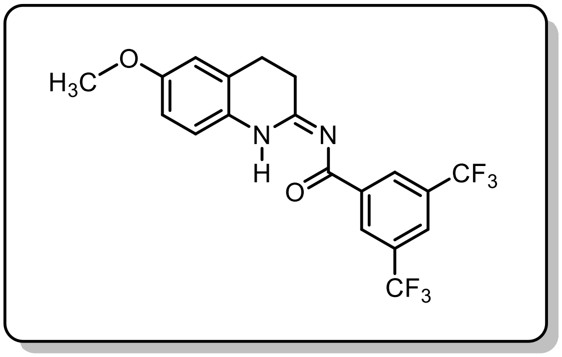

Compound **7m** was prepared from **5m** and **3i** according to the above general procedure with 9 h for step 1 and 16 h for step 2; eluent: ethyl acetate:hexane = 35:65; yield: 40.5 mg (20%); White solid **^{1}H NMR** (500 MHz, $CDCl_3$) δ 13.04 (s, 1H), 8.73 (s, 2H), 7.99 (s, 1H), 6.91 (d, J = 8.5 Hz, 1H), 6.80 – 6.71 (m, 2H), 3.81 (s, 3H), 2.98 (dd, J = 8.9, 6.3 Hz, 2H), 2.91 – 2.85 (m, 2H); **^{13}C NMR** (125 MHz, $CDCl_3$) δ 176.6, 167.6, 157.4, 139.5, 131.5 (q, J = 33.6 Hz, 2C), 129.6 (d, J = 4.1 Hz, 2C), 128.1, 127.0, 125.2 – 124.8 (m), 123.3 (q, J = 272.7 Hz, 2C), 118.6, 114.2, 112.7, 55.5, 30.2, 24.3; **IR** (cm^{-1}) 1585, 1567, 1503, 1343, 1278, 1240, 1162, 1119, 1044, 911, 801, 706, 699, 682; **HRMS** (EI): Calculated for $C_{19}H_{14}F_6N_2O_2$ $[M]^+$: 416.0959, Found: 416.0955.

(*Z*)-*N*-(6-(Methoxymethoxy)-3,4-dihydroquinolin-2(1*H*)-ylidene)-3,5-bis(trifluoromethyl)benzamide (Scheme 5, 7n')

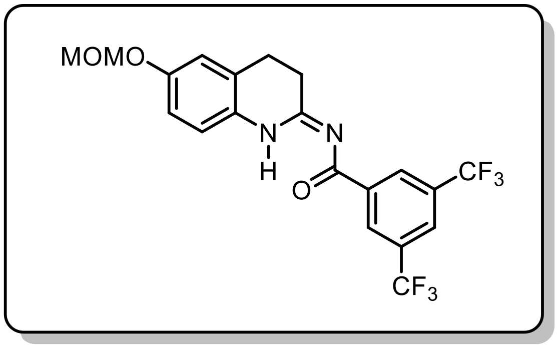

Compound **7n'** was prepared from **5n** and **3i** according to the above general procedure with 5 h for step 1 and 15 h for step 2; eluent: ethyl acetate:hexane = 15:85; yield: 106.0 mg (26%); White yellow solid; **^{1}H NMR** (500 MHz, $CDCl_3$) δ 13.06 (s, 1H), 8.74 – 8.70 (m, 2H), 7.97 (d, J = 2.0 Hz, 1H), 6.94 – 6.86 (m, 3H), 5.14 (s, 2H), 3.47 (s, 3H), 2.97 (dd, J = 8.9, 6.3 Hz, 2H), 2.90 – 2.83 (m, 2H); **^{13}C NMR** (125 MHz, $CDCl_3$) δ 176.8, 167.9, 155.1, 139.5, 131.6 (q, 2C, J = 33.5 Hz), 129.7 (t, 2C, J = 3.9 Hz), 129.2, 127.1, 125.3 – 125.0 (m), 123.4 (q, 2C, J = 272.8 Hz), 118.7, 116.5, 115.7, 94.7, 56.1, 30.3, 24.3; **IR** (cm^{-1}) 1598, 1563, 1343, 1268, 1236, 1122, 1025, 910, 820, 798, 701, 681; **HRMS** (EI): Calculated for $C_{20}H_{16}F_6N_2O_3$ $[M]^+$: 446.1065, Found: 446.1068.

(*Z*)-*N*-(1,4-Dihydrobenzo[f]quinolin-3(2*H*)-ylidene)-3,5-bis(trifluoromethyl)benzamide (Scheme 5,

7o)

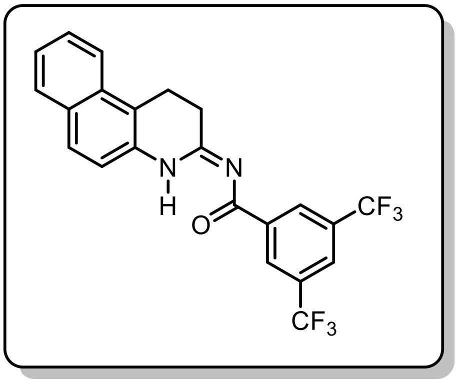

Compound **7o** was prepared from **5o** and **3i** according to the above general procedure with 16 h for step 1 and 27 h for step 2; eluent: ethyl acetate:hexane = 1:9; yield: 36.7 mg (17%); White solid; **^{1}H NMR** (500 MHz, $CDCl_3$) δ 13.10 (s, 1H), 8.78 – 8.74 (m, 2H), 7.99 (s, 1H), 7.95 (dd, J = 8.5, 1.1 Hz, 1H), 7.83 (dd, J = 8.1, 1.3 Hz, 1H), 7.78 (d, J = 8.6 Hz, 1H), 7.58 (ddd, J = 8.4, 6.8, 1.3 Hz, 1H), 7.47 (ddd, J = 8.1, 6.8, 1.1 Hz, 1H), 7.14 (d, J = 8.6 Hz, 1H), 3.39 (dd, J = 8.9, 7.2 Hz, 2H), 3.05 (dd, J = 8.8, 7.2 Hz, 2H); **^{13}C NMR** (125 MHz, $CDCl_3$) δ 175.0, 167.9, 139.3, 131.8, 131.6, 131.6 (q, J = 33.6 Hz, 2C), 131.2, 129.7 (d, J = 4.1 Hz, 2C), 128.8, 128.6, 127.3, 125.4, 125.3 – 125.1 (m), 123.3 (q, J = 272.8 Hz, 2C), 122.8, 119.2, 117.6, 30.0, 19.8; **IR** (cm^{-1}) 1581, 1338, 1269, 1250, 1158, 1127, 937, 903, 807, 780, 743, 682; **HRMS** (EI): Calculated for $C_{22}H_{14}F_6N_2O$ $[M]^+$: 436.1010, Found: 436.1012.

(*Z*)-*N*-(6-Phenyl-3,4-dihydroquinolin-2(1*H*)-ylidene)-3,5-bis(trifluoromethyl)benzamide (Scheme 5, 7p)

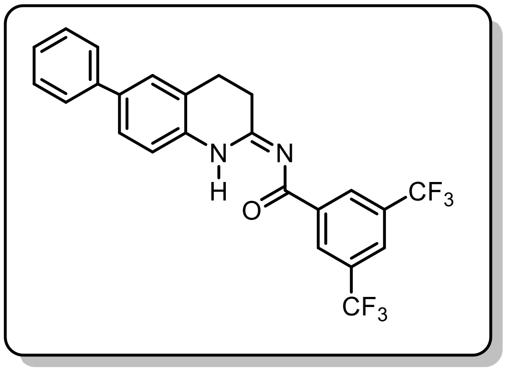

Compound **7p** was prepared from **5p** and **3i** according to the above general procedure with 14 h for step 1 and 48 h for step 2; eluent: ethyl acetate:hexane = 1:9; yield: 110.7 mg (48%); Lemon yellow solid; **^{1}H NMR** (500 MHz, $CDCl_3$) δ 13.10 (s, 1H), 8.78 – 8.74 (m, 2H), 8.01 (d, J = 2.0 Hz, 1H), 7.61 – 7.54 (m, 2H), 7.48 (dd, J = 8.1, 2.1 Hz, 1H), 7.47 – 7.43 (m, 3H), 7.40 – 7.33 (m, 1H), 7.04 (d, J = 8.1 Hz, 1H), 3.08 (dd, J = 8.8, 6.5 Hz, 2H), 2.98 – 2.92 (m, 2H); **^{13}C NMR** (125 MHz, $CDCl_3$) δ 176.9, 168.2, 140.0, 139.3 138.7, 133.9, 131.6 (q, J = 33.7 Hz), 129.7 (d, J = 4.2 Hz, 2C), 128.9 (s, 2C), 127.5, 127.1, 126.8, 126.6 (s, 2C), 125.9, 125.4 – 125.0 (m), 122.2 (q, J = 274.1 Hz,2C), 117.9, 30.4, 24.1; **IR** (cm^{-1}) 1557, 1563, 1276, 1248, 1122, 908, 816, 758, 681; **HRMS** (EI): Calculated for $C_{24}H_{16}F_6N_2O$ $[M]^+$: 462.1167, Found: 462.1171.

VII. The relative rates of hydrosilylation and [3 + 2] cycloaddition (Scheme 6)

1. Hydrosilylation of quinolines (5a, 5m, 5q)

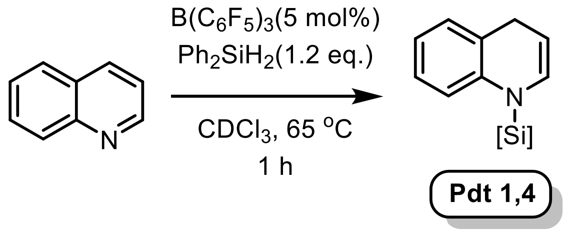

1) NMR data of final reaction mixtrues (1~16 h) and structural assignments.

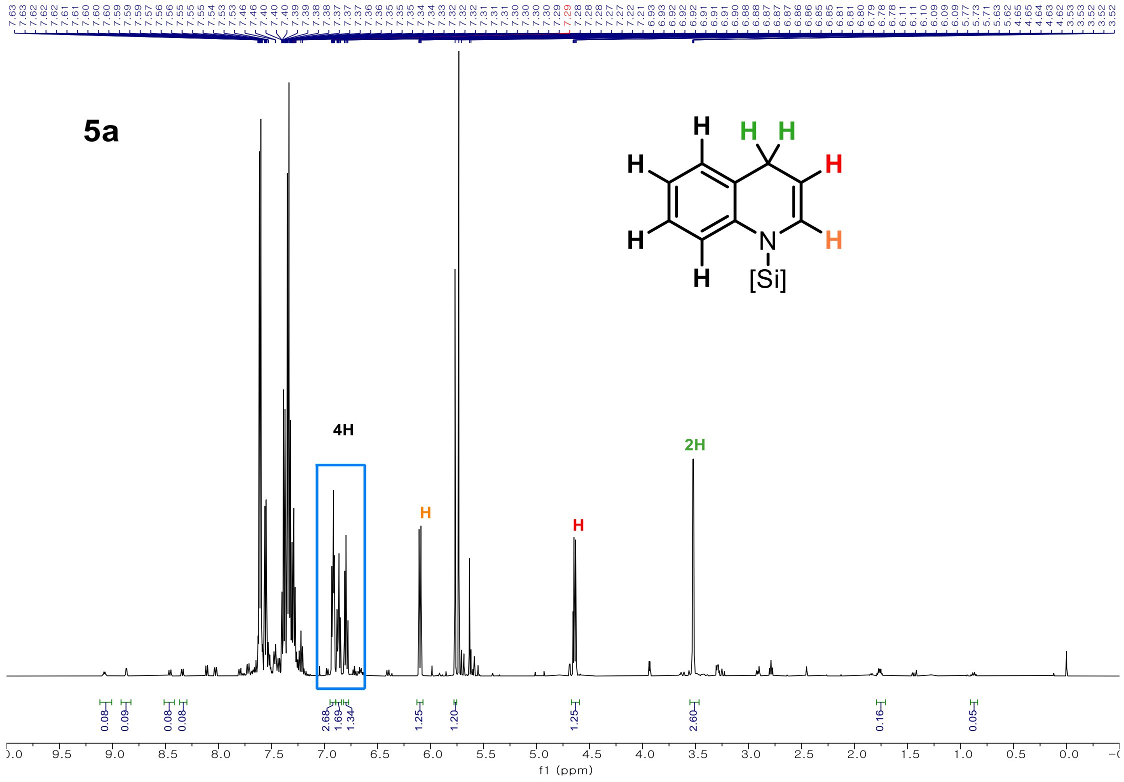

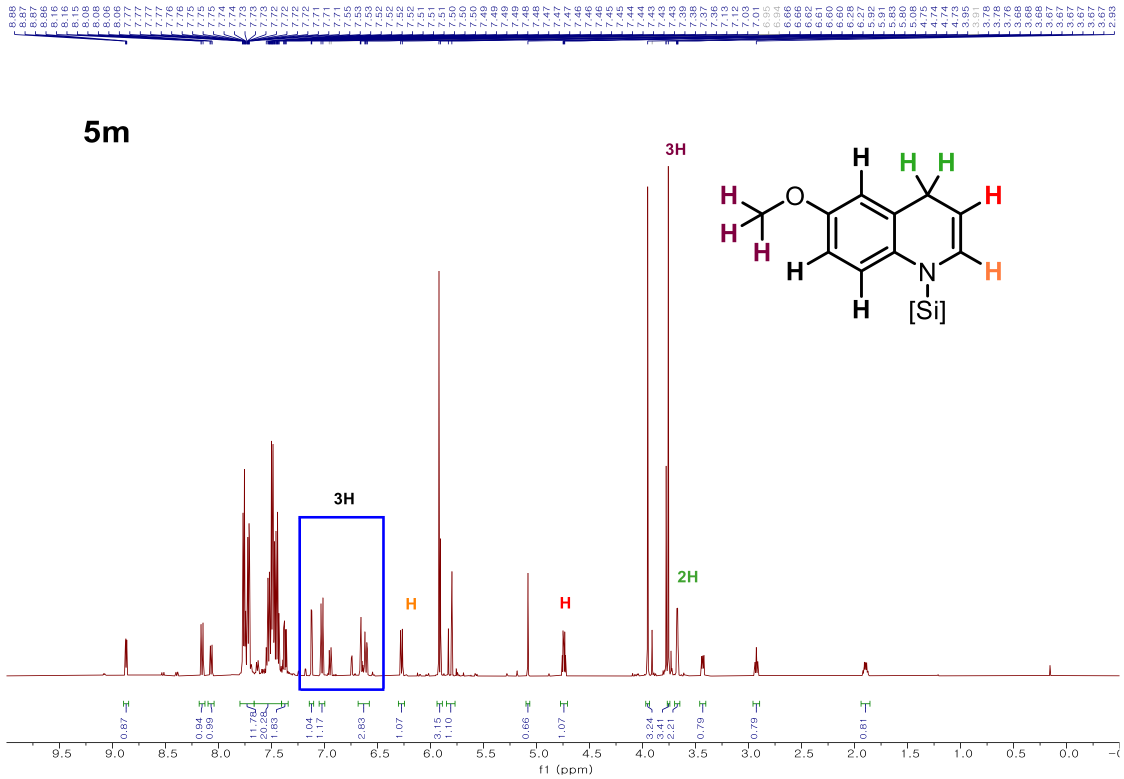
5m
3H
3H
2H
H
H
[Si]
N
O
f1 (ppm)

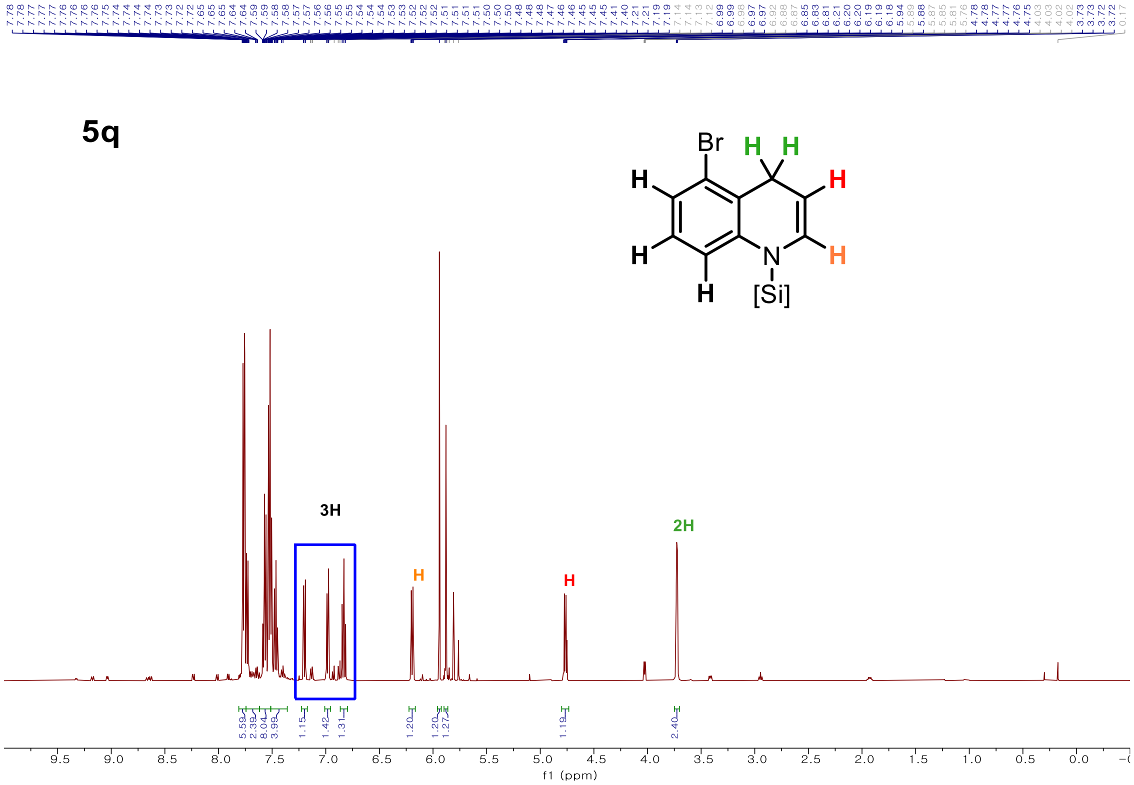
5q
Br
3H
2H
H
H
N
[Si]
f1 (ppm)

2) Reaction monitoring NMR data stack

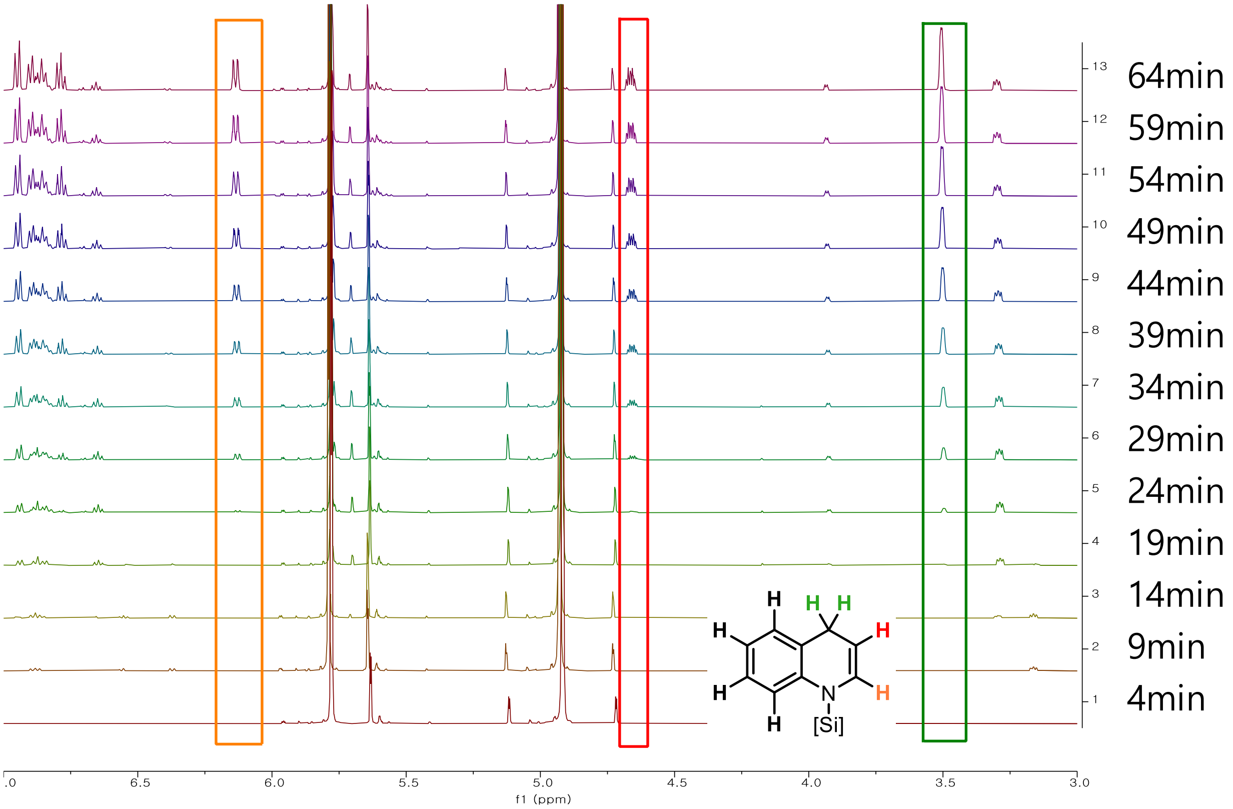

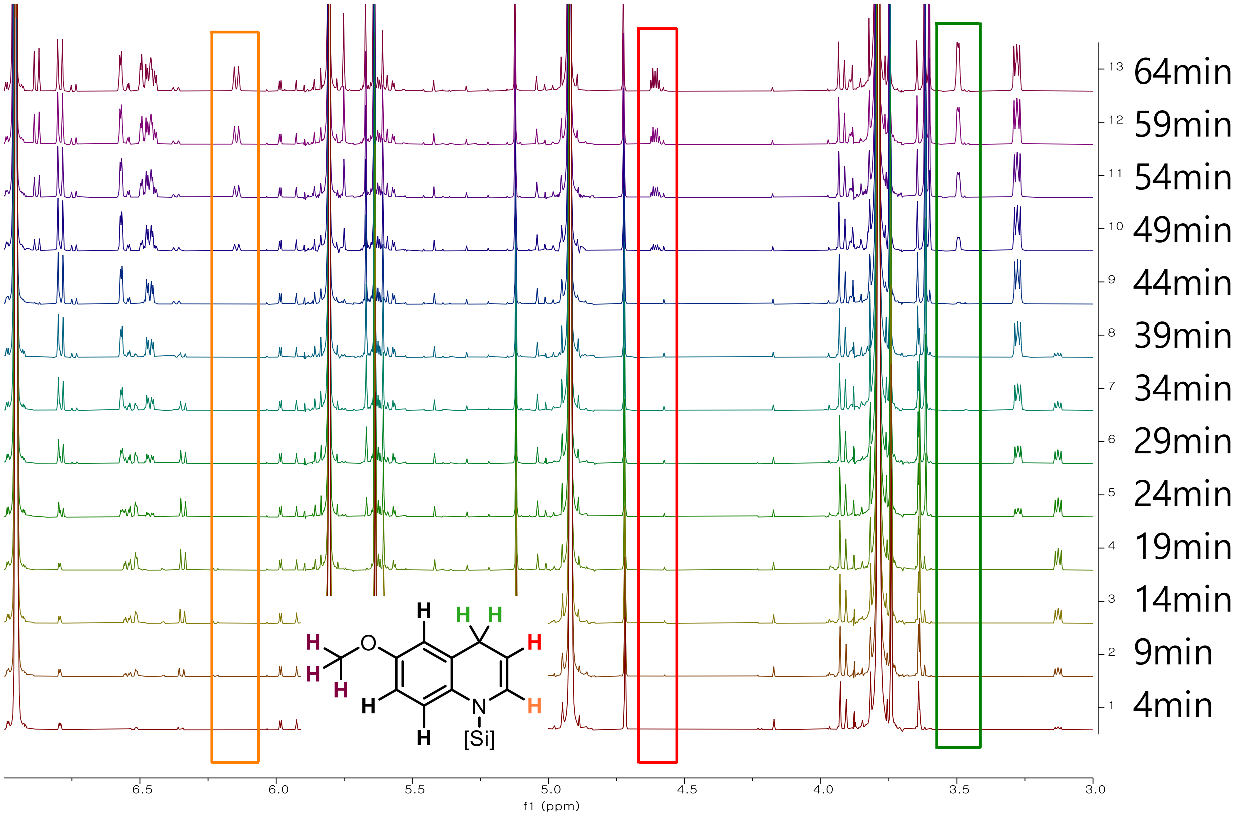

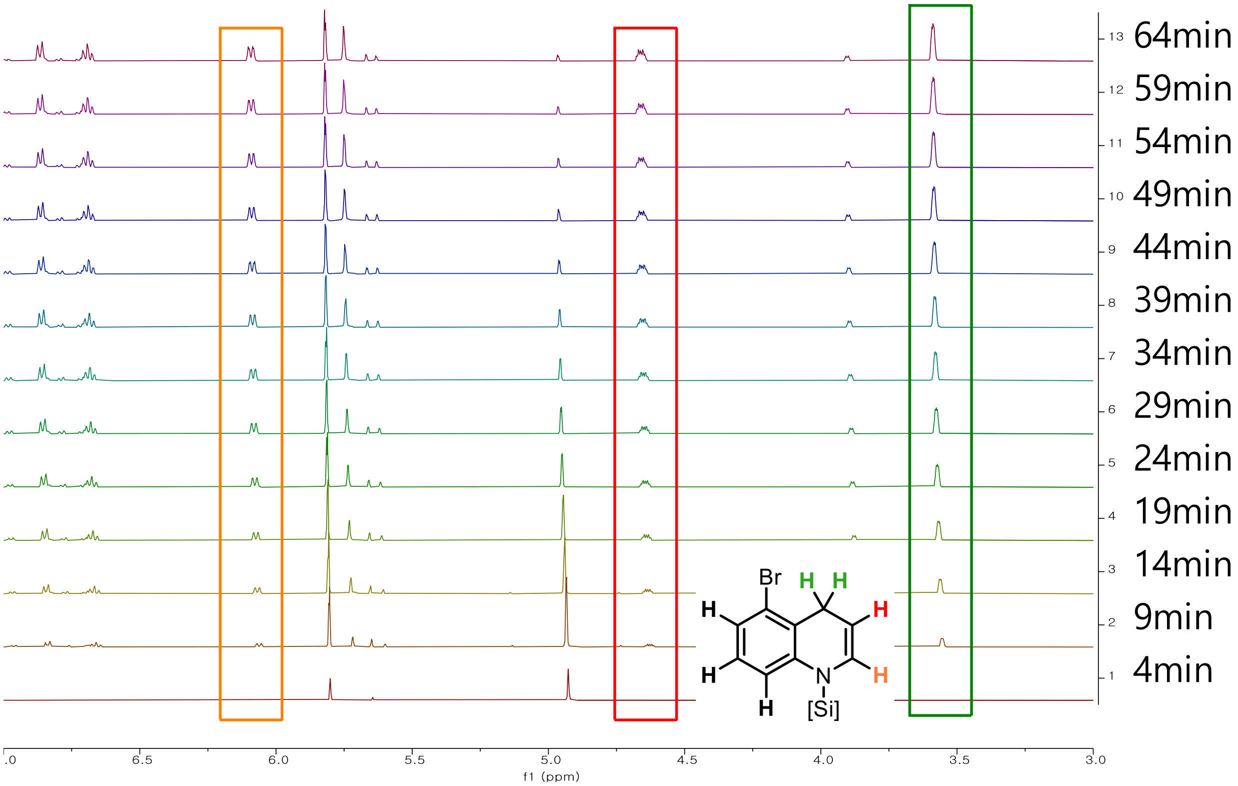

64min
59min
54min
49min
44min
39min
34min
29min
24min
19min
14min
9min
4min
Br
H
H
H
H
H
H
H
N
[Si]
f1 (ppm)

3) NMR data of 64 min and the peak of interest for calculation.

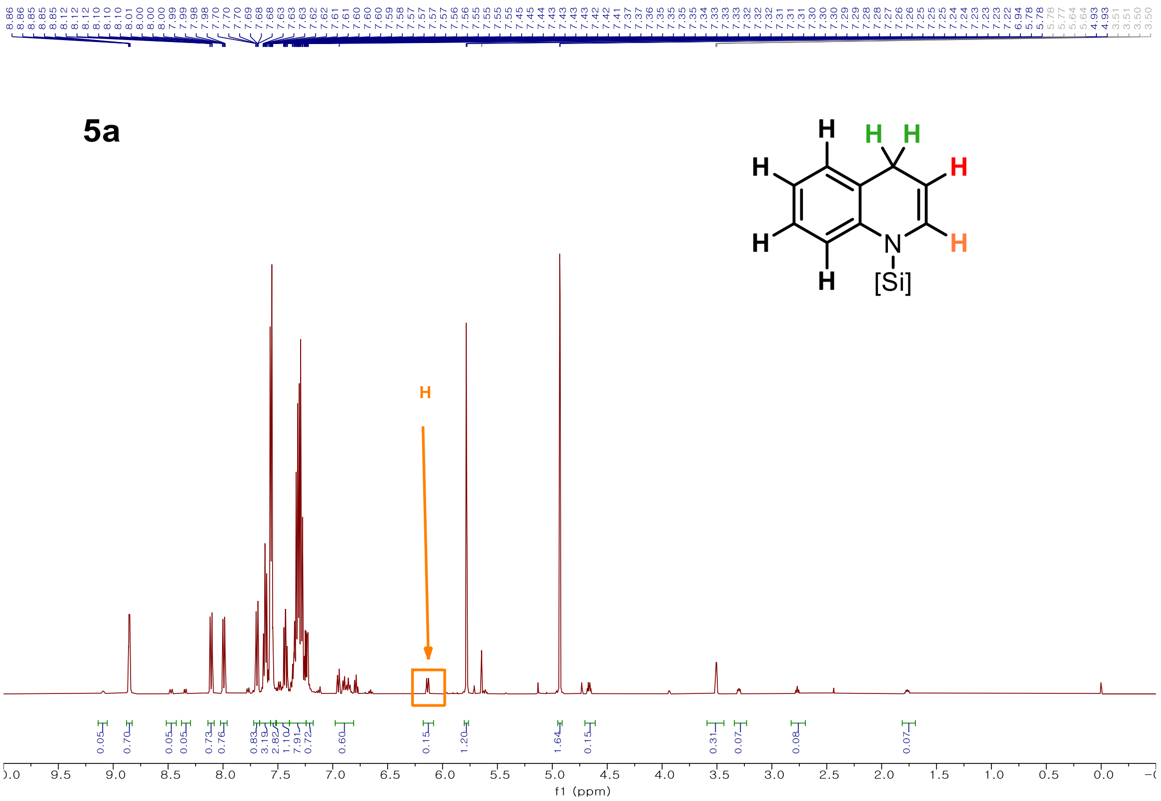

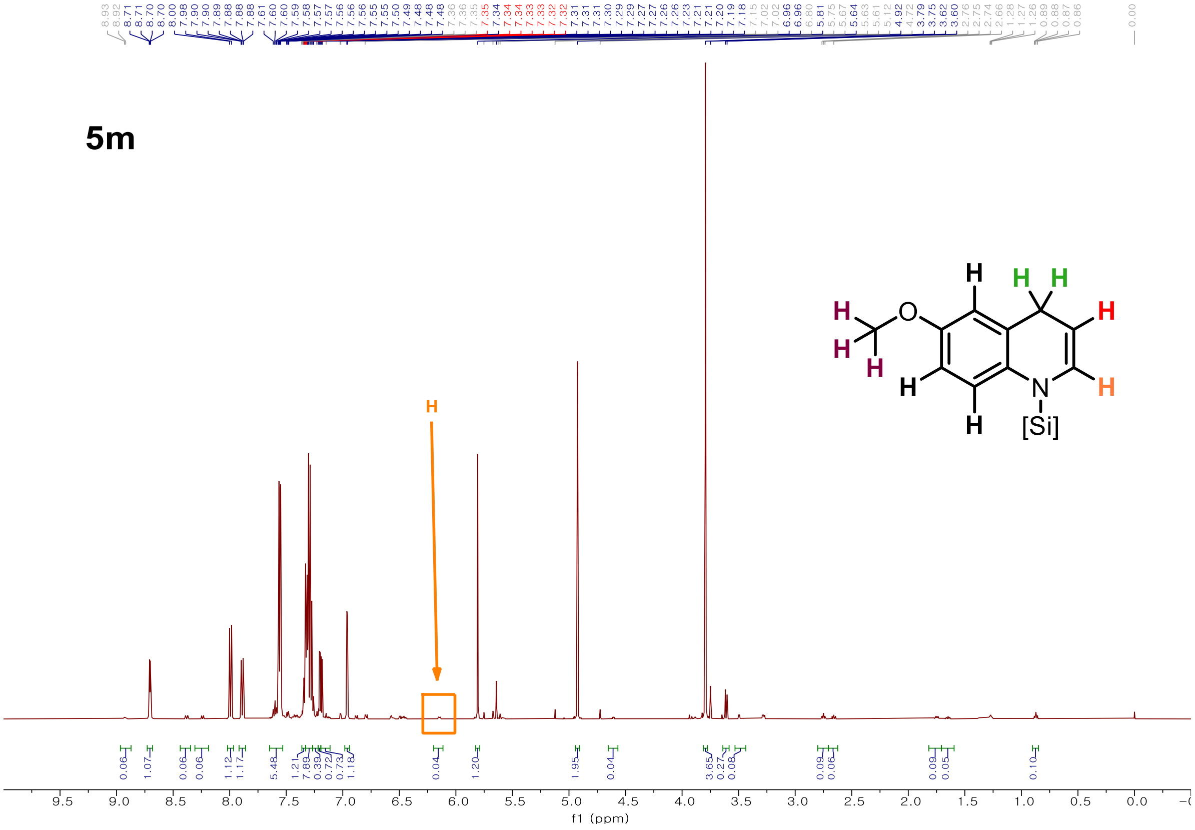

5q

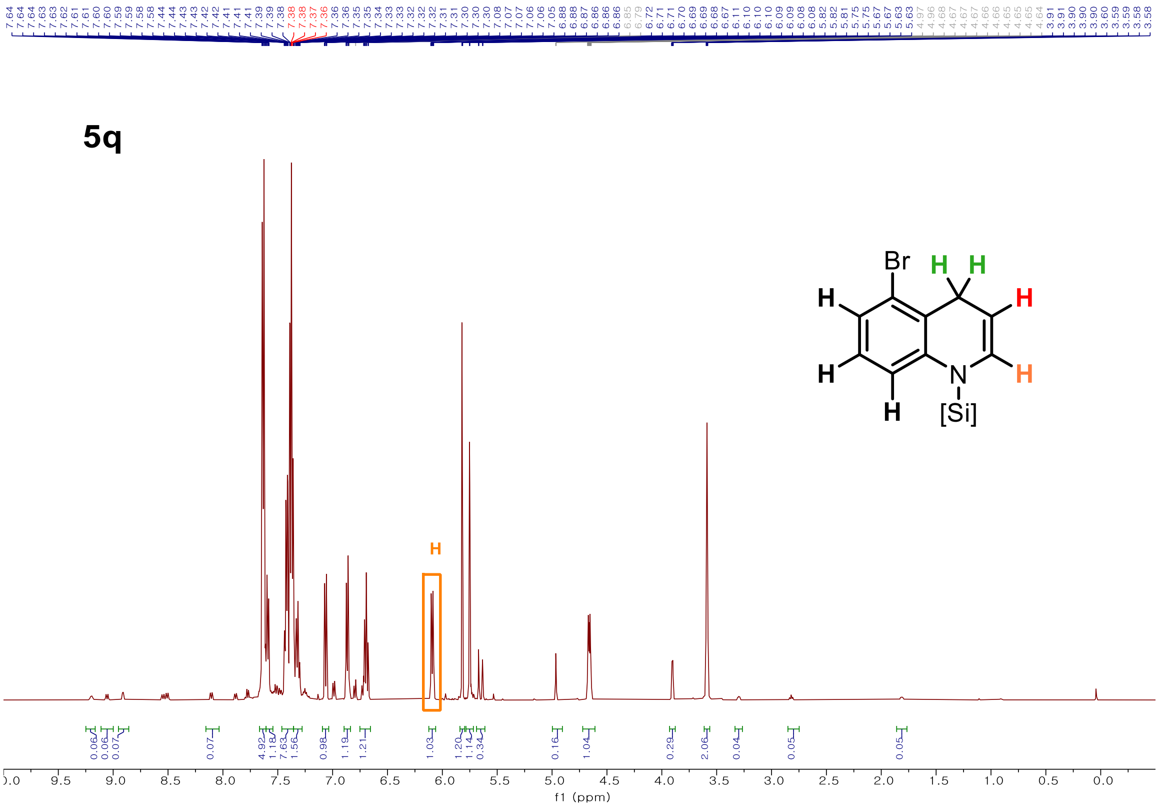

4) Table and chart of the reaction monitoring.

time (min)	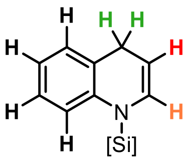	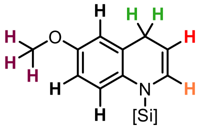	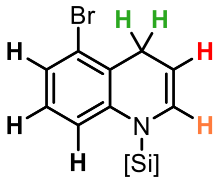
0 min	0%	0%	0%
4 min	0%	0%	7%
9 min	0%	0%	23%
14 min	0%	0%	35%
19 min	0%	0%	52%
24 min	1%	0%	62%
29 min	3%	0%	71%
34 min	5%	0%	78%
39 min	7%	0%	83%
44 min	8%	0%	88%
49 min	10%	0%	93%
54 min	12%	0%	96%
59 min	13%	3%	100%
64 min	15%	4%	103%

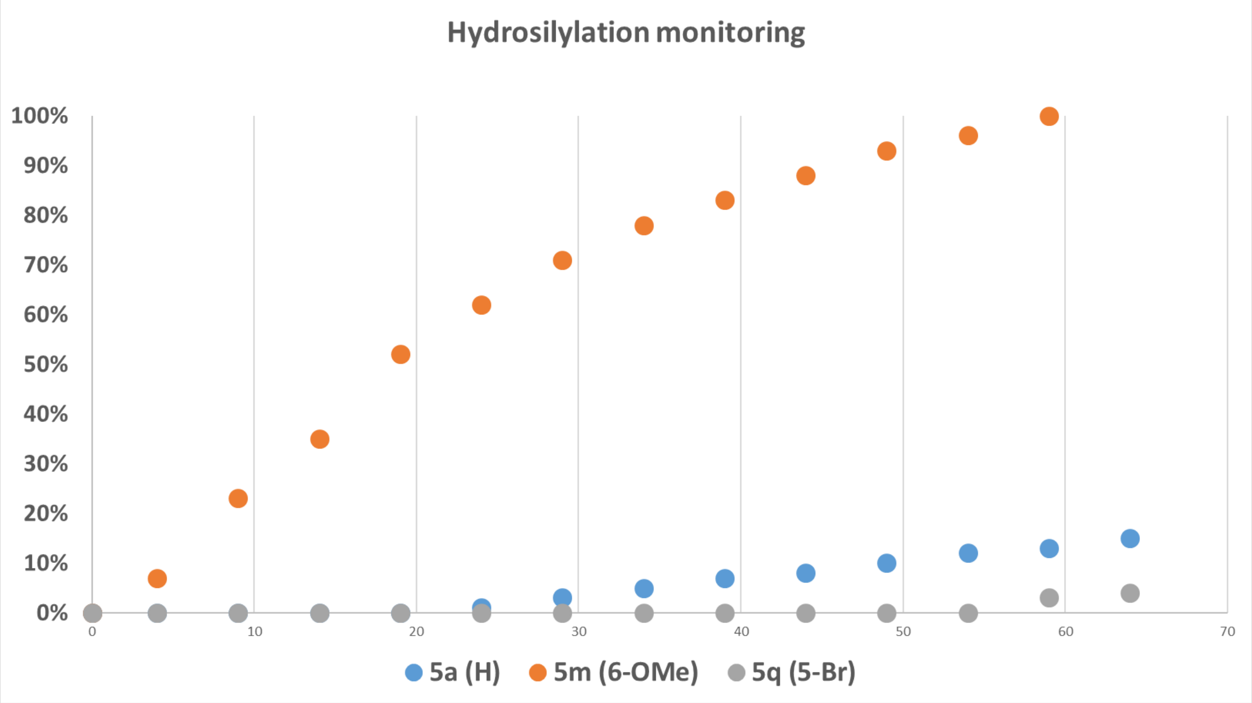

2. [3+2] cycloaddition of *N*-silyl enamines (6a', 6m', 6q') and 3i [S3]

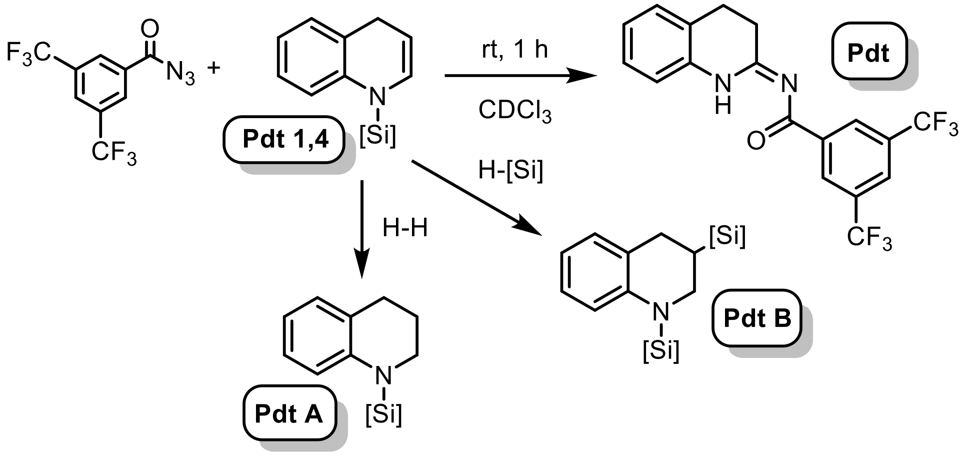

1) NMR data of 1st step for 6a', 6m', and 6q' to assign Pdt 1,4, Pdt B, and Pdt A

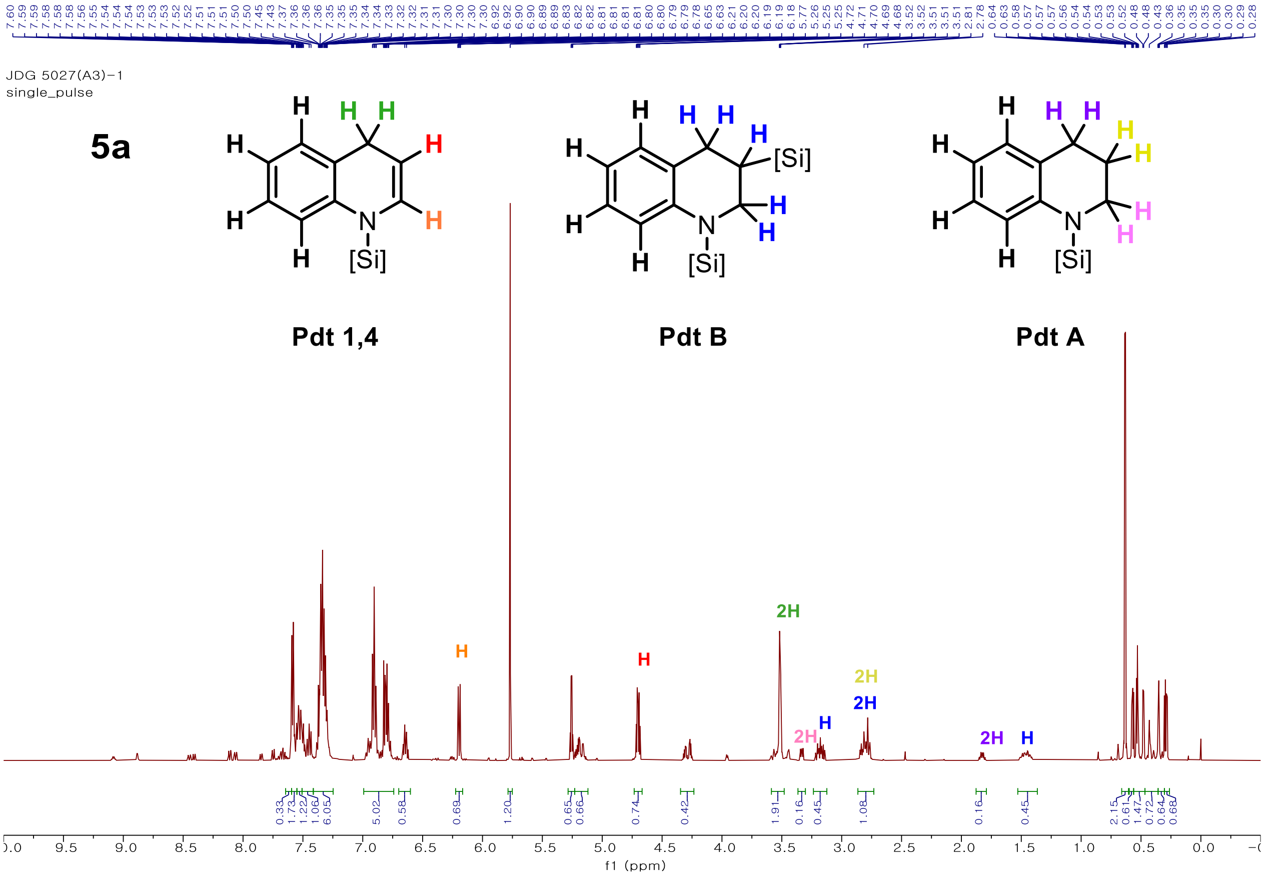

5m

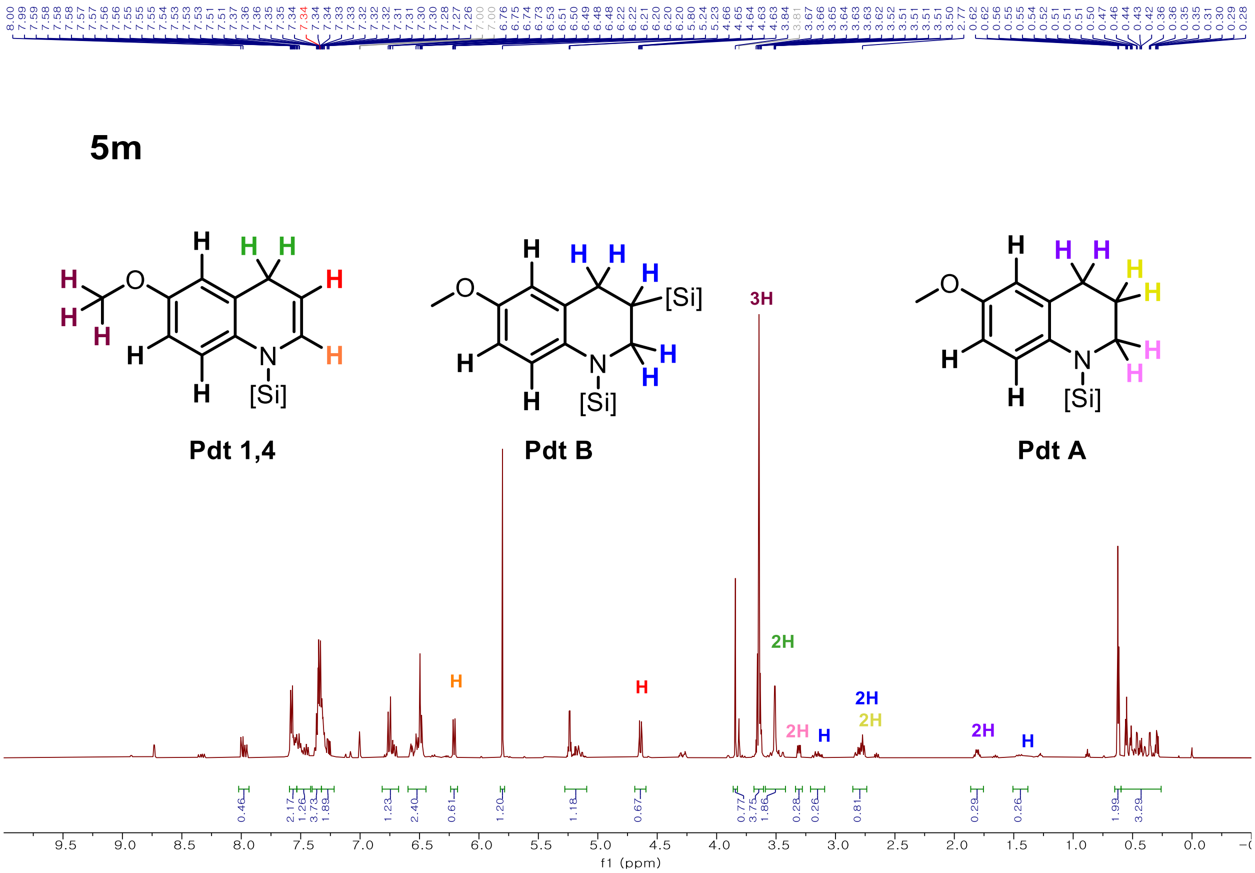

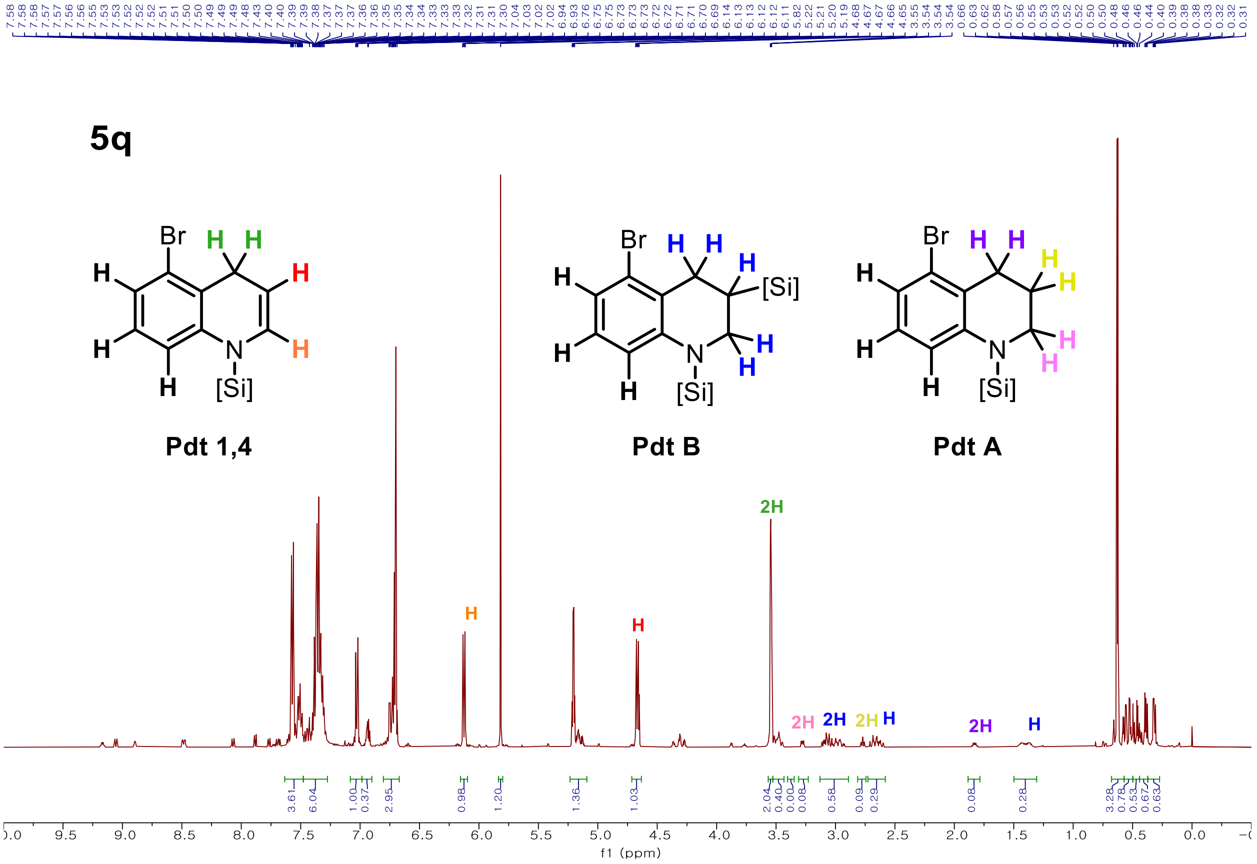
5q
Pdt 1,4
Pdt B
Pdt A
f1 (ppm)

2) NMR data of final reaction mixtrues (16~48 h) and structural assignments.

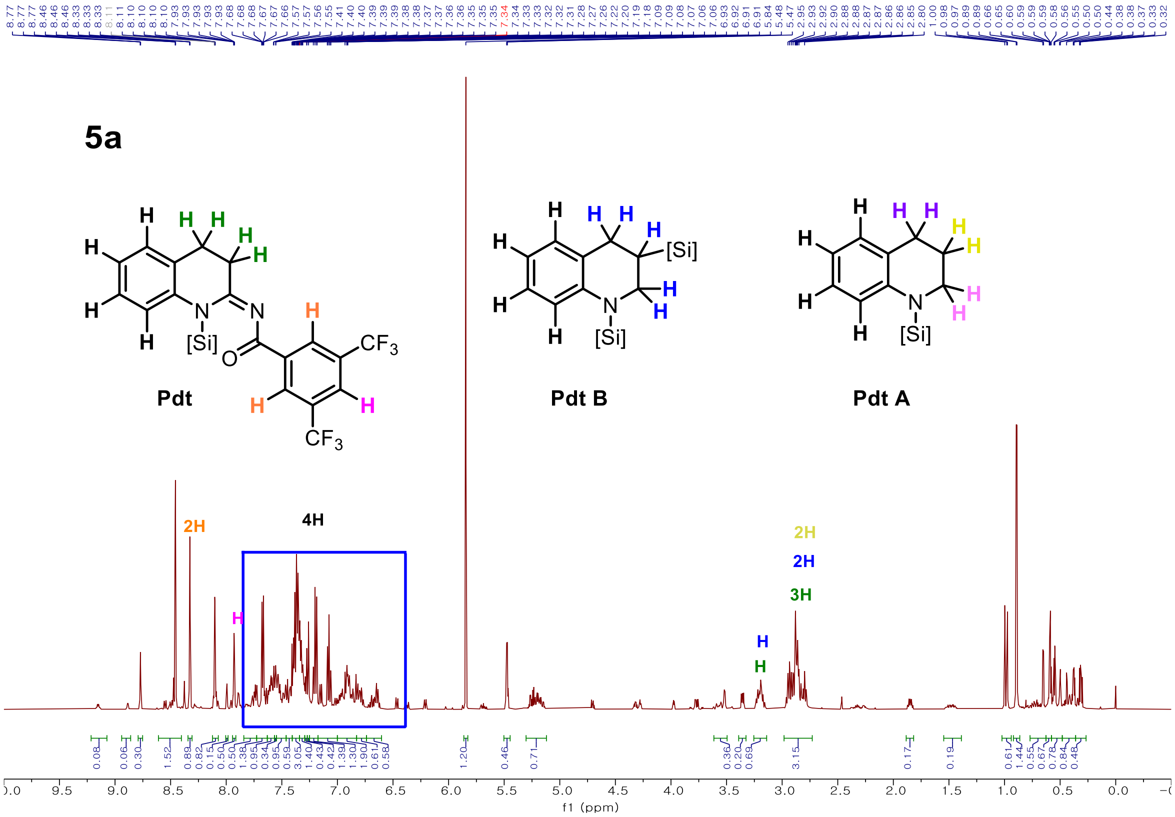

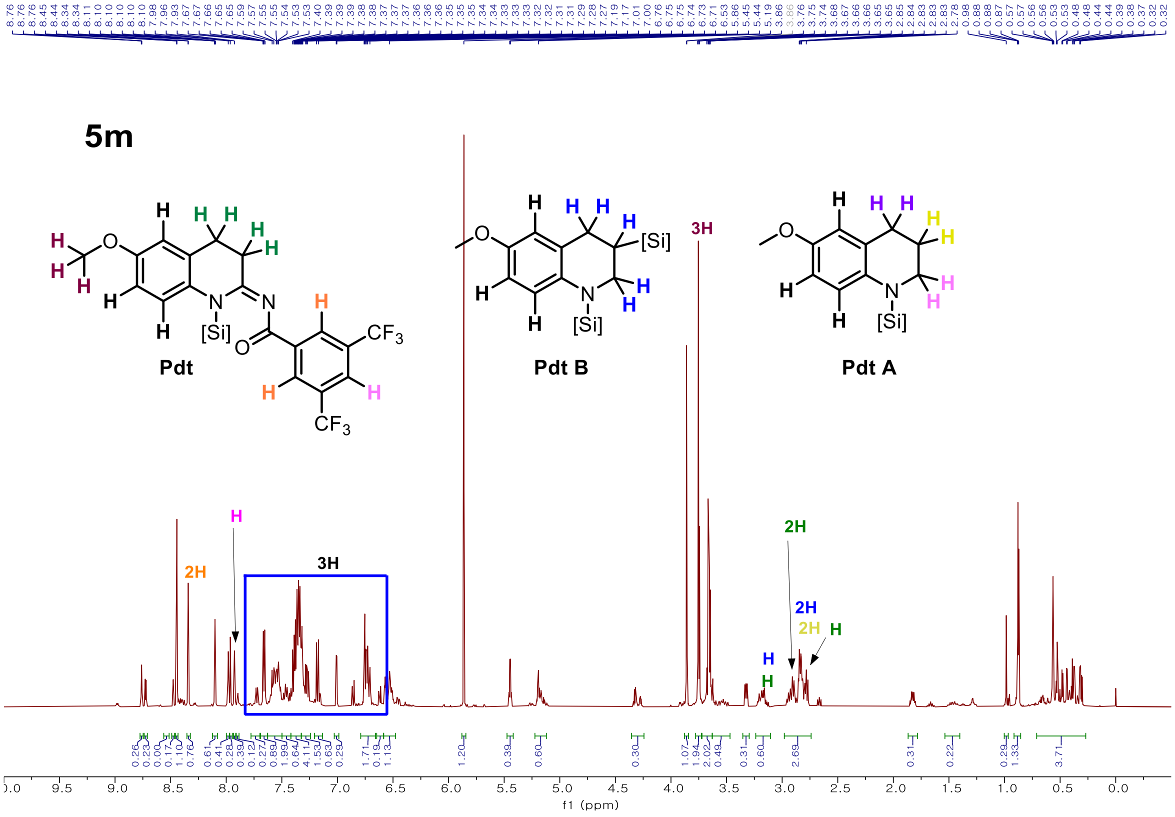

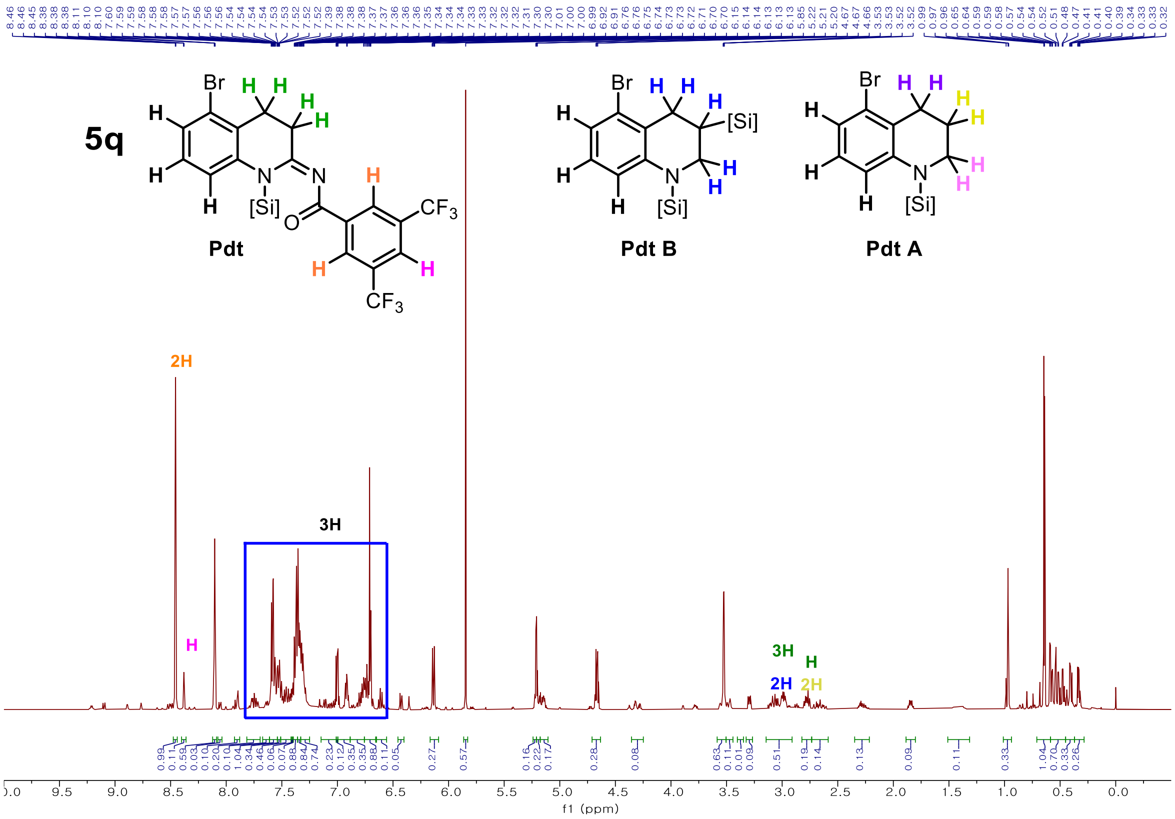
5q
Pdt
Pdt B
Pdt A
2H
H
3H
3H
2H
H
2H
f1 (ppm)

3) Reaction monitoring NMR data stack

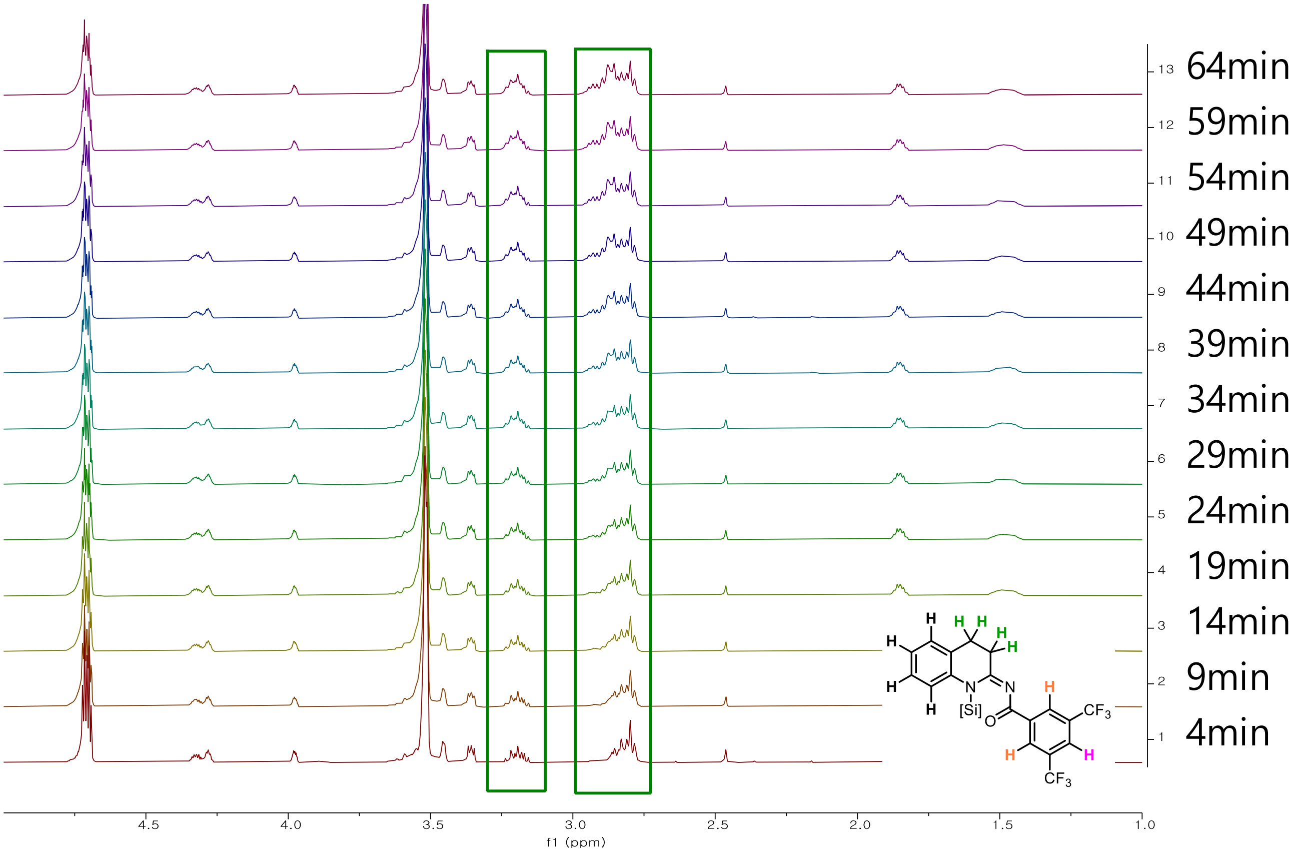

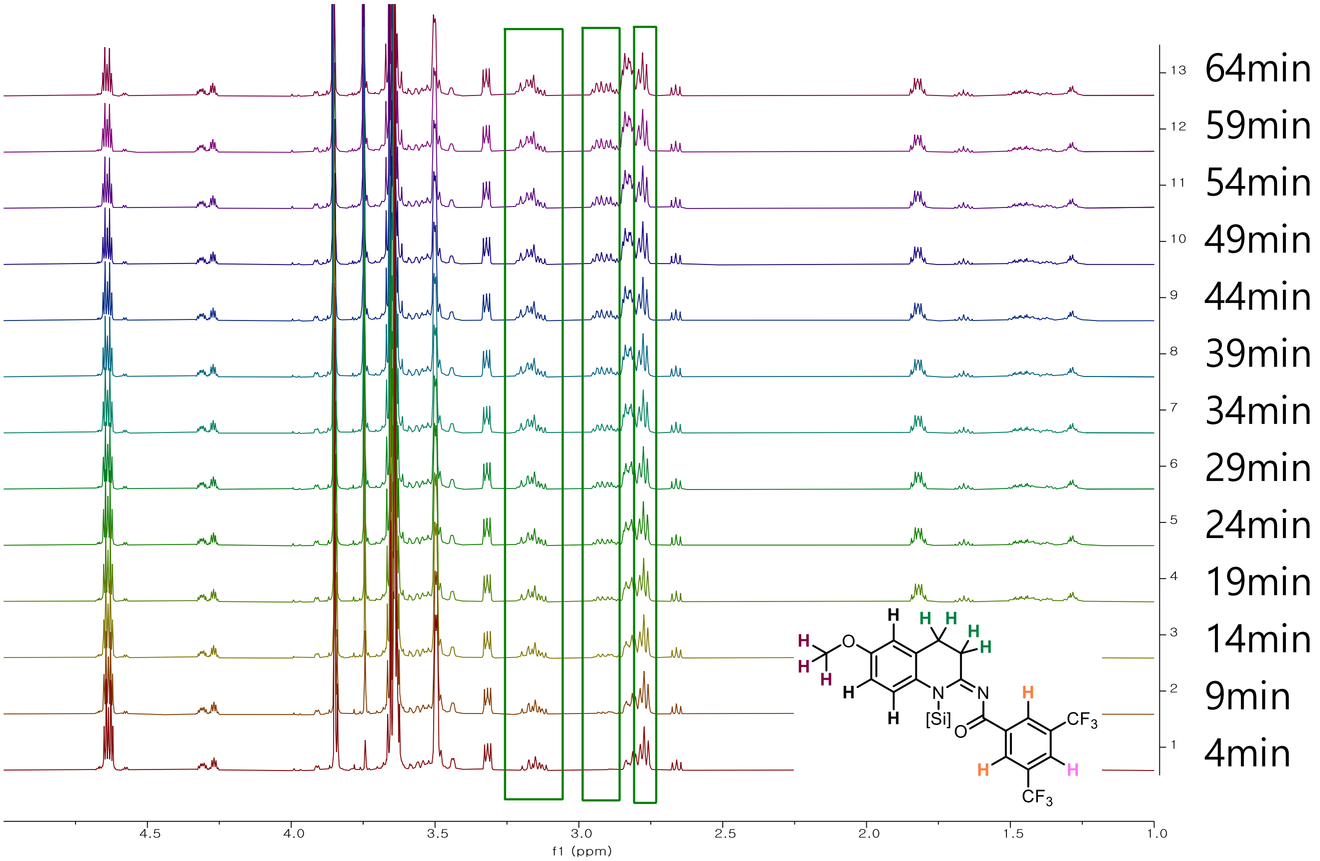
64min
59min
54min
49min
44min
39min
34min
29min
24min
19min
14min
9min
4min
[Si]
CF3
f1 (ppm)

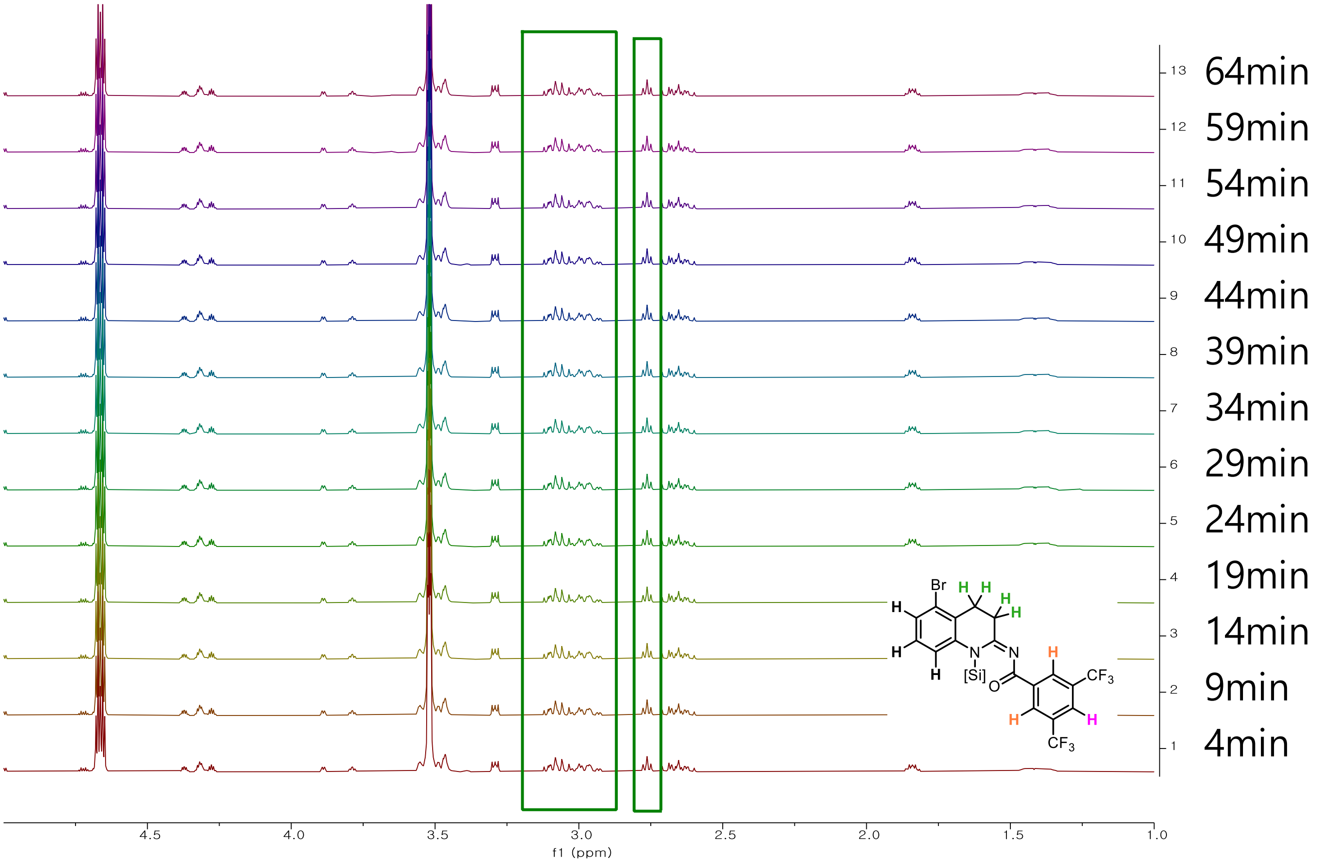
64min
59min
54min
49min
44min
39min
34min
29min
24min
19min
14min
9min
4min
Br
[Si]
CF3
f1 (ppm)

4) NMR data of 64 min and the peak of interest for calculation

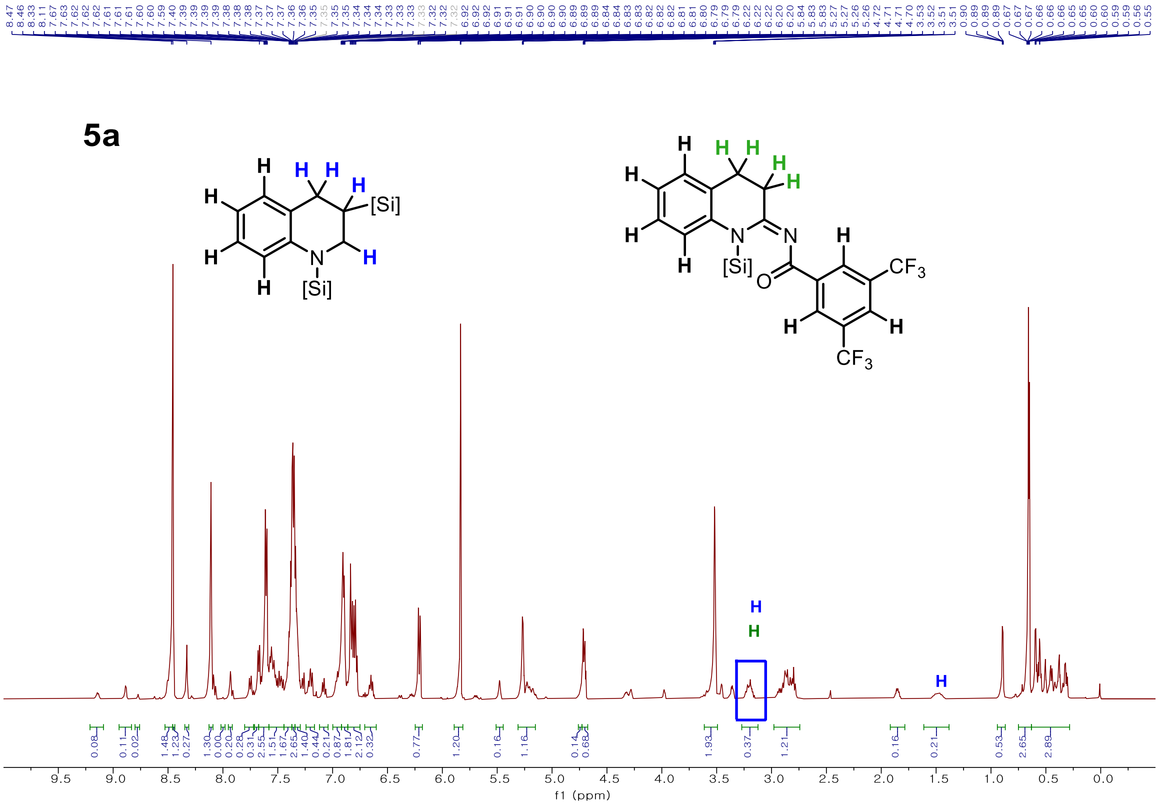

Integration 0.37 (H+H) – 0.21 (H) = 0.16 (H)

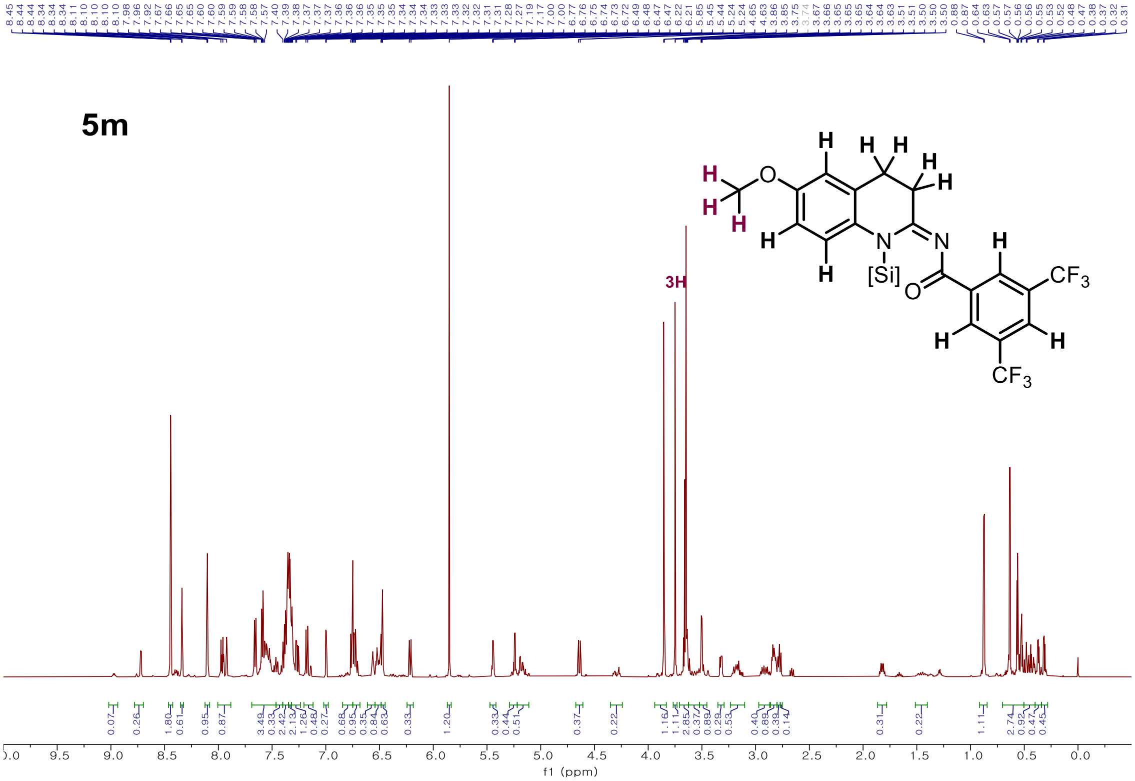

Integration 1.11 (3H) / 3 = 0.37 (H)

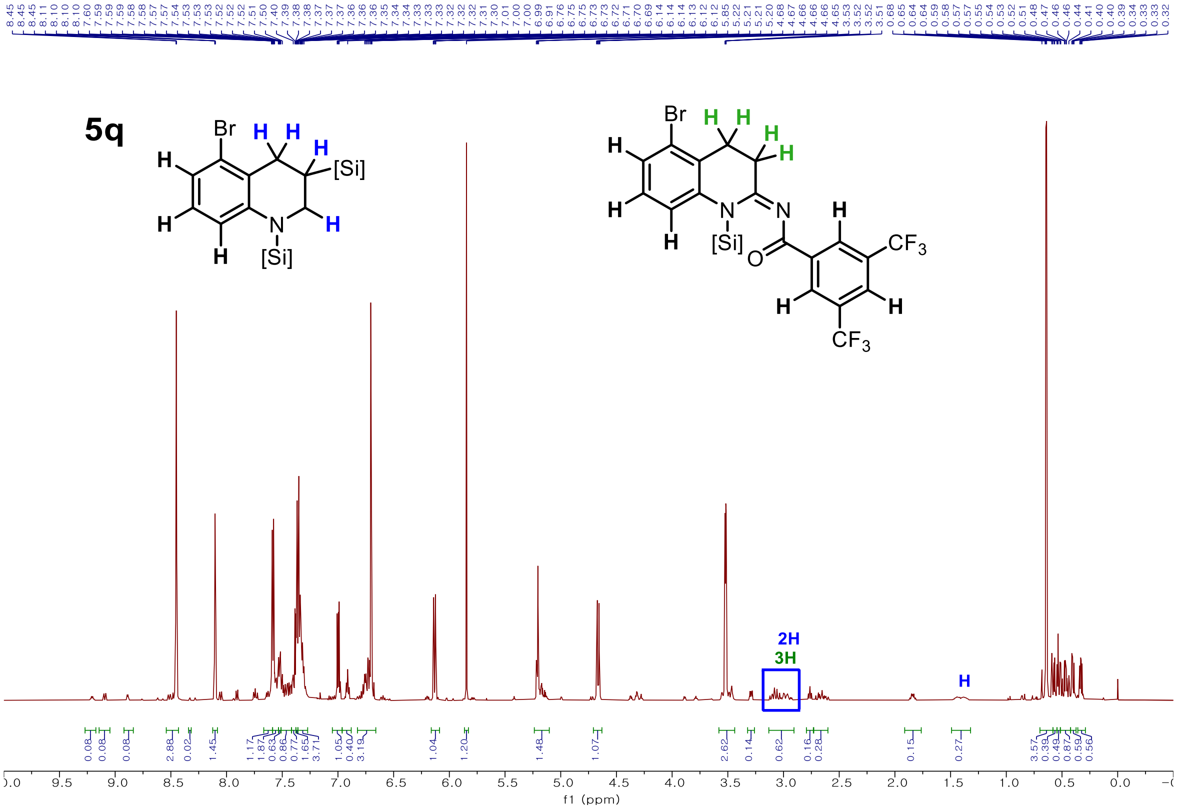

Integration (0.62 (2H + 3H) – 2*(0.27 (H))) / 3 = 0.026 (H)

5) Table and chart of the reaction monitoring.

time (min)	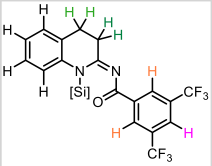	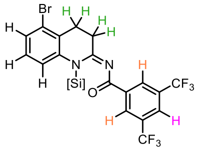	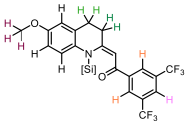
0 min	0%	0%	0%
4 min	0%	1%	3%
9 min	1%	0%	7%
14 min	0%	1%	12%
19 min	5%	2%	15%
24 min	7%	1%	19%
29 min	6%	1%	21%
34 min	6%	1%	24%
39 min	9%	2%	27%
44 min	8%	2%	29%
49 min	13%	2%	32%
54 min	13%	2%	34%
59 min	15%	2%	35%
64 min	16%	3%	37%

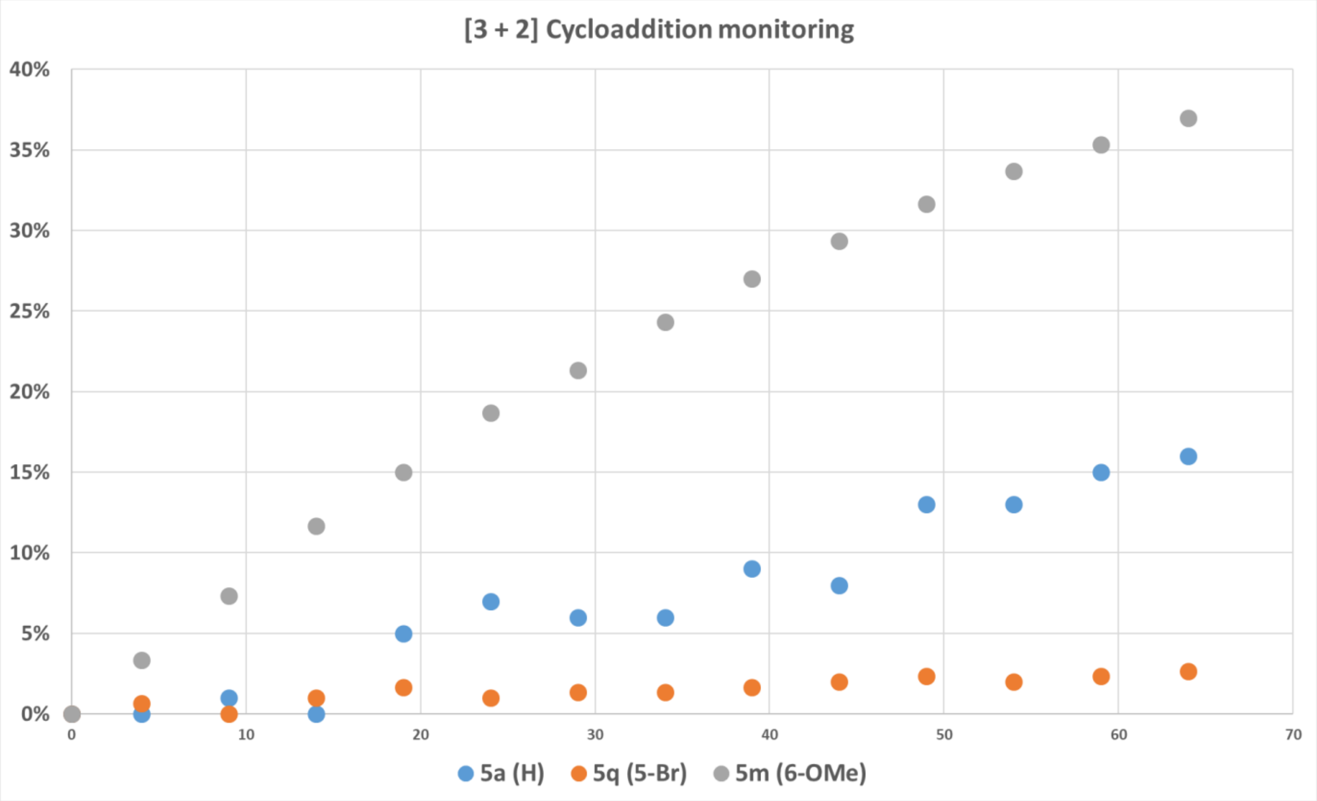

VIII. Gram scale reaction

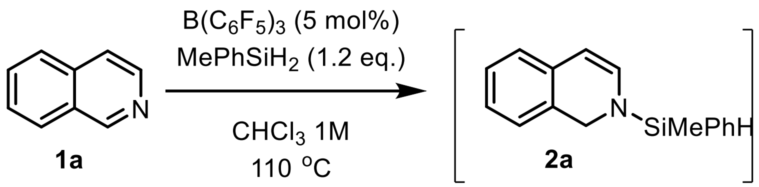

Step 1: To a $B(C_6F_5)_3$ catalyst (0.5mmol, 5 mol%) in 30 mL pressure vial sealed with rubber plugs was added $CHCl_3$ (10 mL) and $MePhSiH_2$ (12 mmol, 1.2 equiv) at room temperature under argon air. The reaction was stirred slowly to remove H_2 bubbles. Isoquinoline (10 mmol, 1.0 equiv.) was subsequently added to the mixture, closed by Teflon cap, stirred and heated up to 110 °C in oil bath for 32 h. The mixture was subjected to NMR to check conversion and yields of reactions.

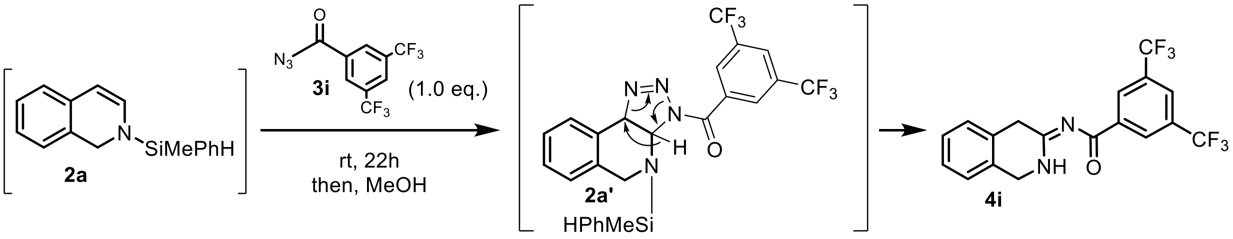

Step 2: The crude mixture solution of the first step was added acyl azide (10.0 mmol, 1.0 equiv.) at room temperature. The reaction mixture was stirred to react at room temperature, N_2 extrusion will be absorbed into the argon balloon. After 22 h, the mixture was subjected to NMR to check conversion and yields of reactions and quenched by MeOH addition, silica filter, and DCM wash. The crude mixtures were purified by column chromatography on silica gel. (EA/hexane/DCM = 0.5/9/0.5, 1.633 g, 42.4%)

IX. References

S1. S. Joung et al. *Org. Lett*. **2020**, *22*, 2, 515–519.

S2. V. D. Cao et al. *Synthesis* **2021**; *53*(4): 754-764.

S3. S. Chang et al. *J. Am. Chem. Soc.*, **2014**, *136*, 30, 10770–10776

S4. S. Chang et al. *J. Am. Chem. Soc.*, **2011**, *133*, 11, 3780–3783

S5. S. Chang et al. *Chem. Commun.*, **2017**, *53*, 8798

X. Copies of Spectroscopic Data

5-(Triisopropylsilyl)oxy quinoline (5k)

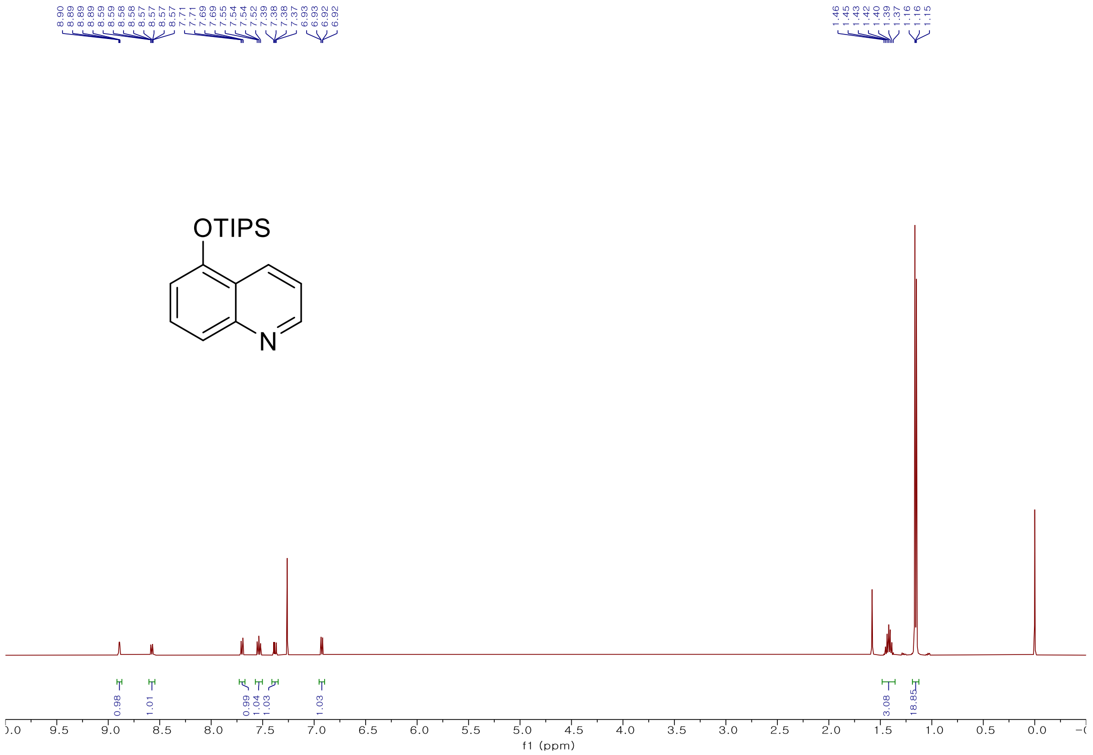

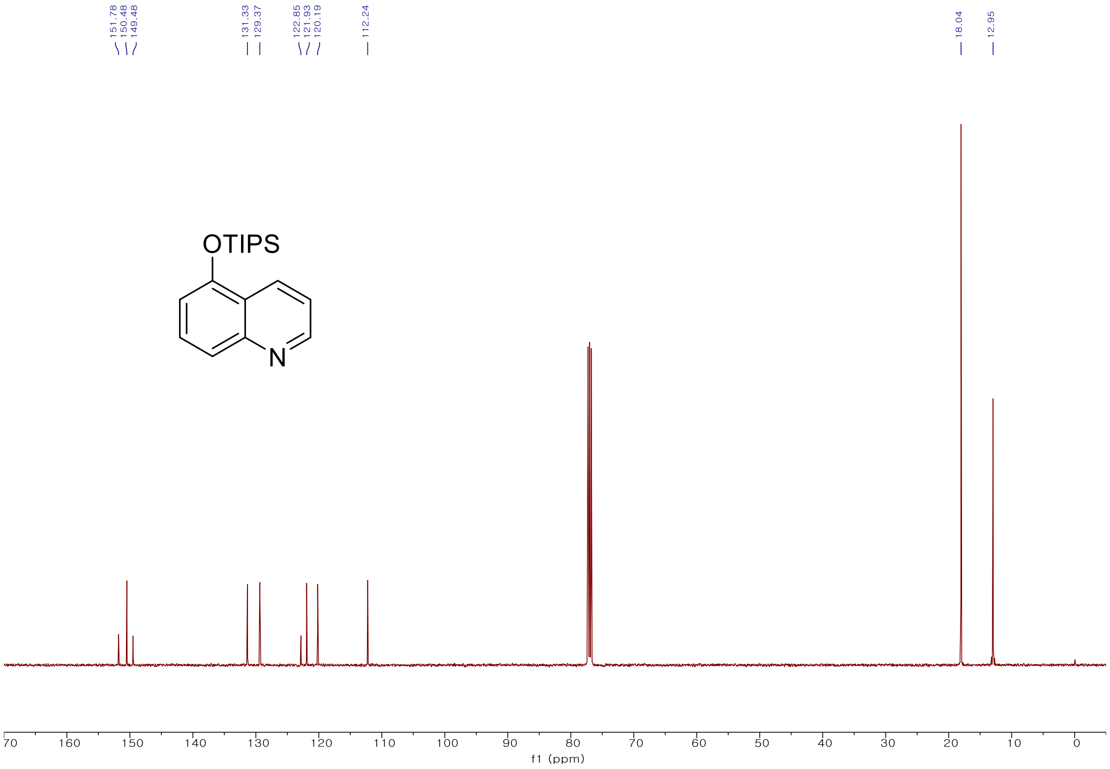

6-(Methoxymethoxy)quinoline (5n)

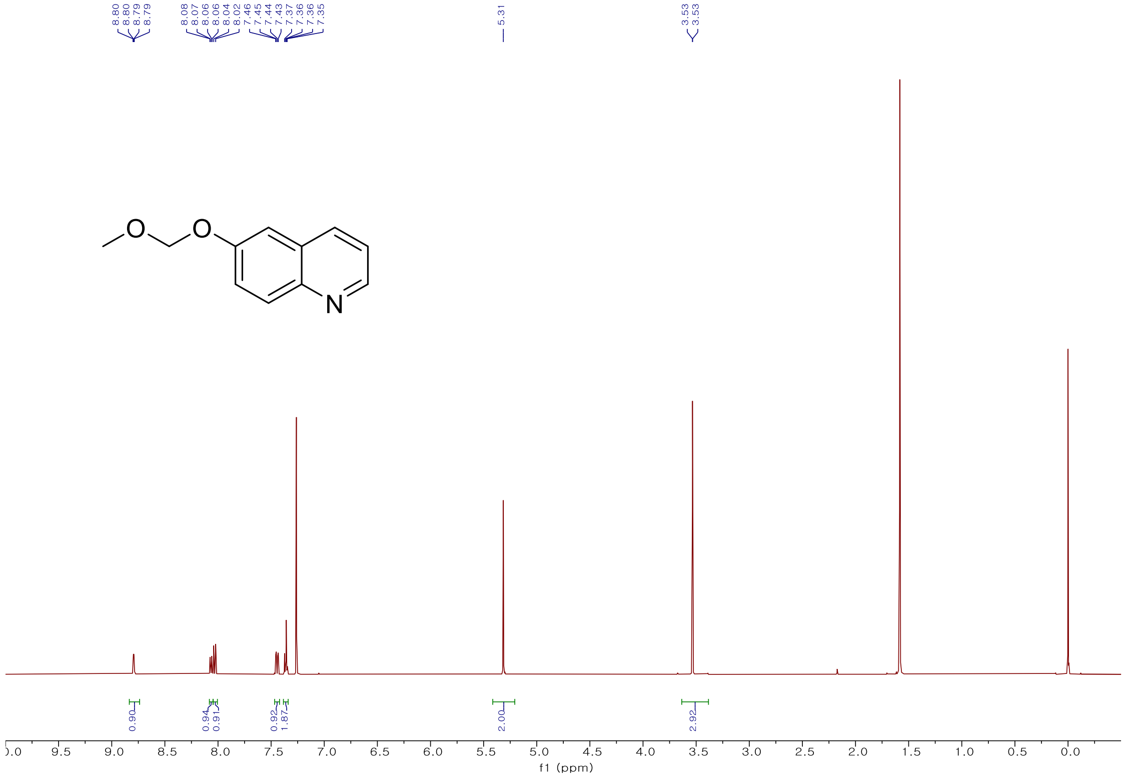

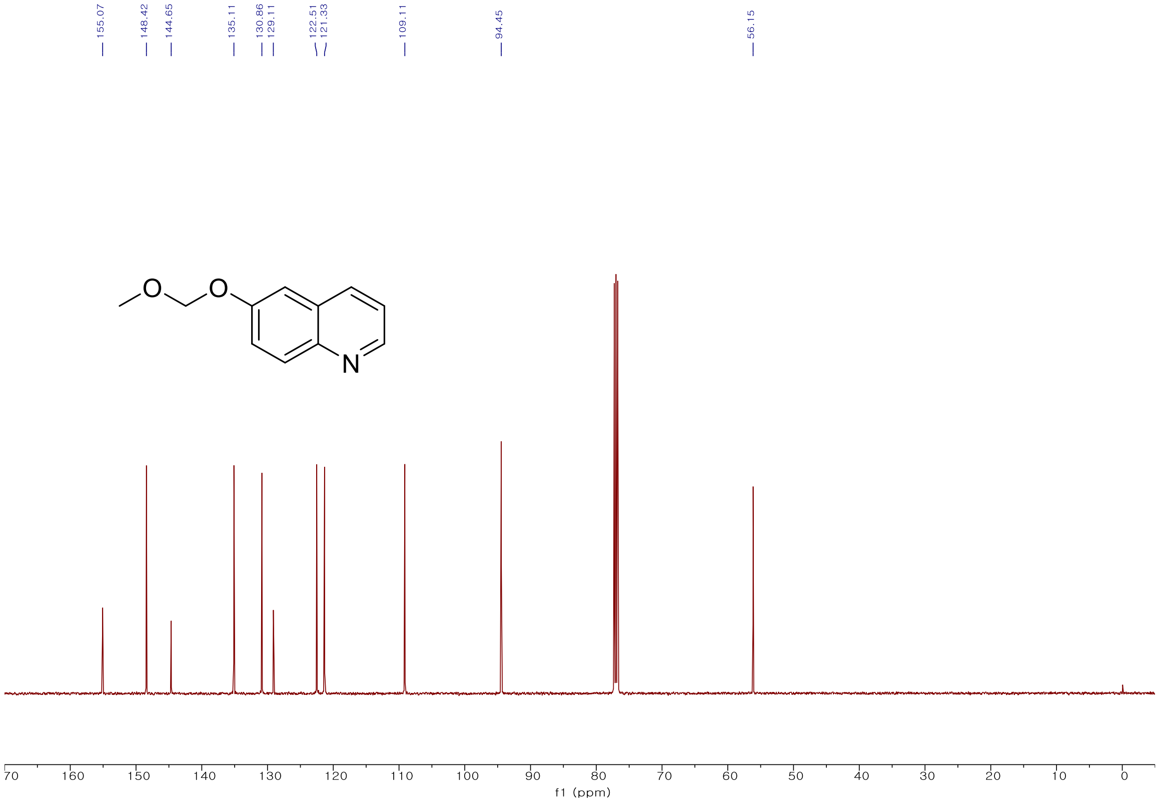

Benzoyl azide (3a)

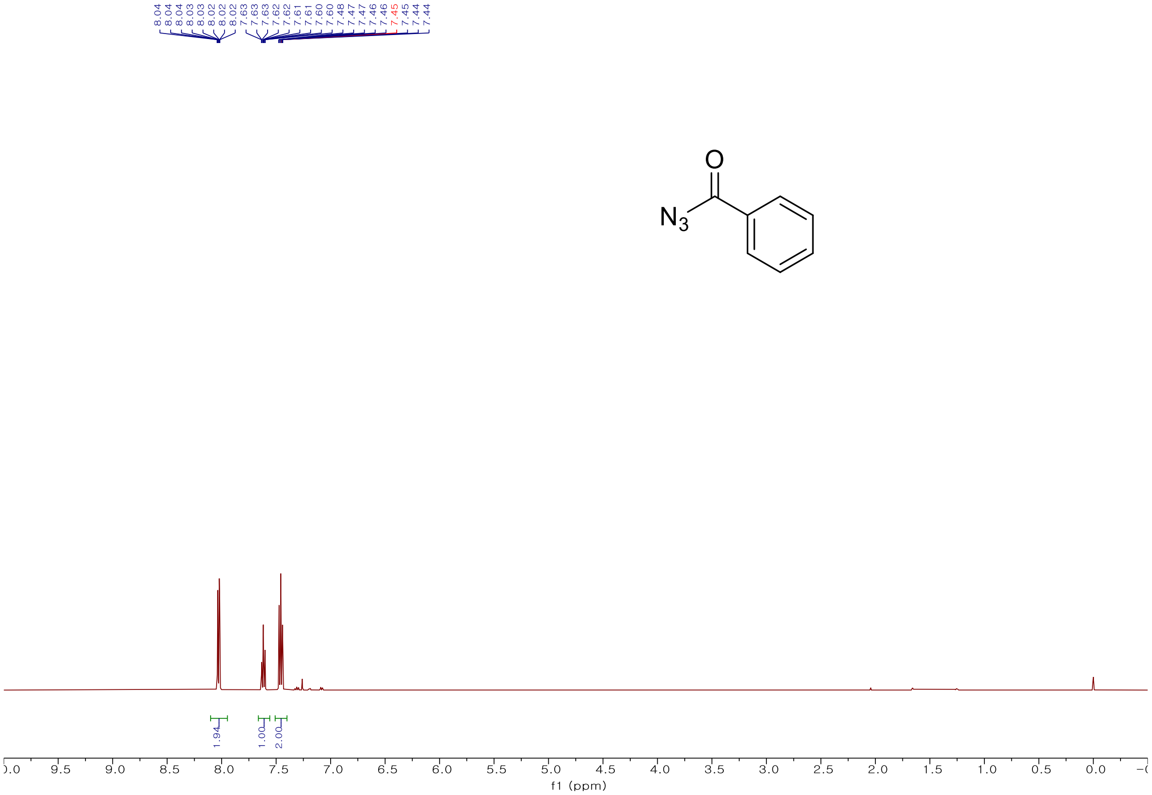

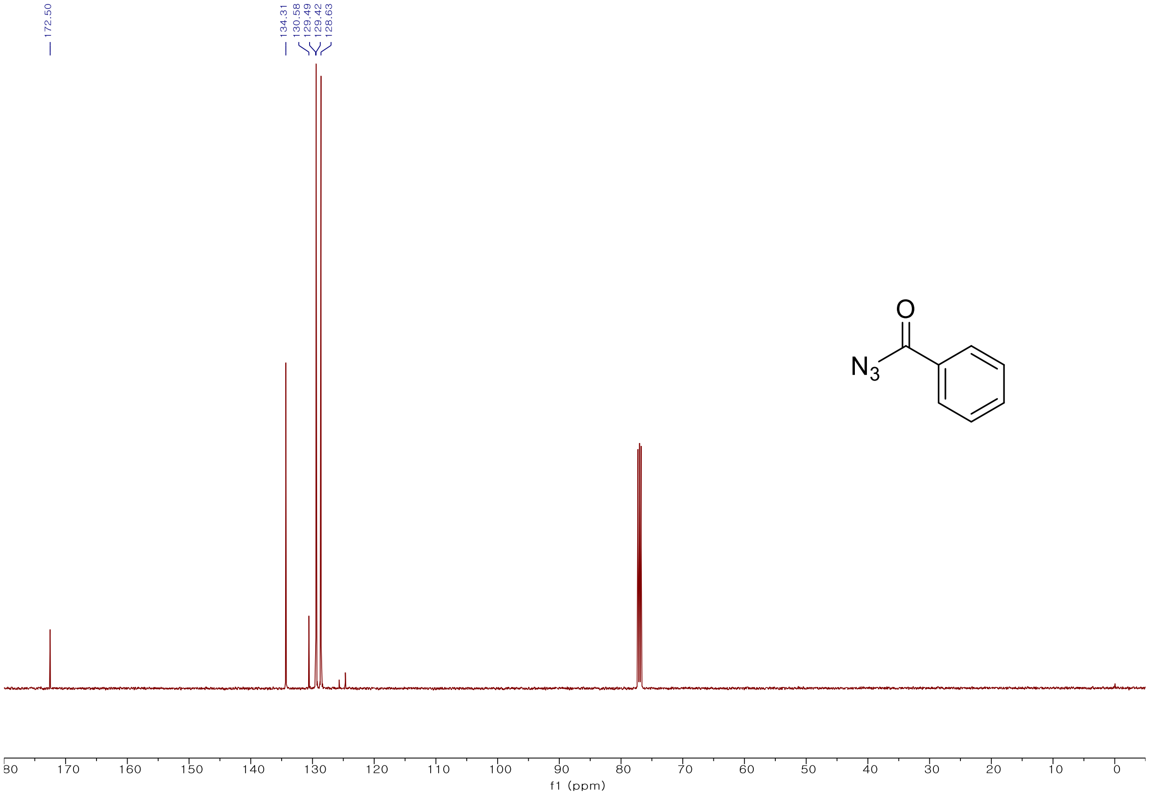

4-Bromobenzoyl azide (3b)

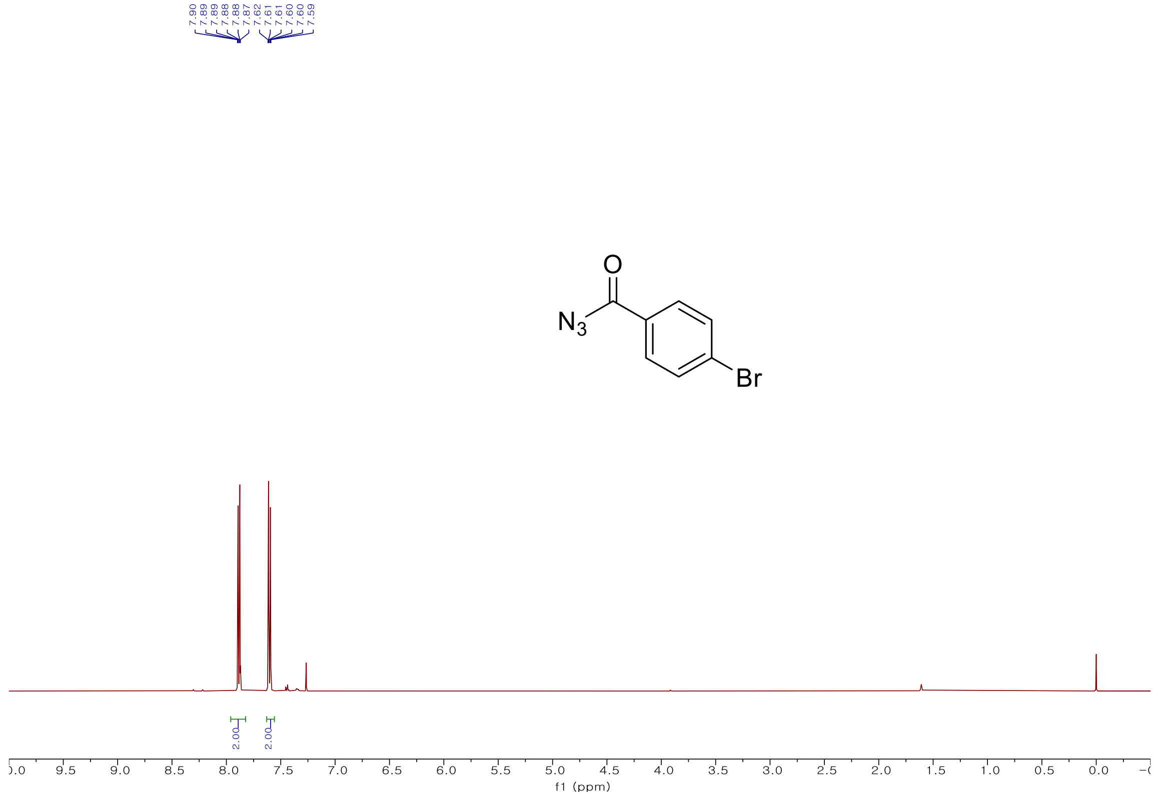

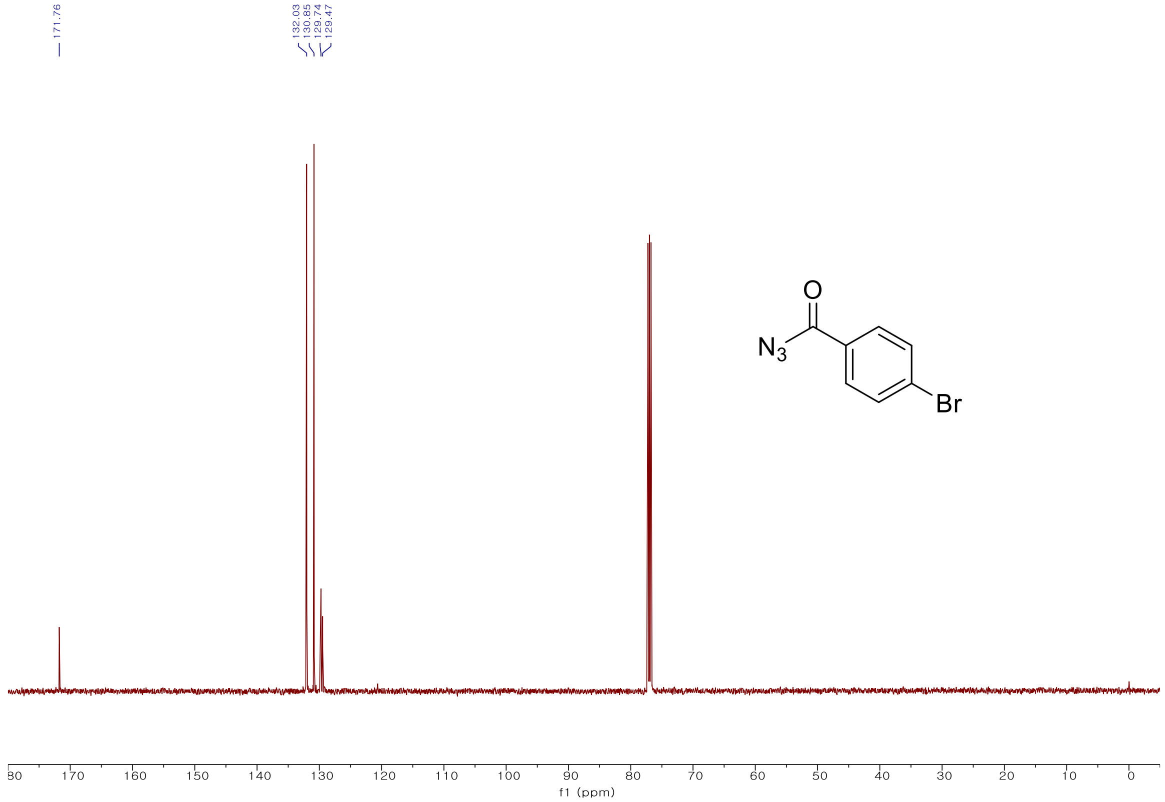

4-Chlorobenzoyl azide (3c).

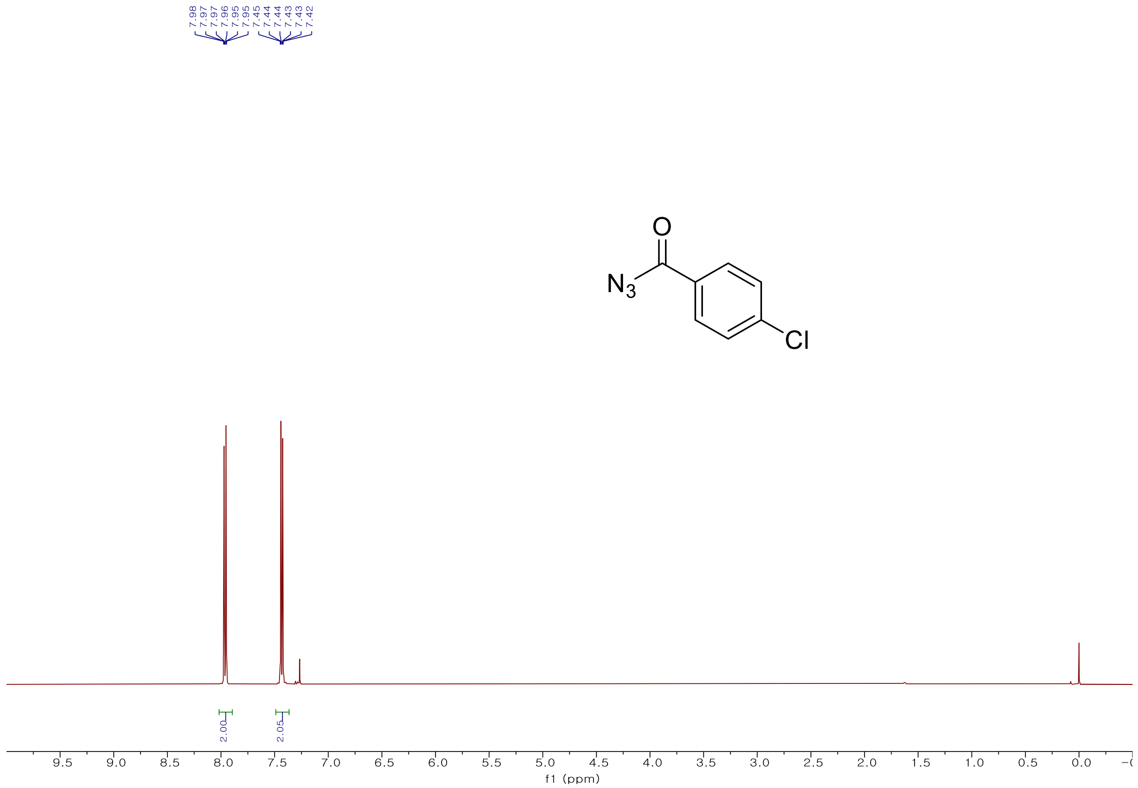

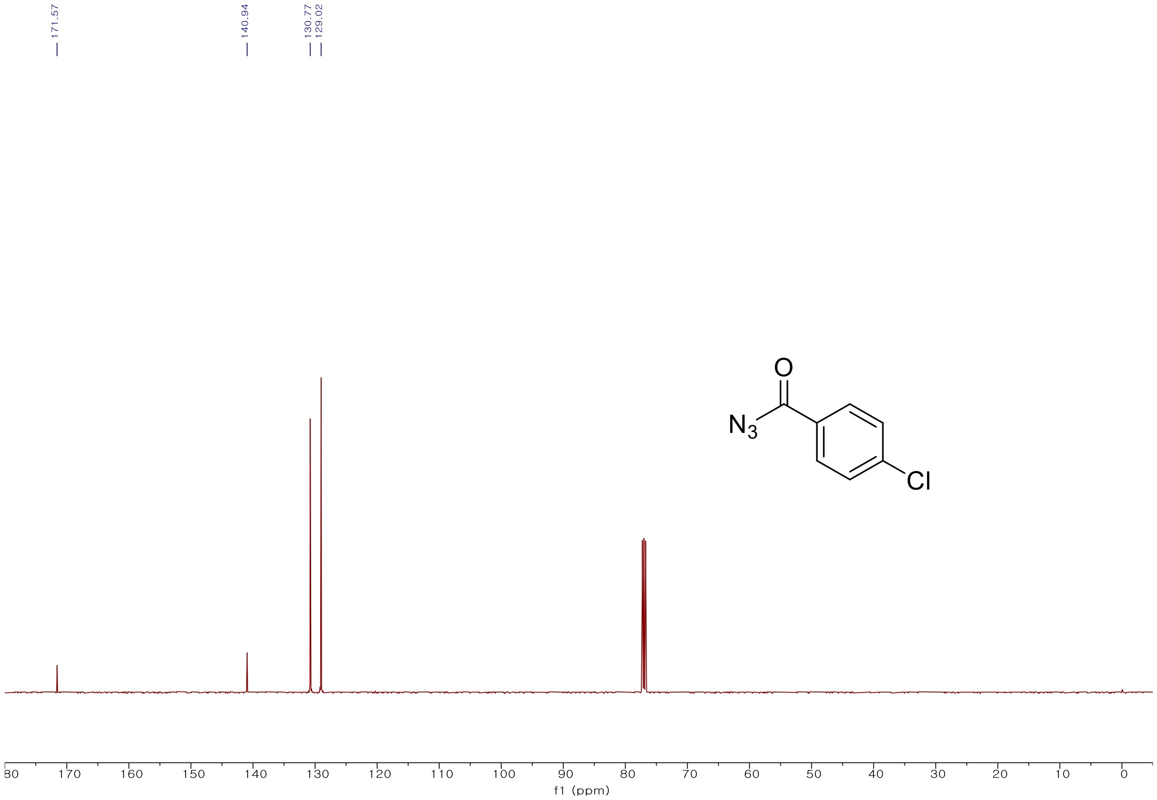

4-Fluorobenzoyl azide (3d)

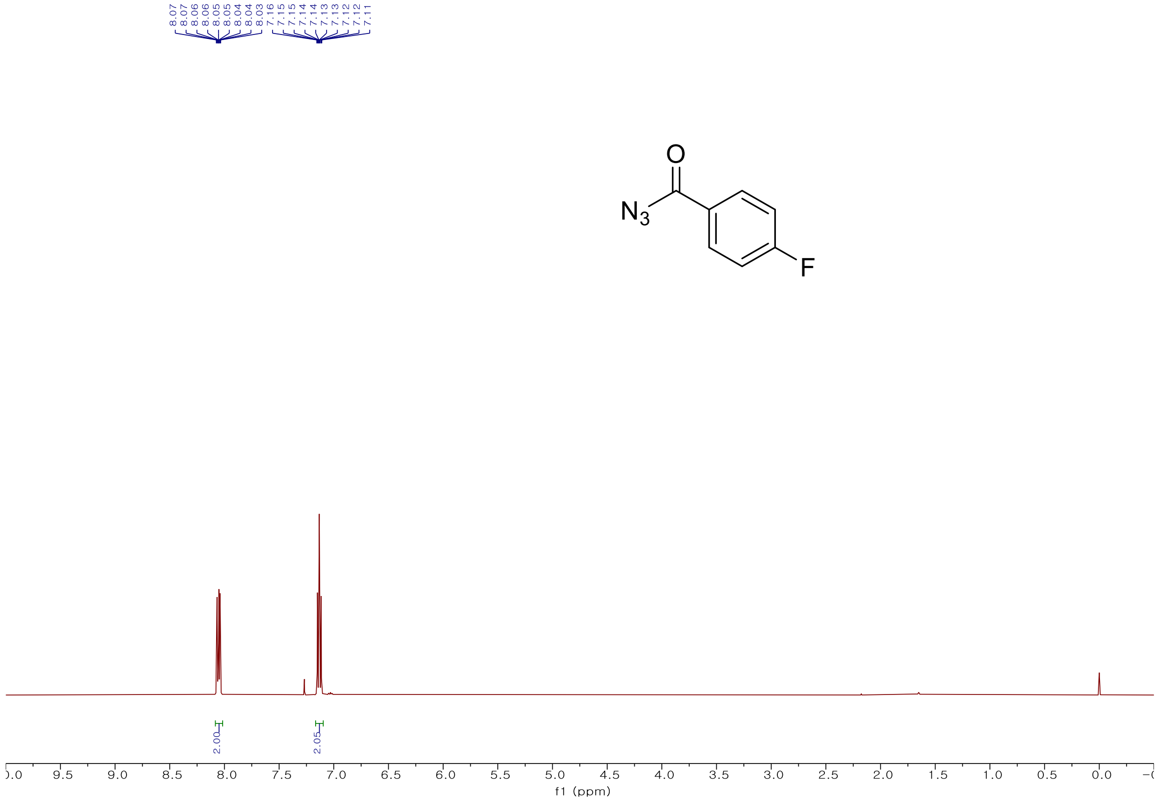

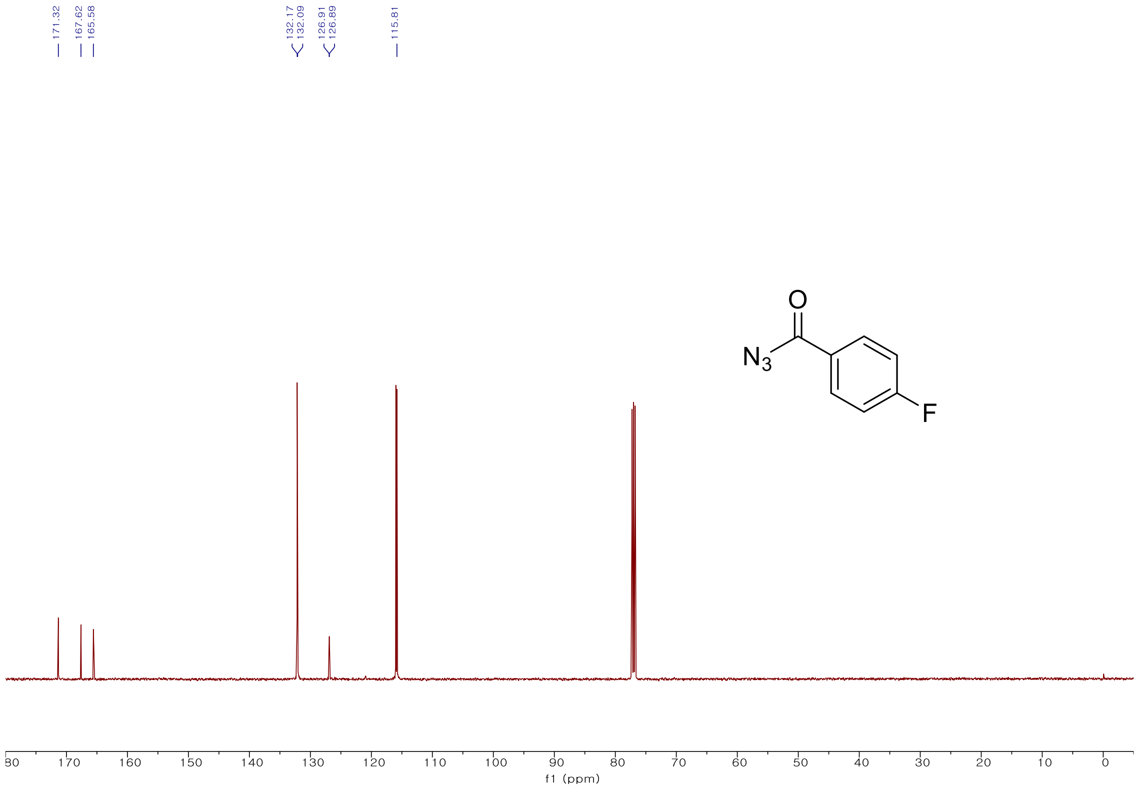

4-Methoxybenzoyl azide (3e)

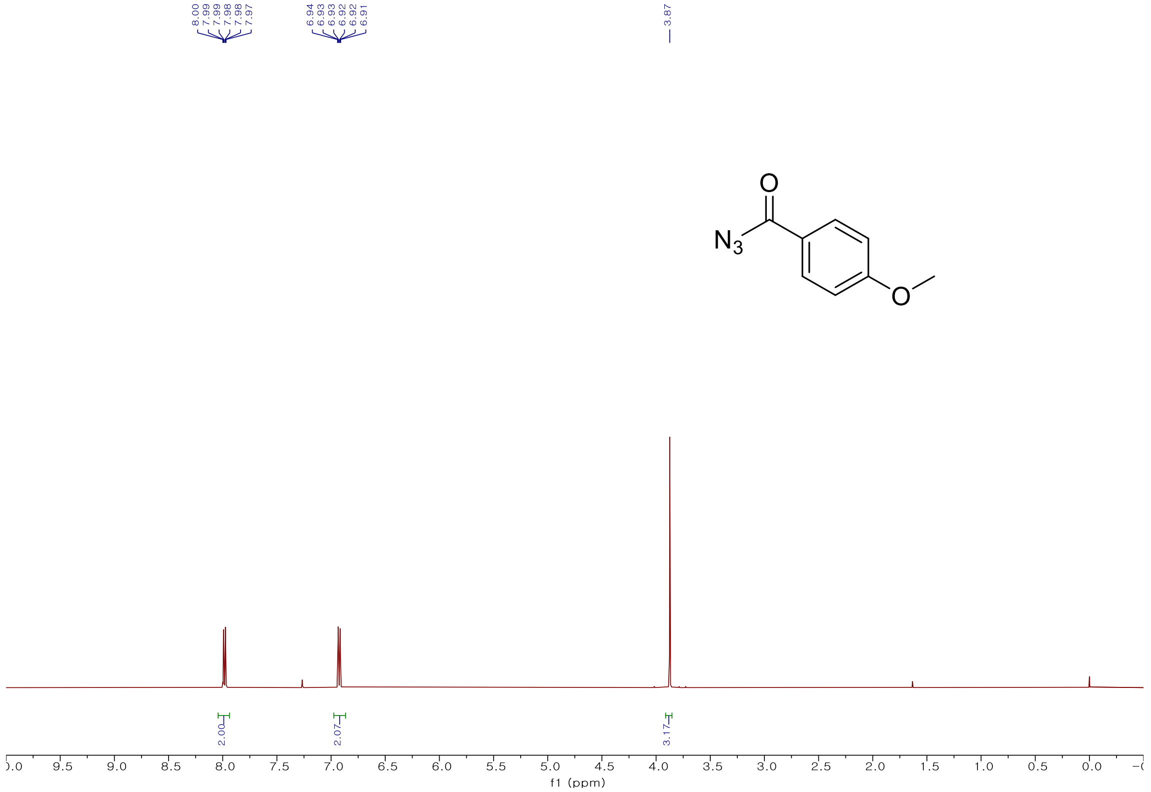

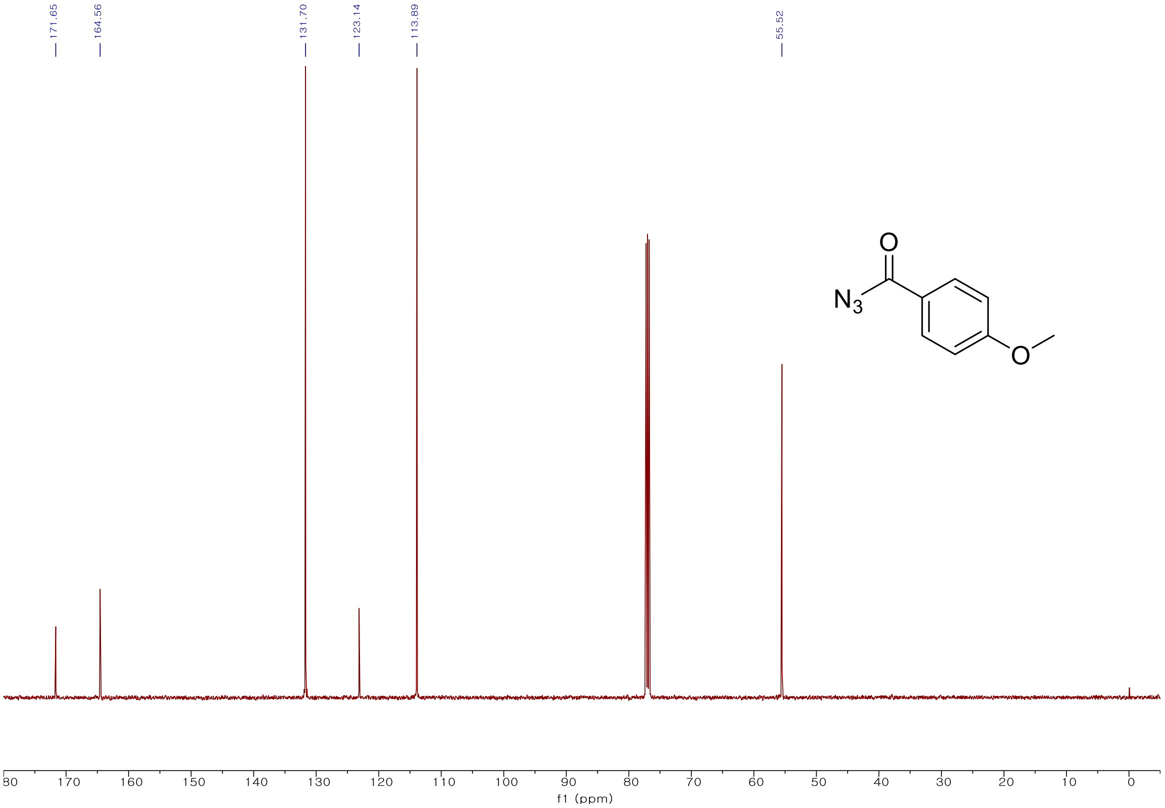

4-Nitrobenzoyl azide (3f)

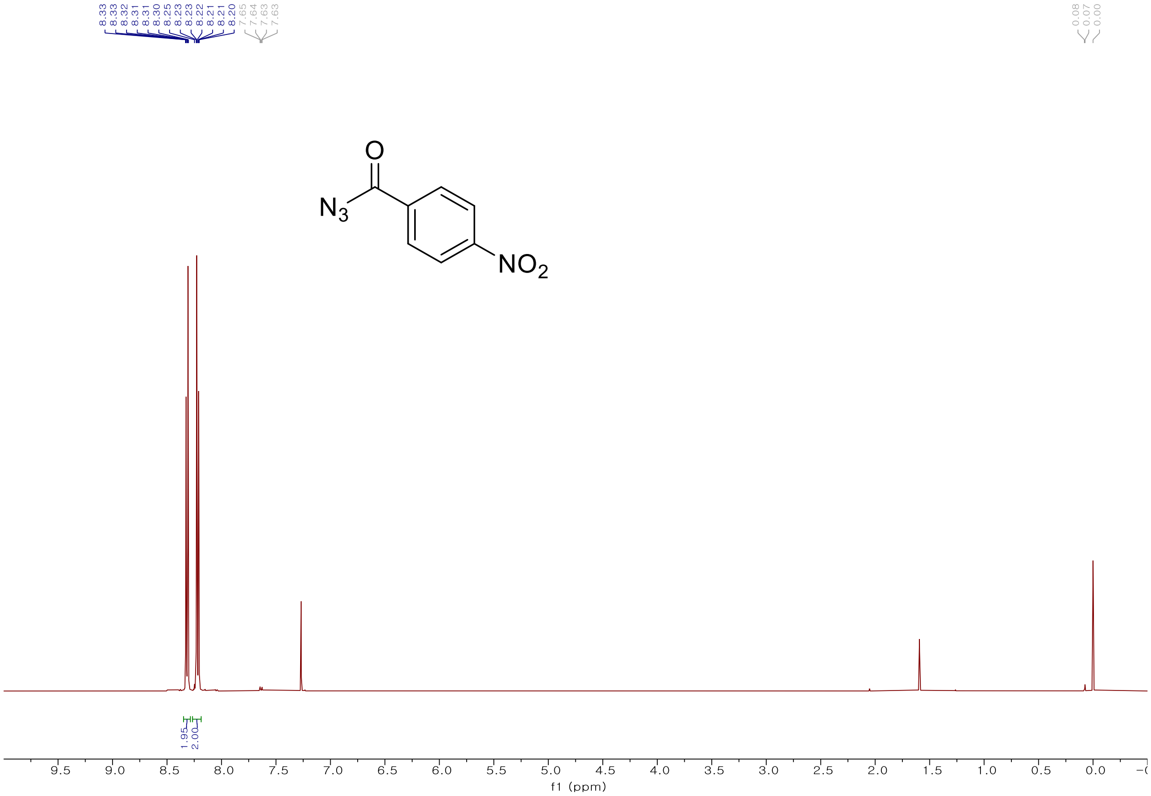

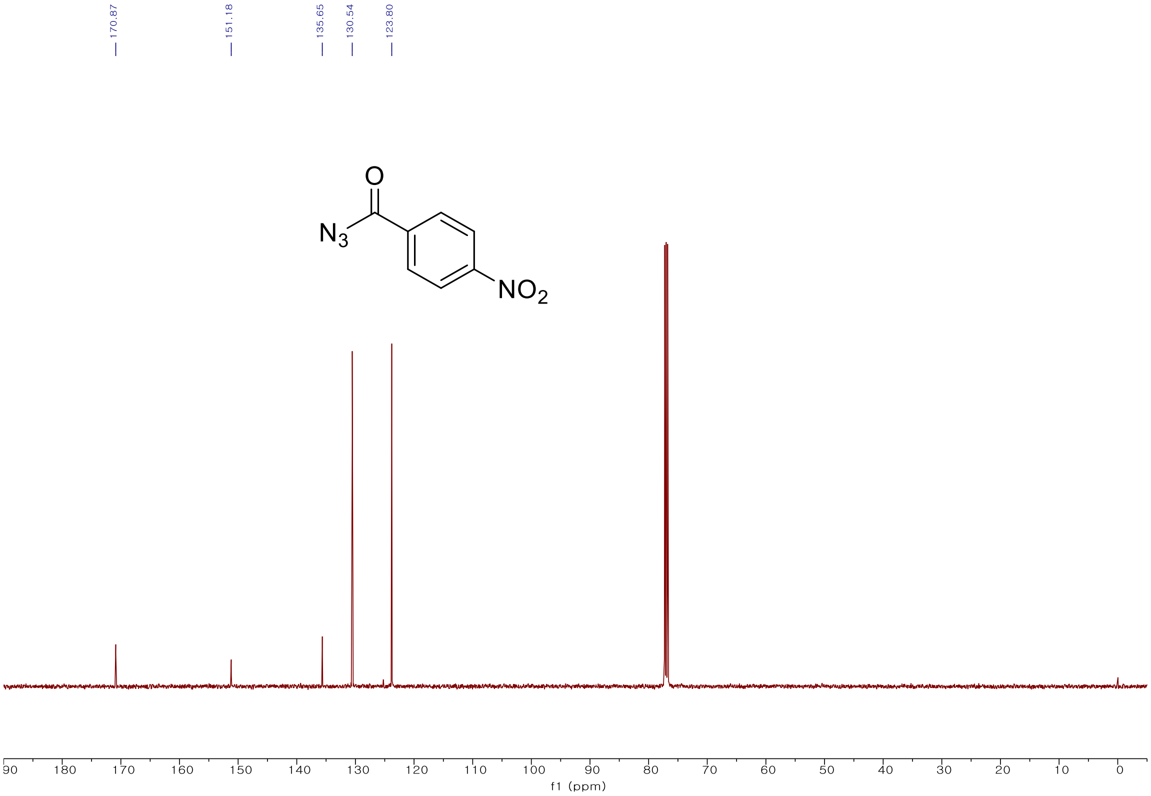

3,5-Dinitrobenzoyl azide (3g)

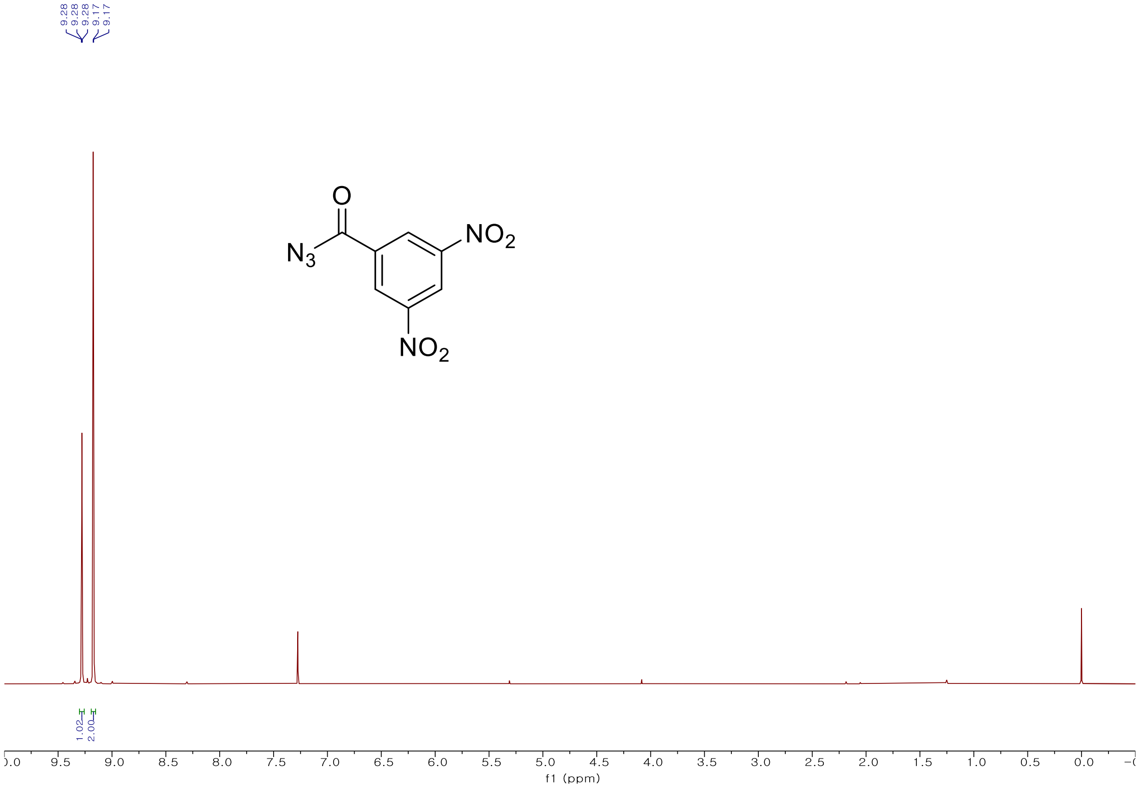

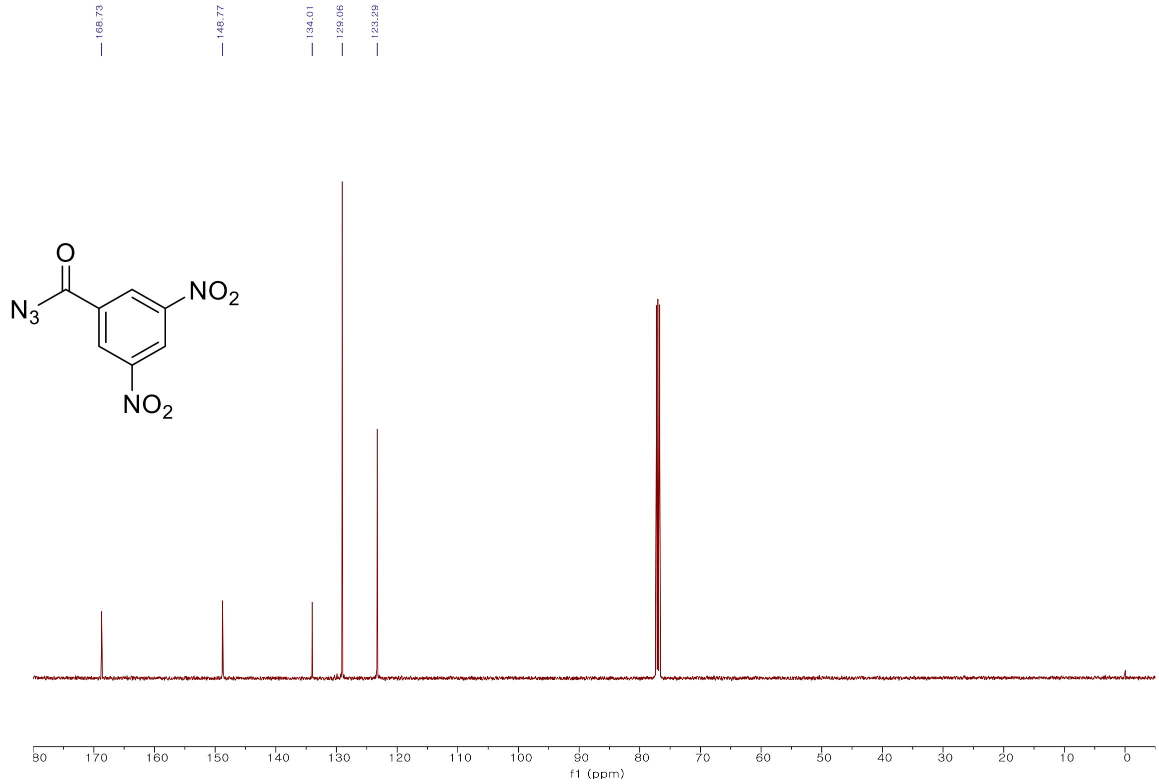

4-(Trifluoromethyl)benzoyl azide (3h)

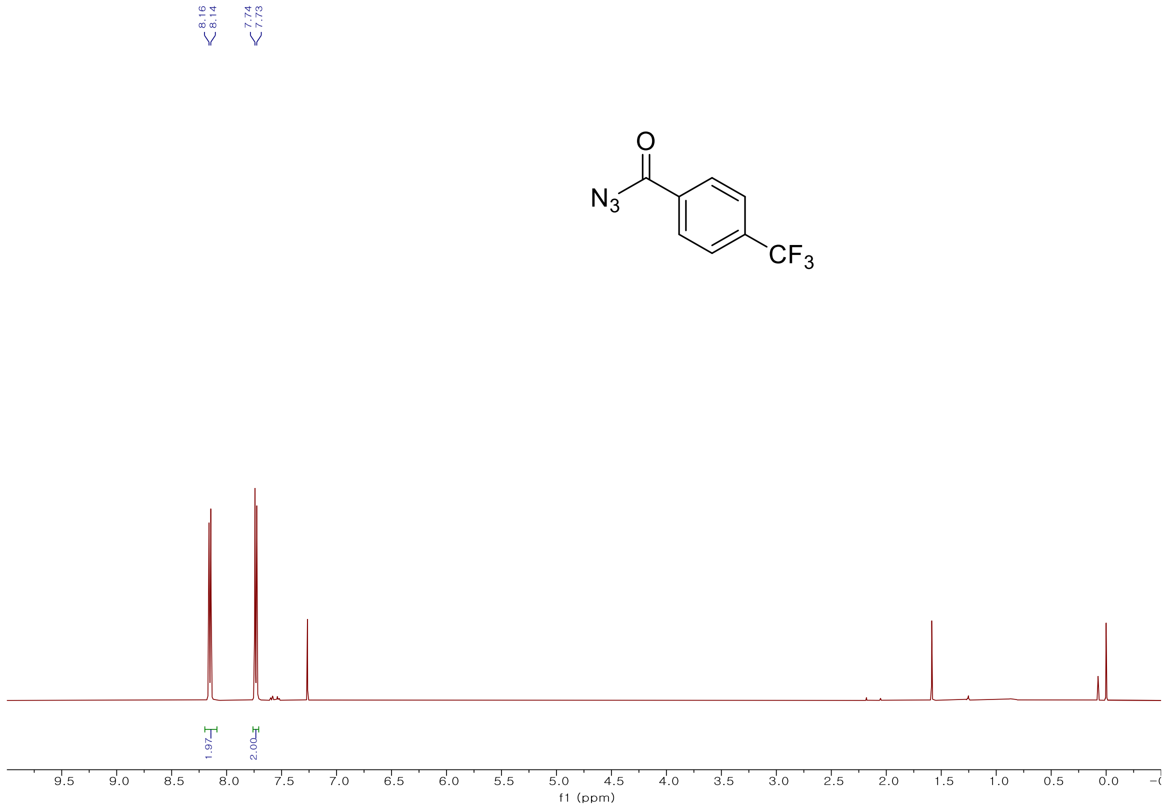

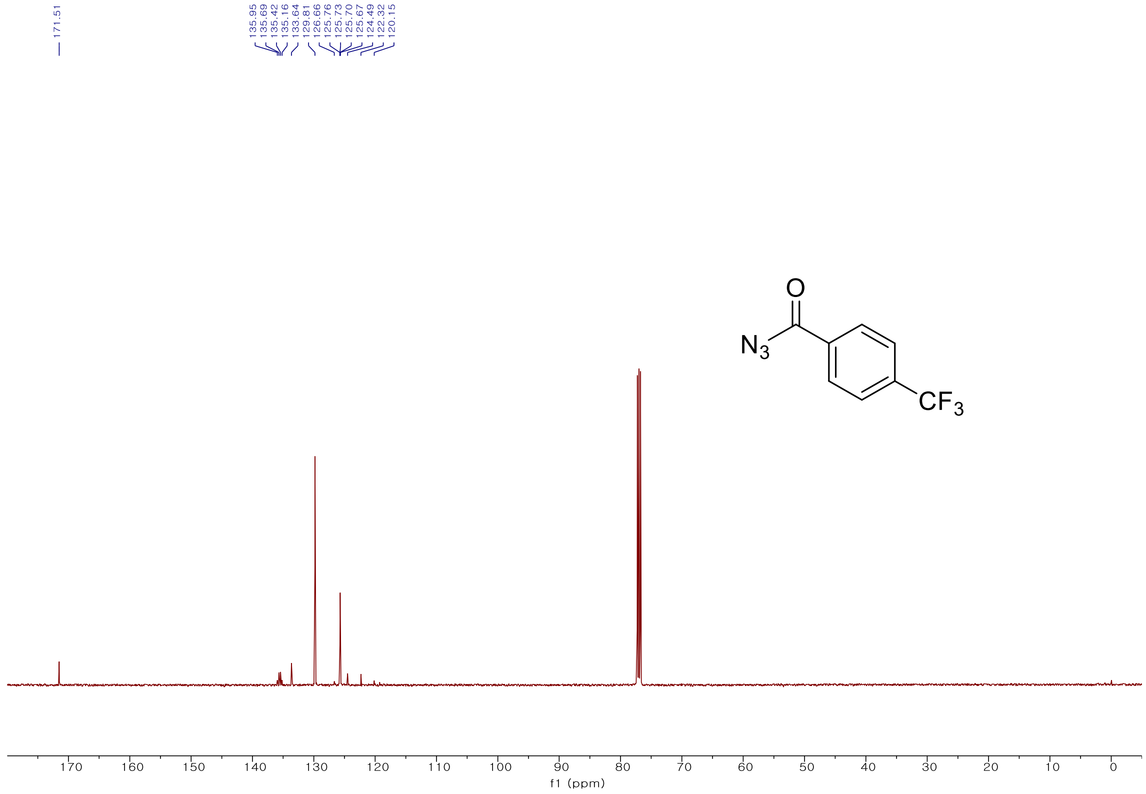

3,5-Bis(trifluoromethyl)benzoyl azide (3i)

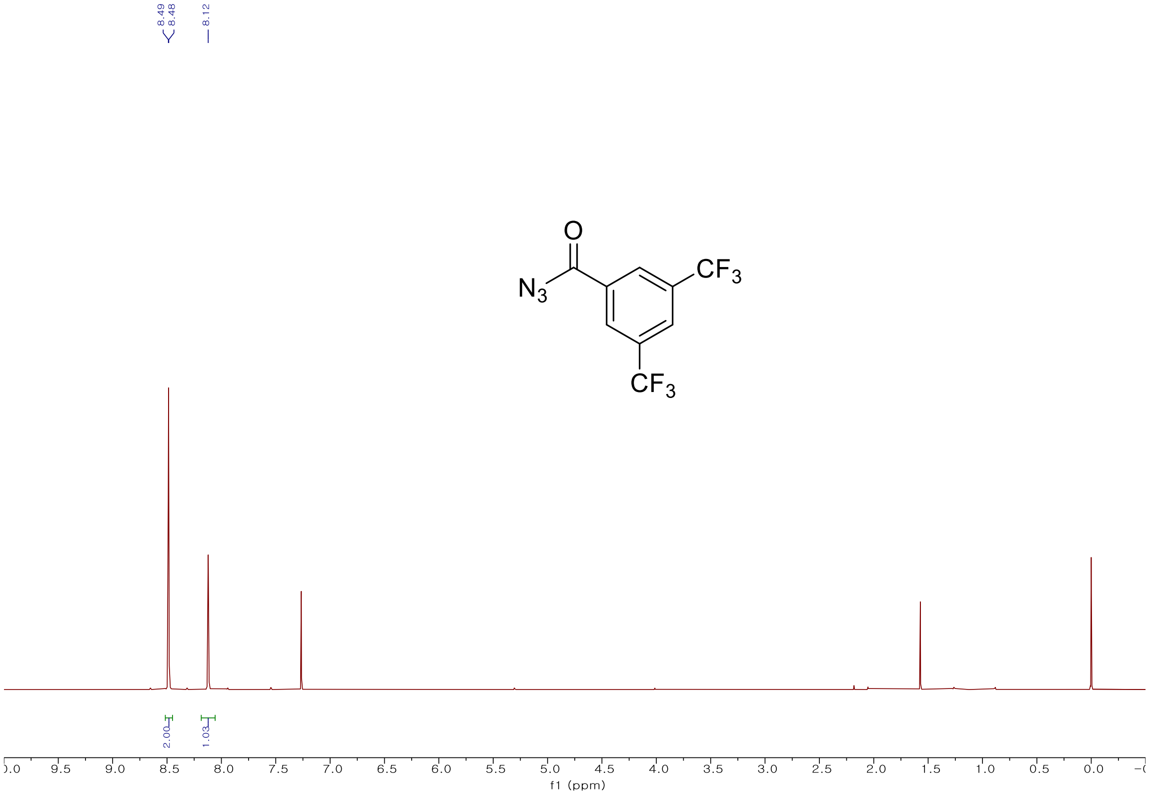

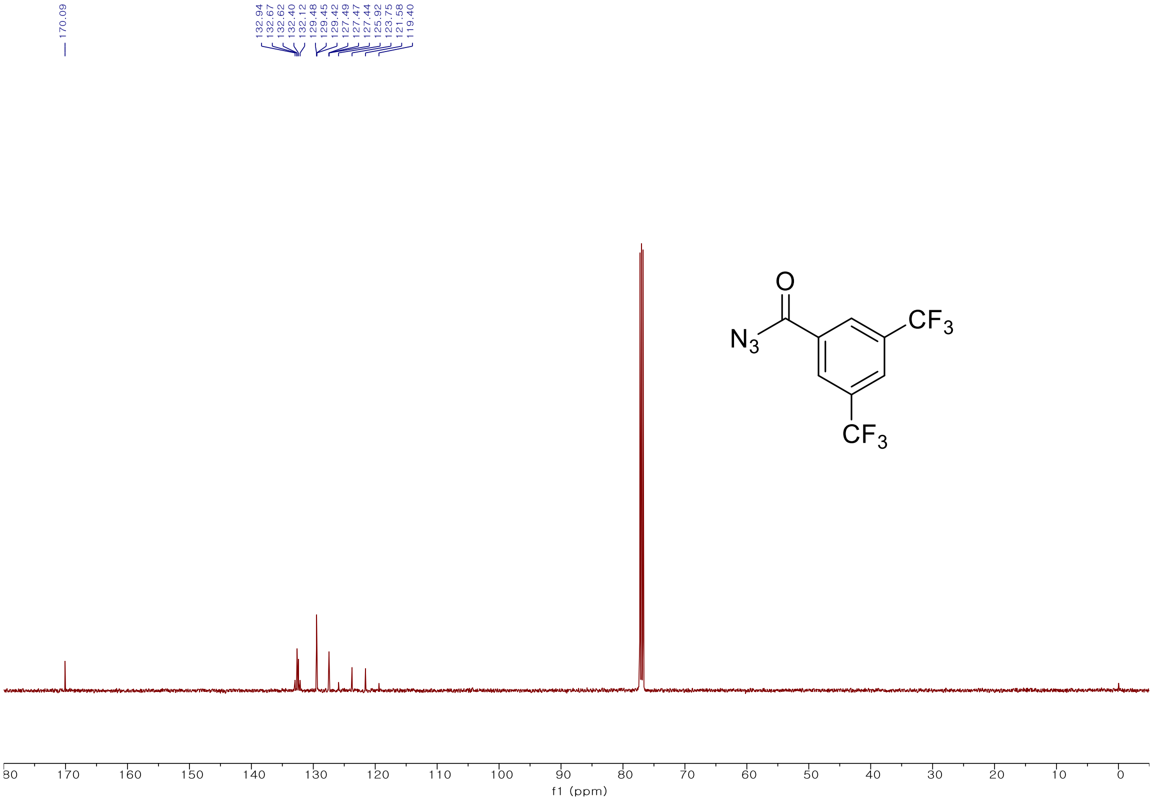

(*Z*)-*N*-(1,4-Dihydroisoquinolin-3(2*H*)-ylidene)benzamide (Scheme 2, 4a)

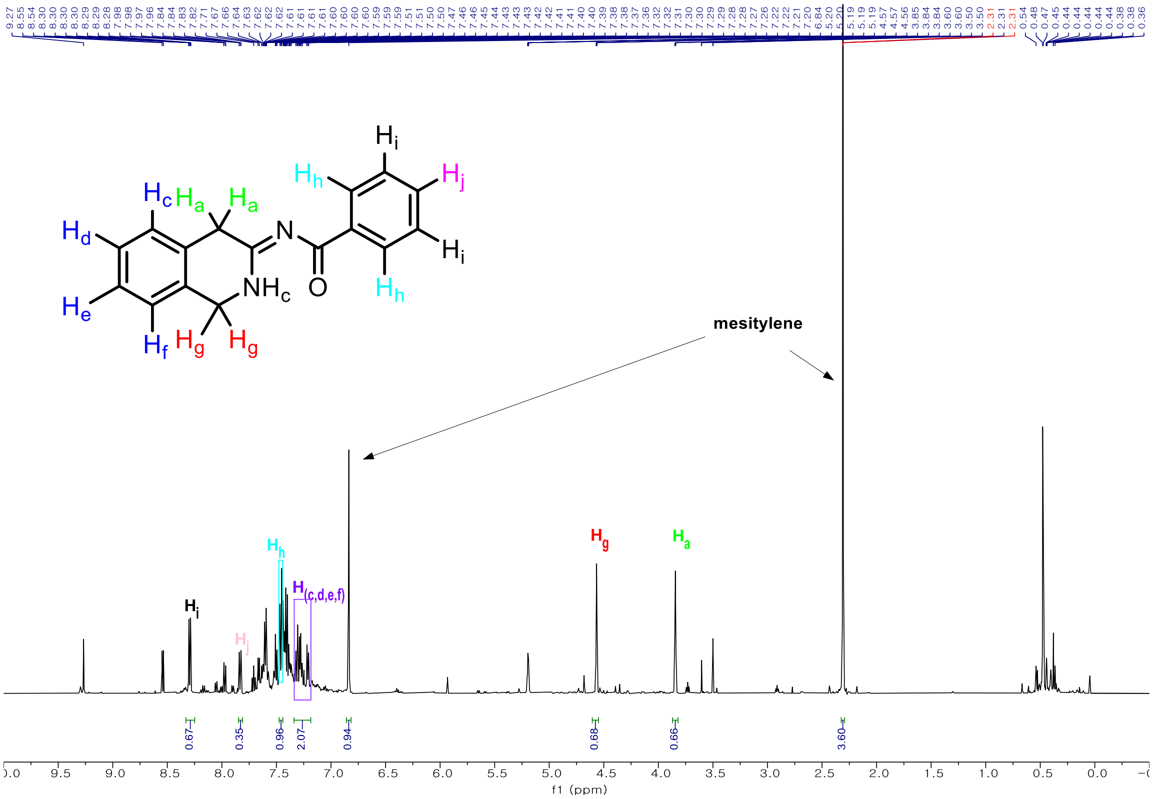

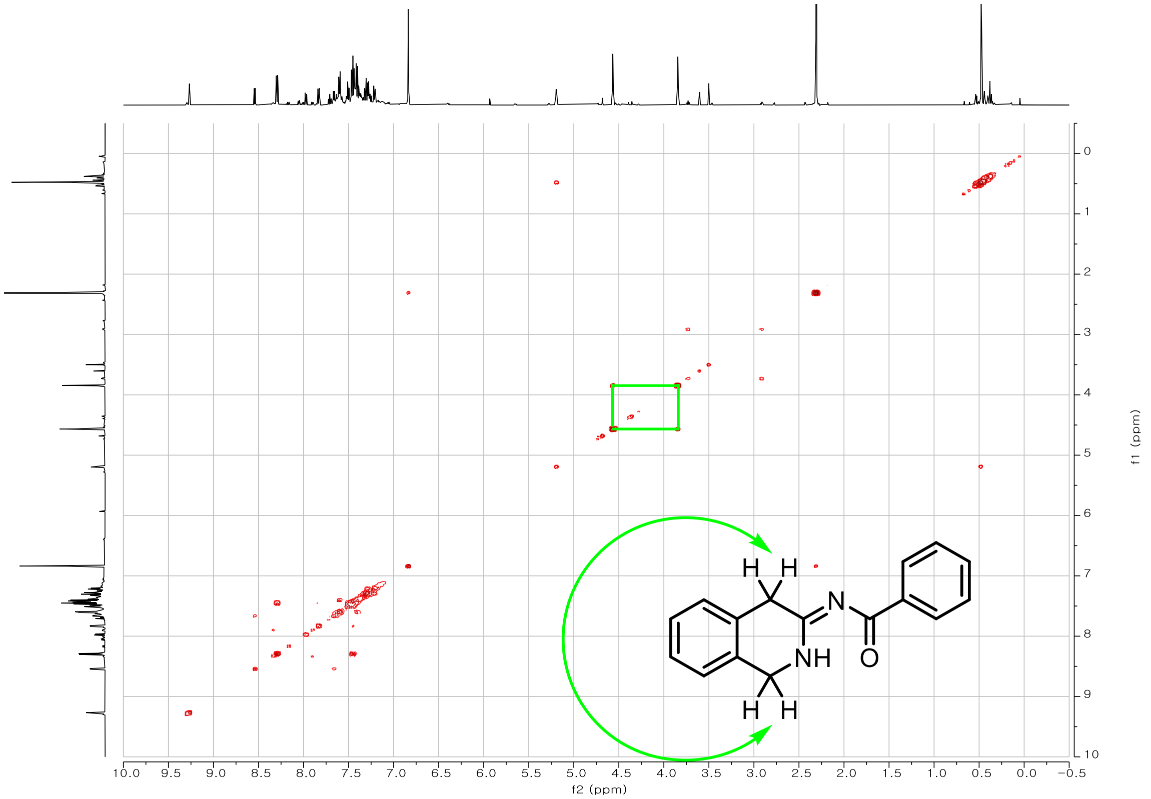

(*Z*)-4-Bromo-*N*-(1,4-dihydroisoquinolin-3(2*H*)-ylidene)benzamide (Scheme 2, 4b)

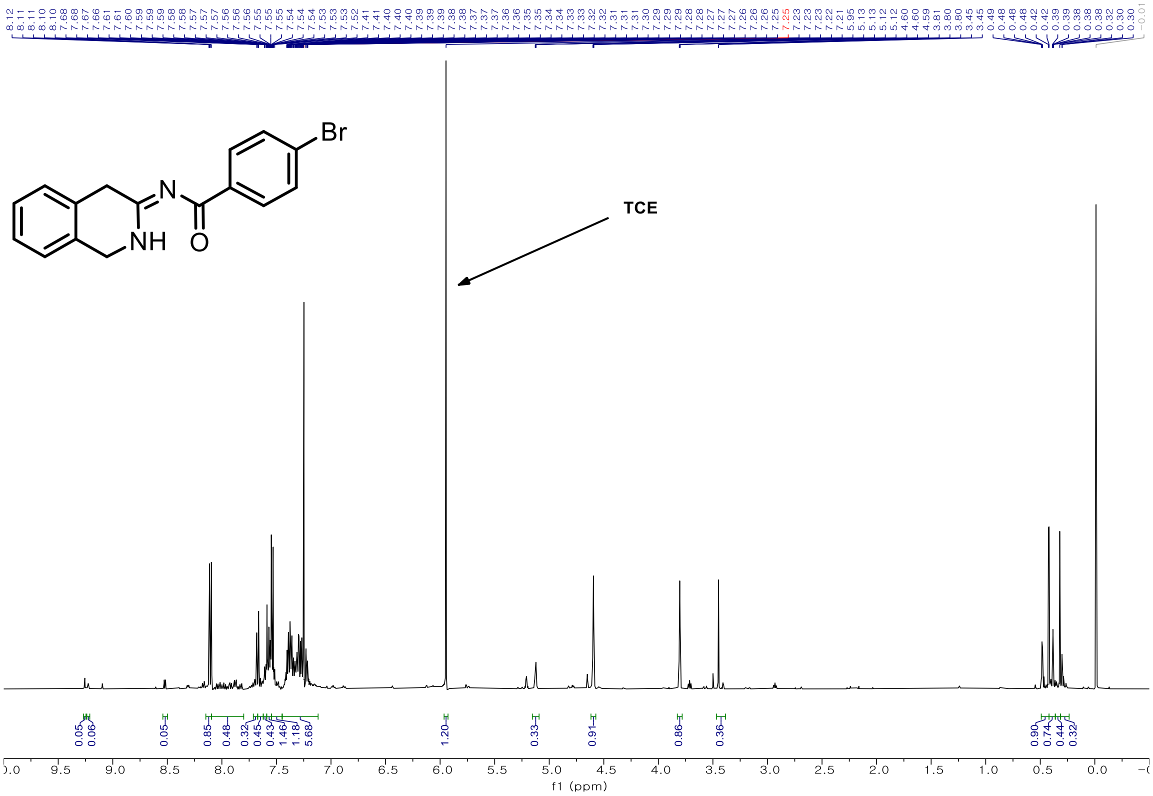

(*Z*)-4-Chloro-*N*-(1,4-dihydroisoquinolin-3(2*H*)-ylidene)benzamide (Scheme 2, 4c)

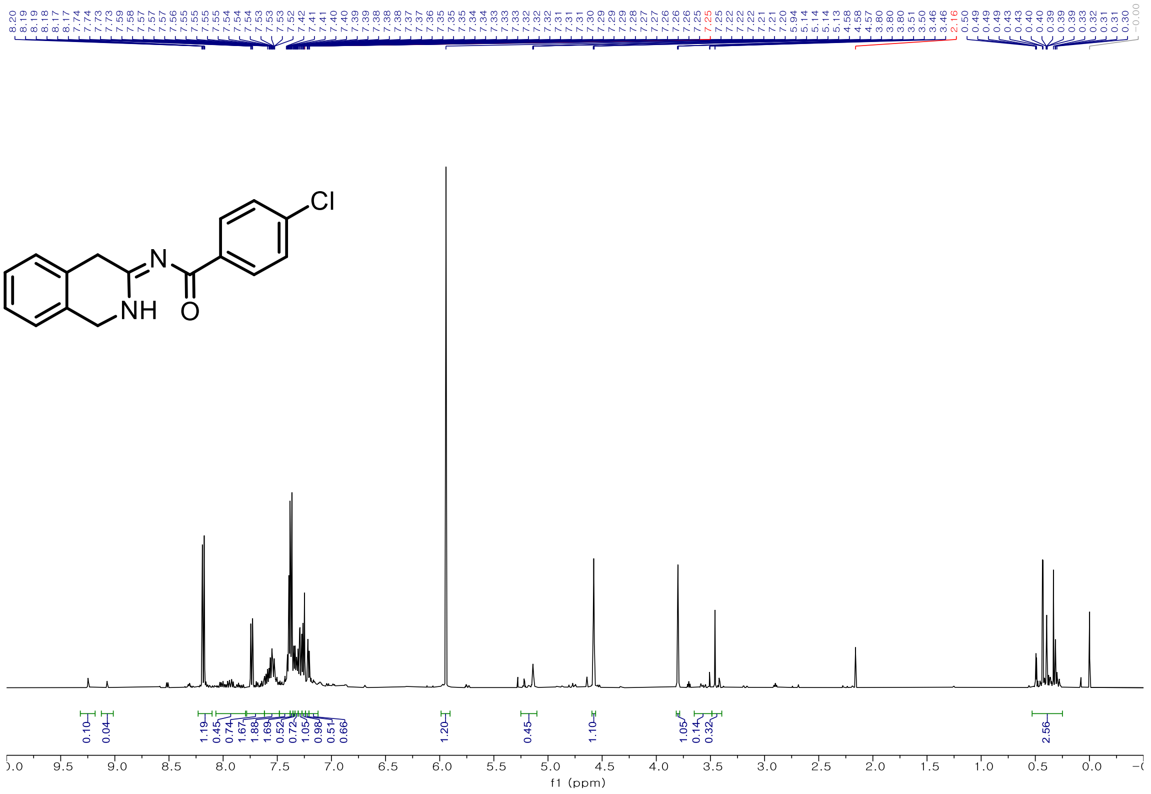

(*Z*)-*N*-(1,4-Dihydroisoquinolin-3(2*H*)-ylidene)-4-fluorobenzamide (Scheme 2, 4d)

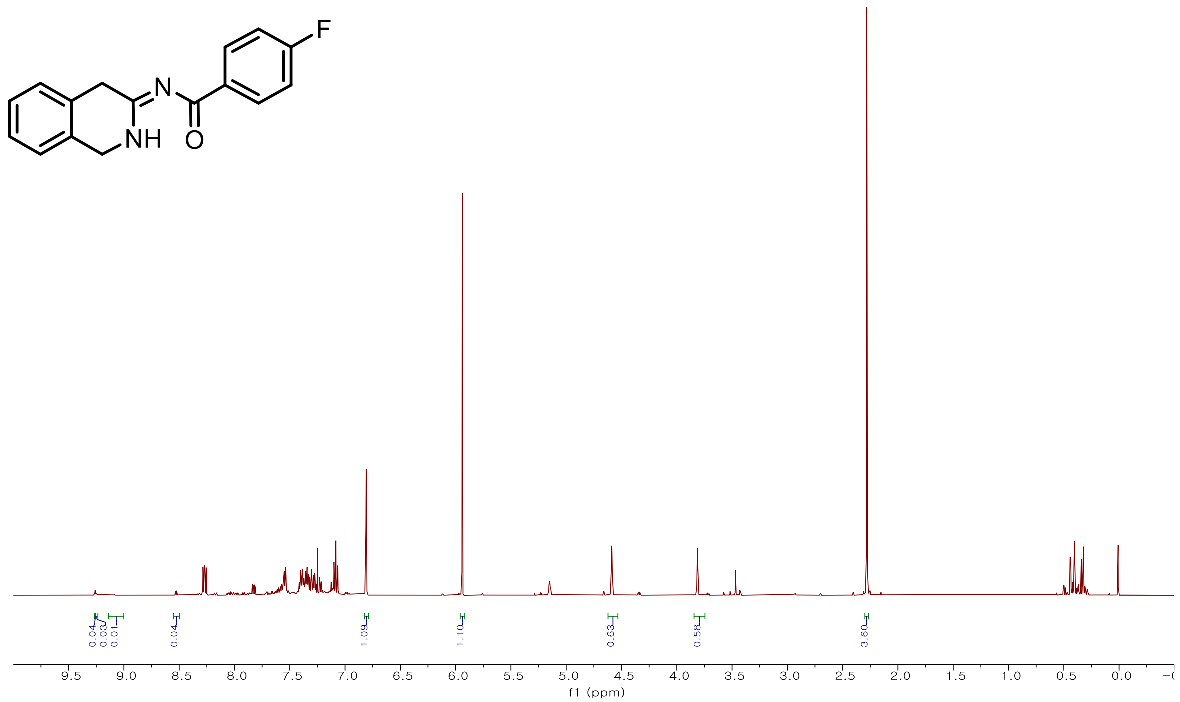

(*Z*)-*N*-(1,4-Dihydroisoquinolin-3(2*H*)-ylidene)-4-methoxybenzamide (Scheme 2, 4e)

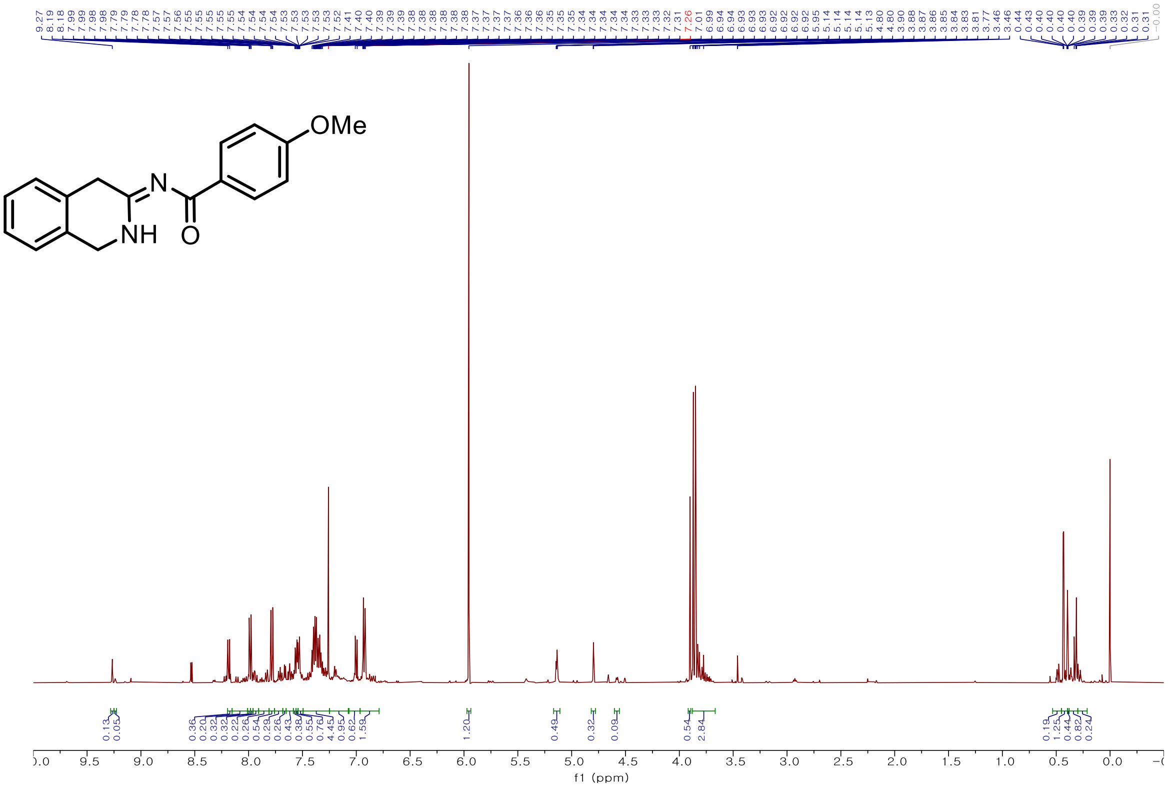

(*Z*)-*N*-(1,4-Dihydroisoquinolin-3(2*H*)-ylidene)-4-nitrobenzamide (Scheme 2, 4f)

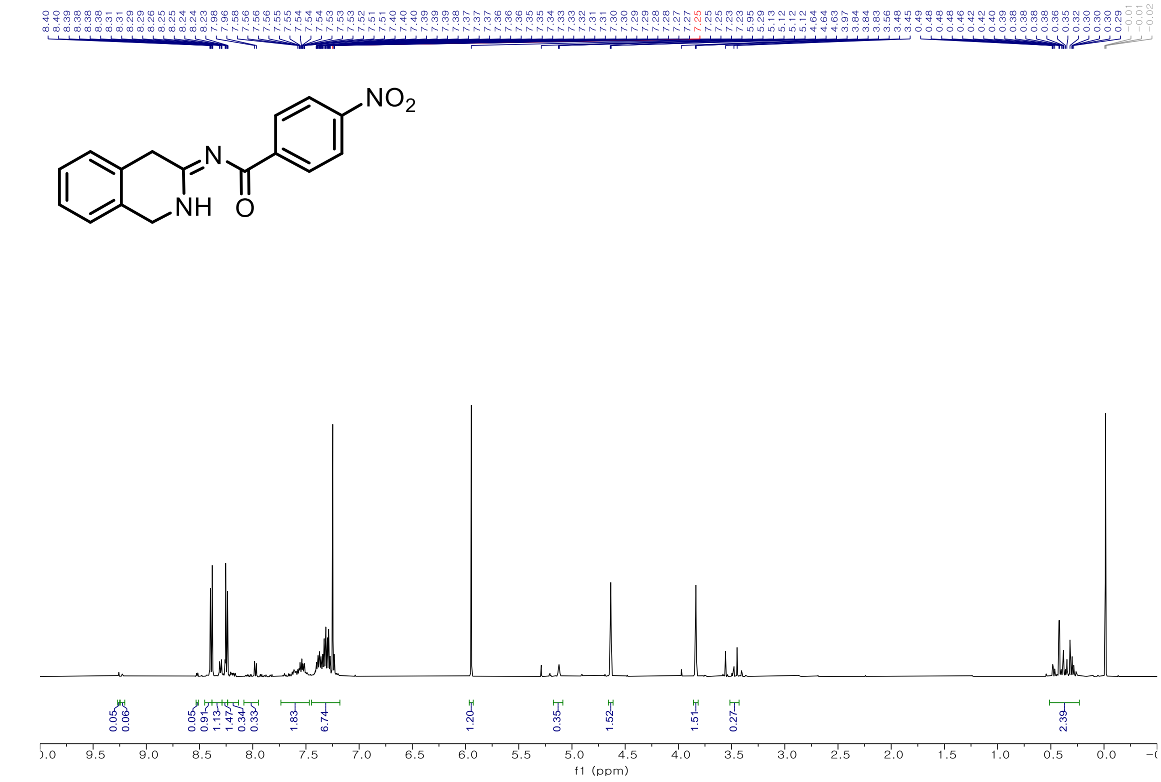

(*Z*)-*N*-(1,4-Dihydroisoquinolin-3(2*H*)-ylidene)-3,5-dinitrobenzamide (Scheme 2, 4g)

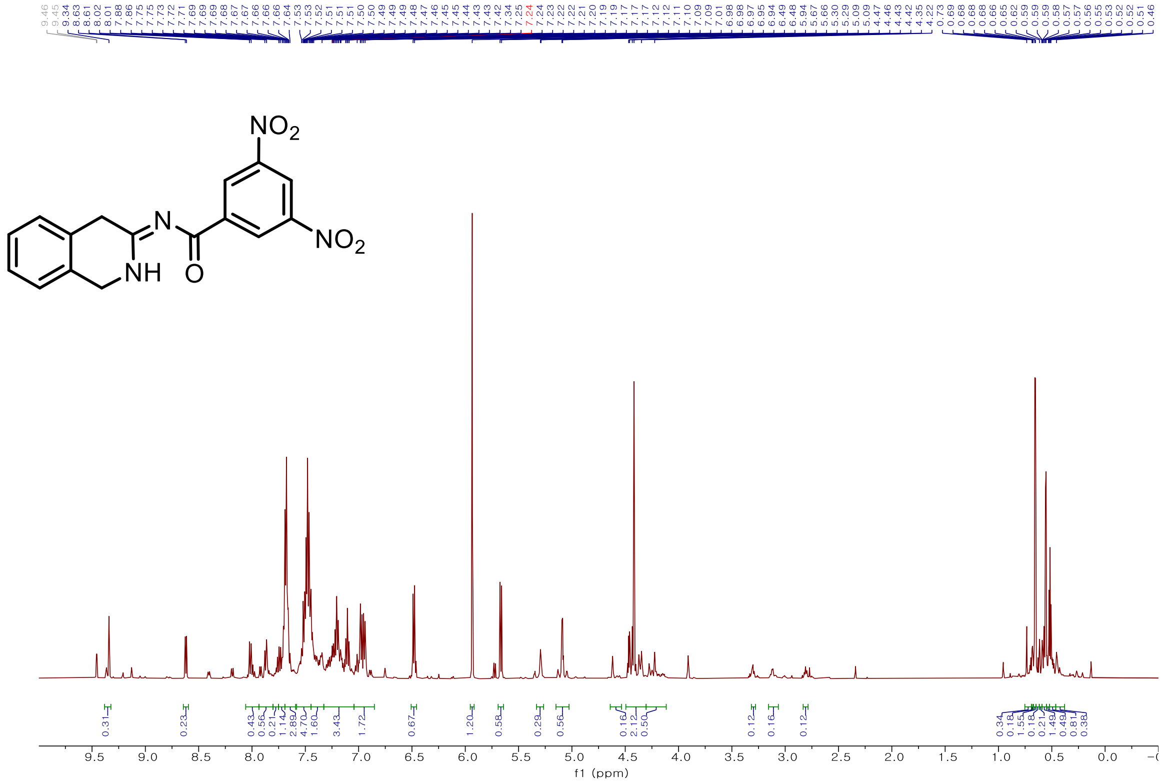

(*Z*)-*N*-(1,4-Dihydroisoquinolin-3(2*H*)-ylidene)-4-(trifluoromethyl)benzamide (Scheme 2, 4h)

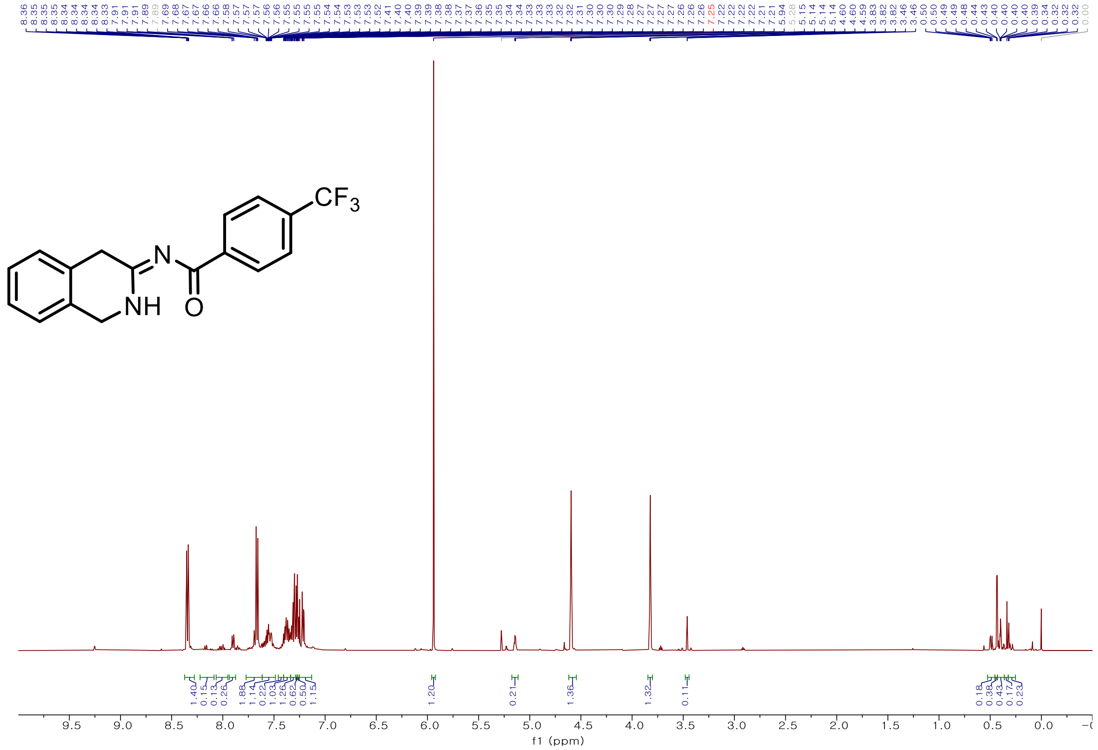

(*Z*)-*N*-(1,4-Dihydroisoquinolin-3(2*H*)-ylidene)-3,5-bis(trifluoromethyl)benzamide (Scheme 3, 4i)

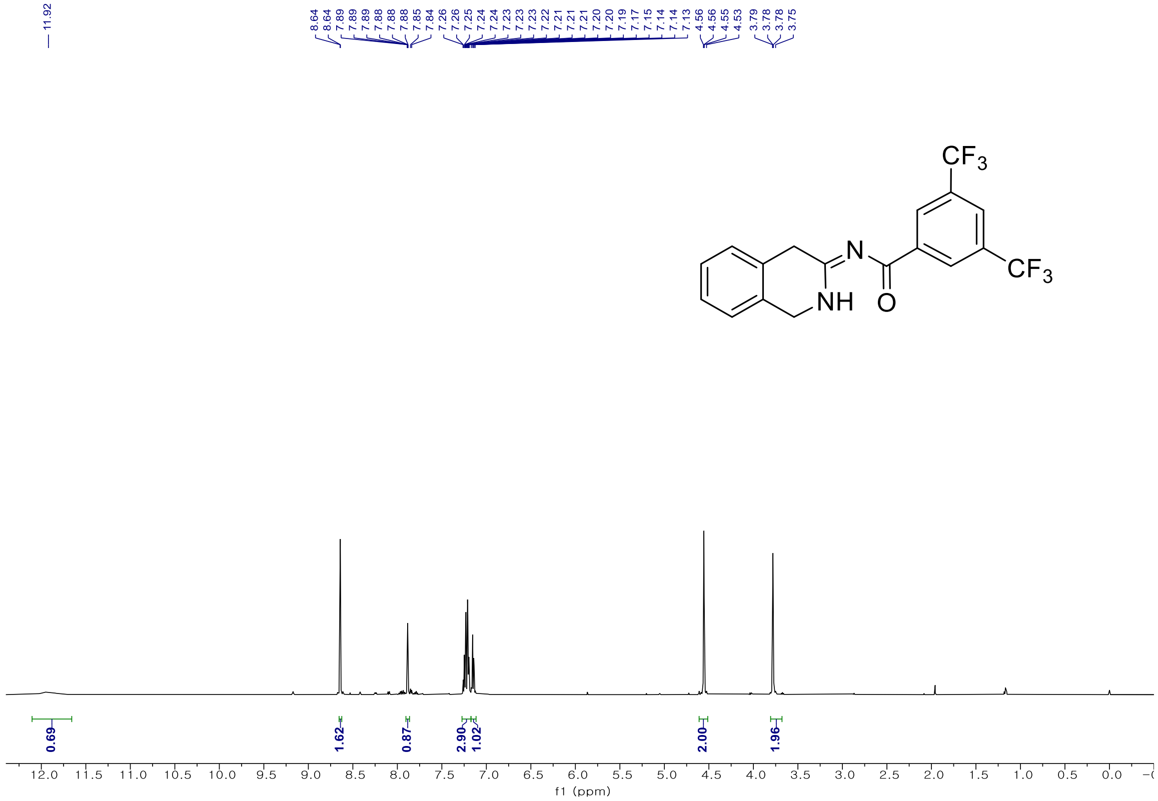

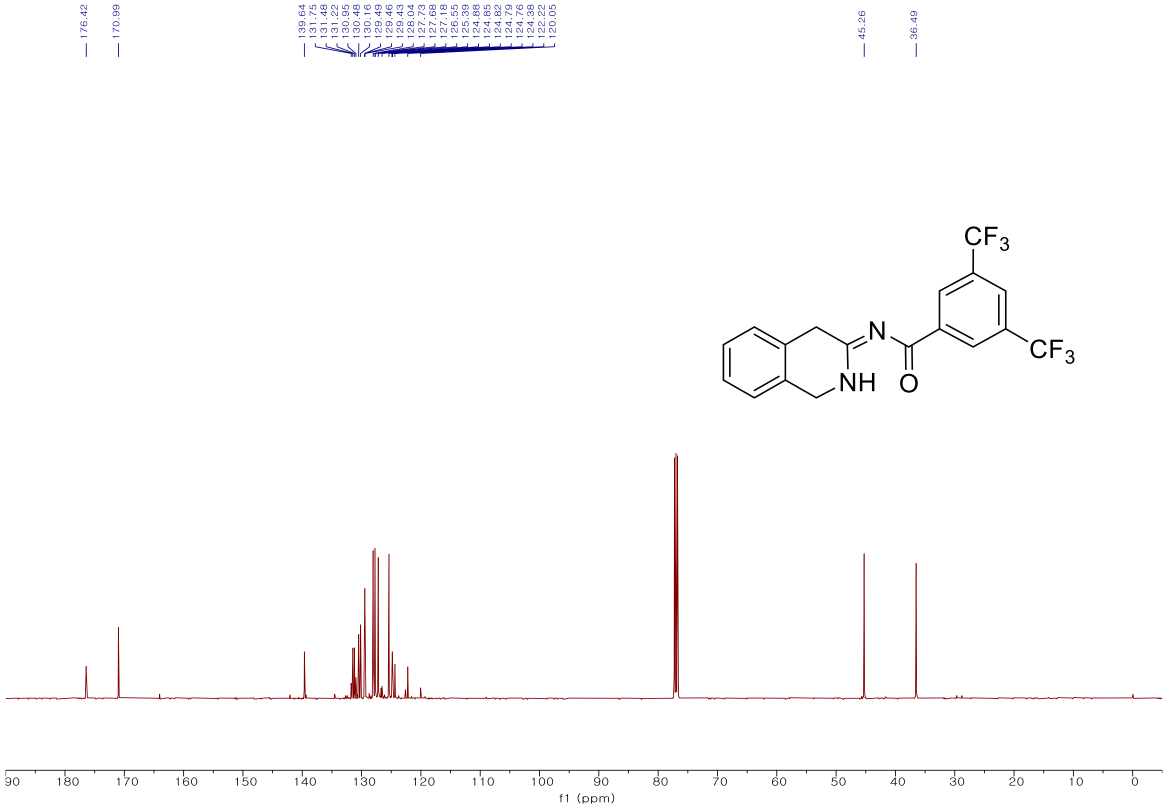

(*Z*)-*N*-(5-Chloro-1,4-dihydroisoquinolin-3(2*H*)-ylidene)-3,5-bis(trifluoromethyl)benzamide (Scheme 3, 4j)

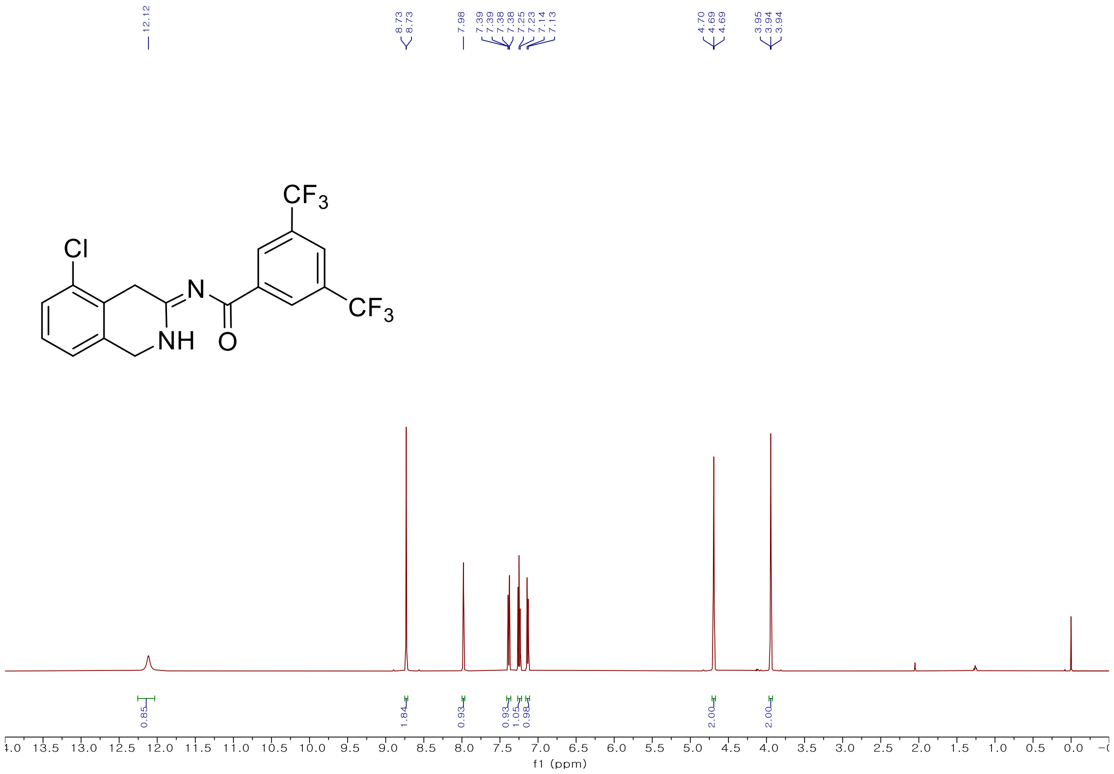

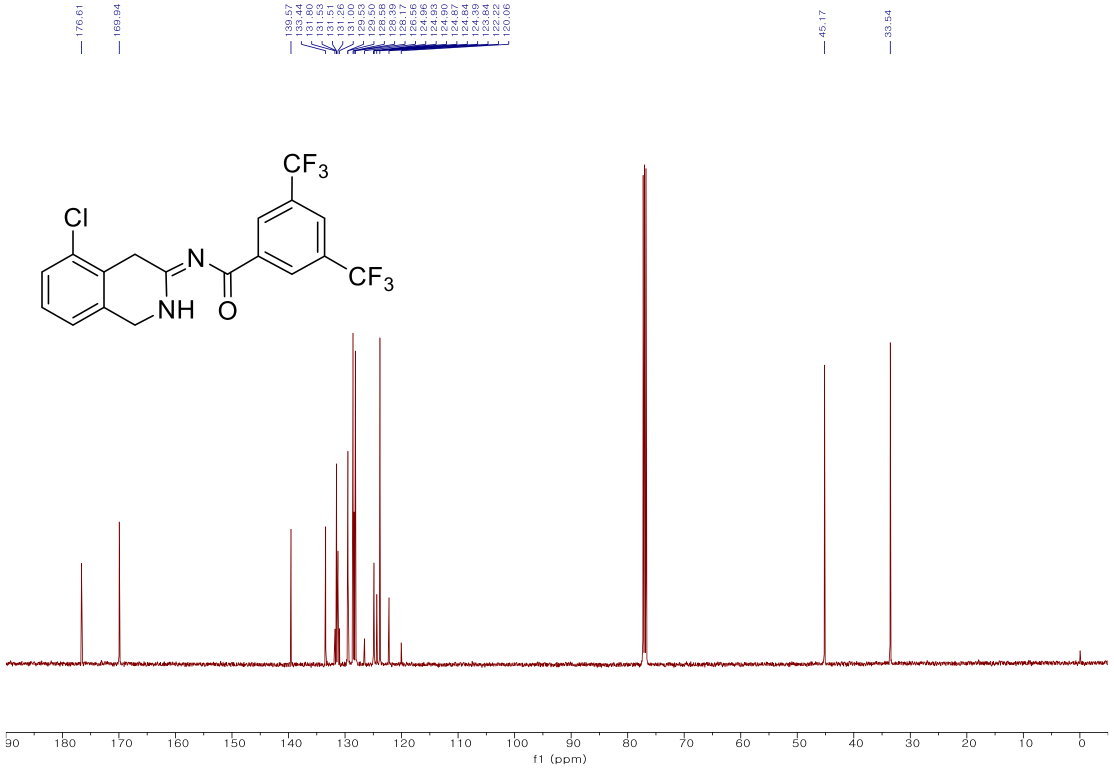

(*Z*)-*N*-(8-Bromo-1,4-dihydroisoquinolin-3(2*H*)-ylidene)-3,5-bis(trifluoromethyl)benzamide (Scheme 3, 4k)

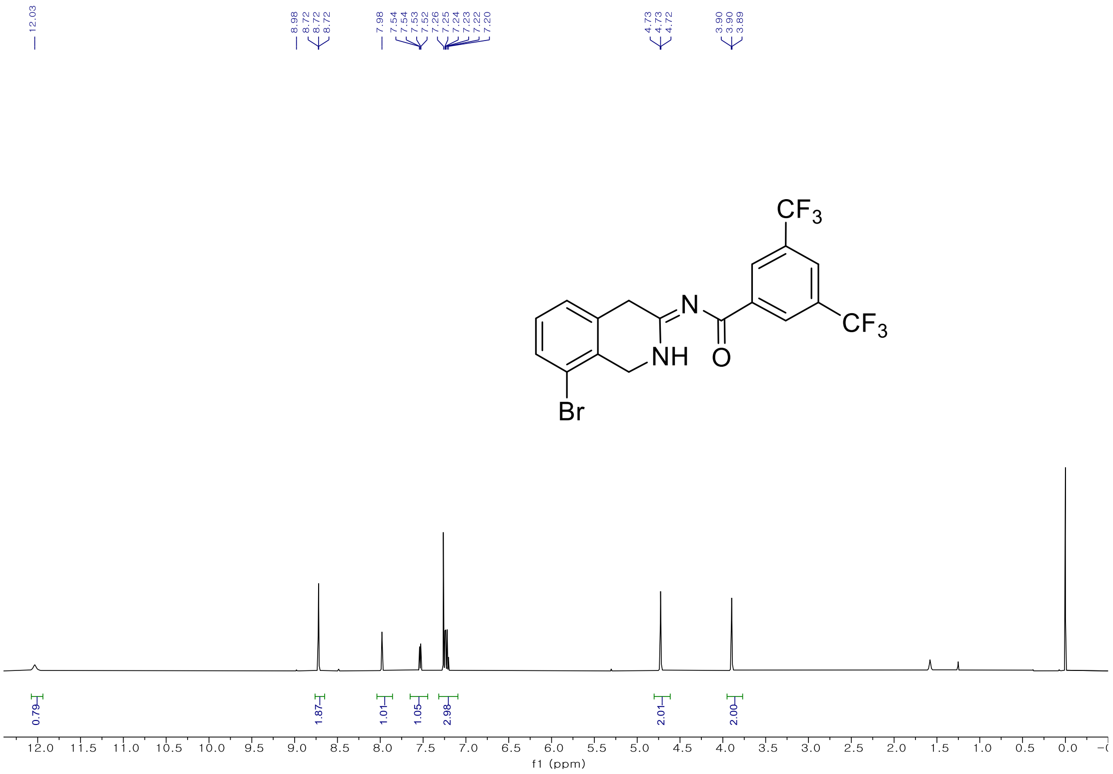

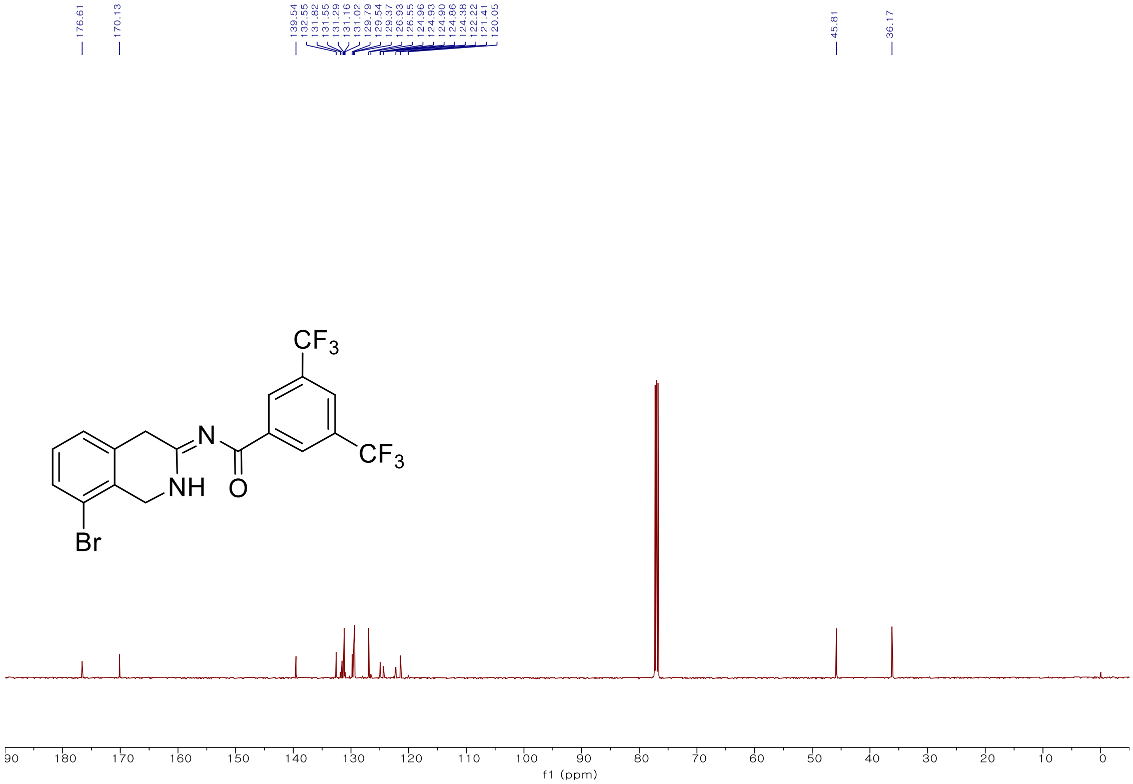

(*Z*)-*N*-(5-Bromo-1,4-dihydroisoquinolin-3(2*H*)-ylidene)-3,5-bis(trifluoromethyl)benzamide (Scheme 3, 4l)

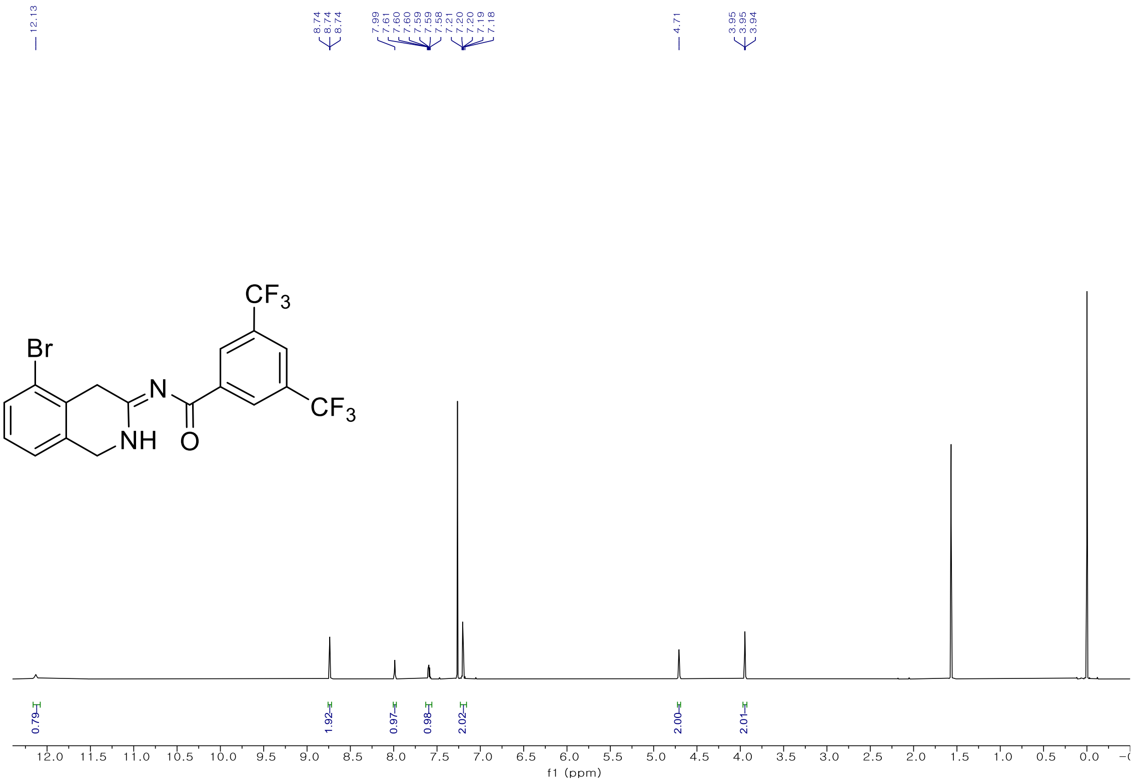

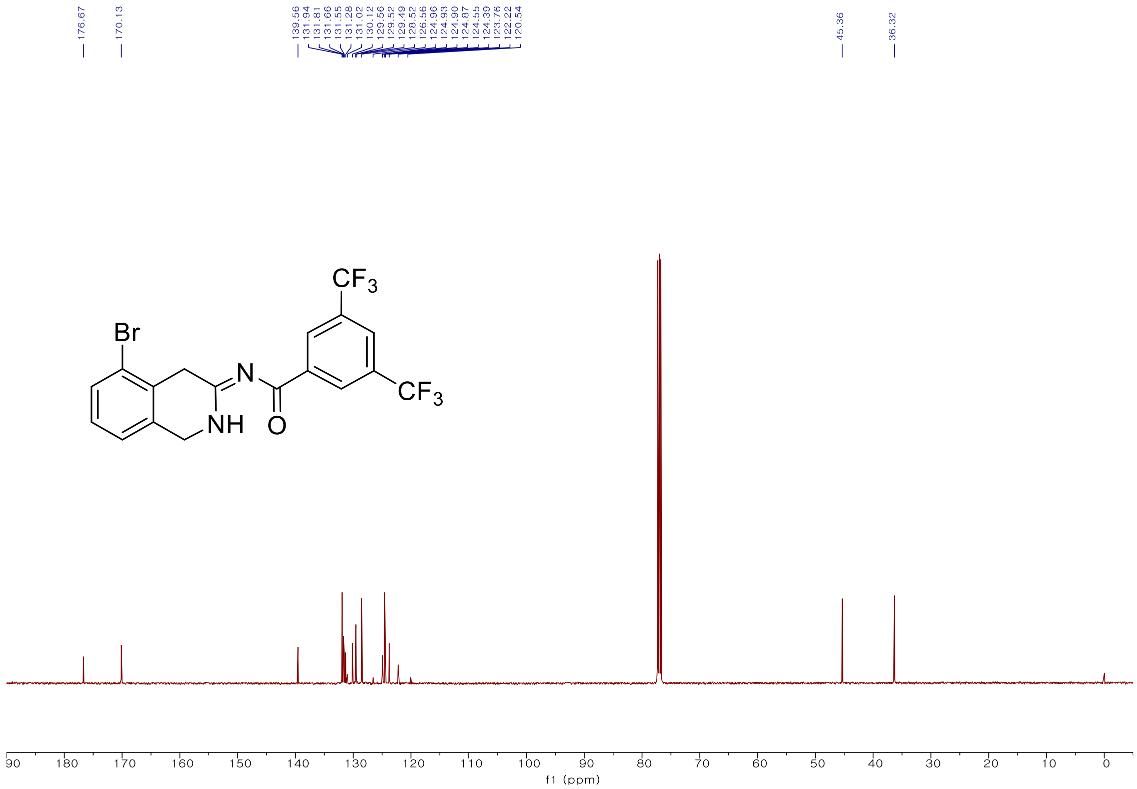

(*Z*)-*N*-(7-Bromo-1,4-dihydroisoquinolin-3(2*H*)-ylidene)-3,5-bis(trifluoromethyl)benzamide (Scheme 3, 4m)

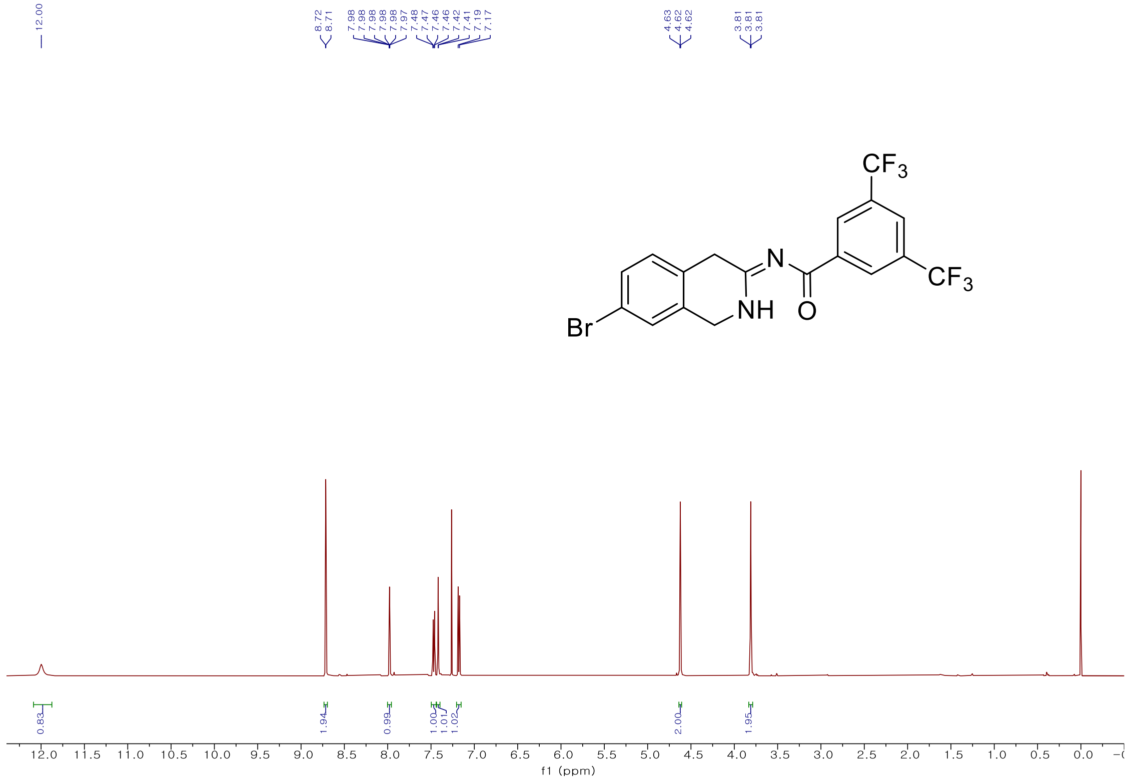

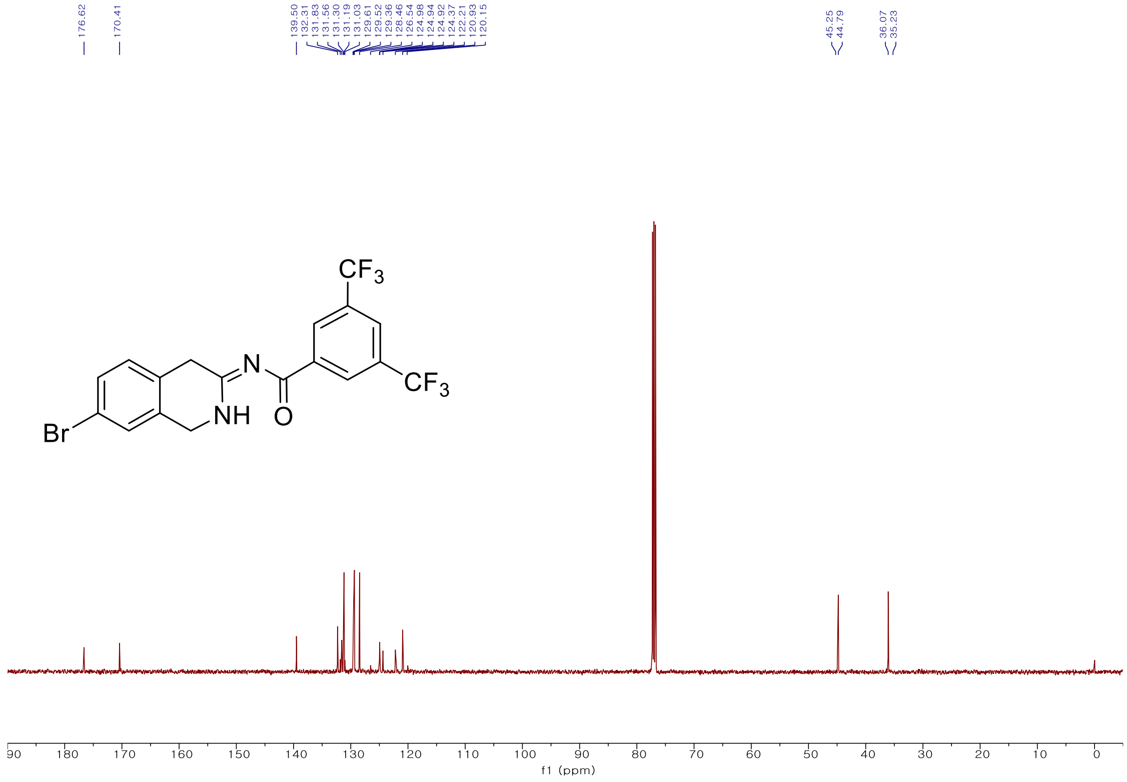

(*Z*)-3,5-bis(Trifluoromethyl)-*N*-(5-((trimethylsilyl)ethynyl)-1,4-dihydroisoquinolin-3(2*H*)-ylidene)benzamide (Scheme 3, 4n)

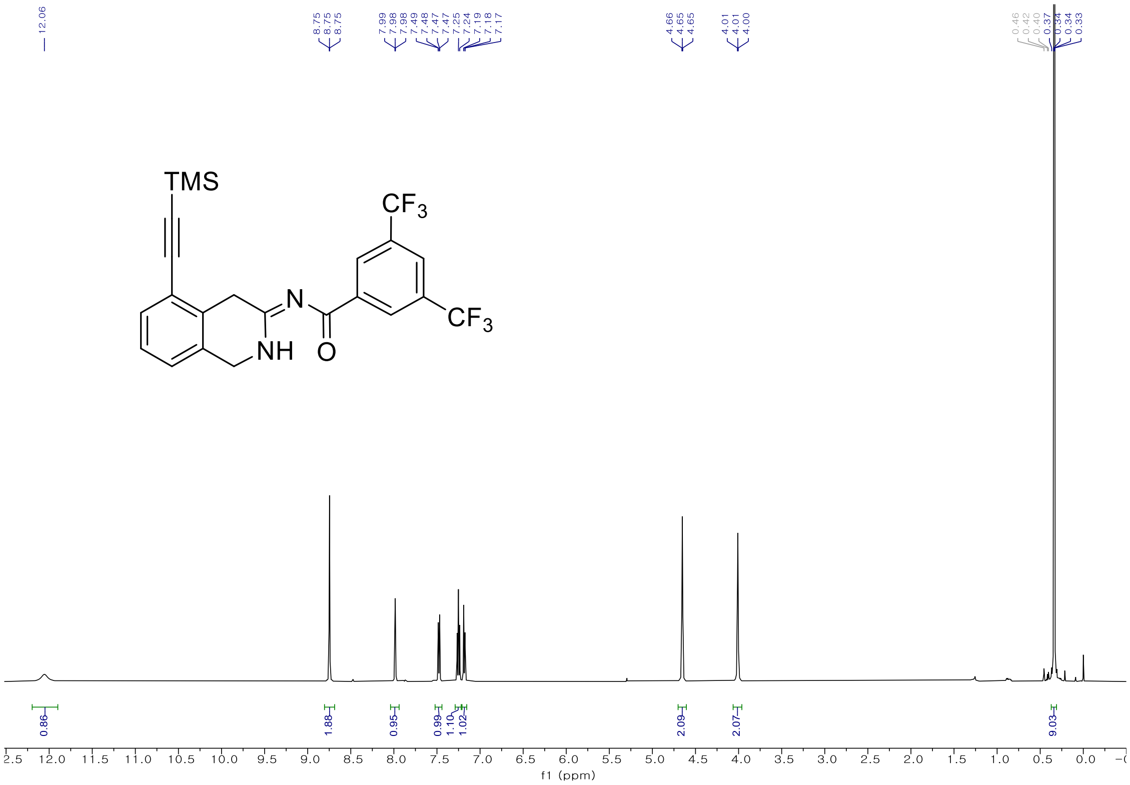

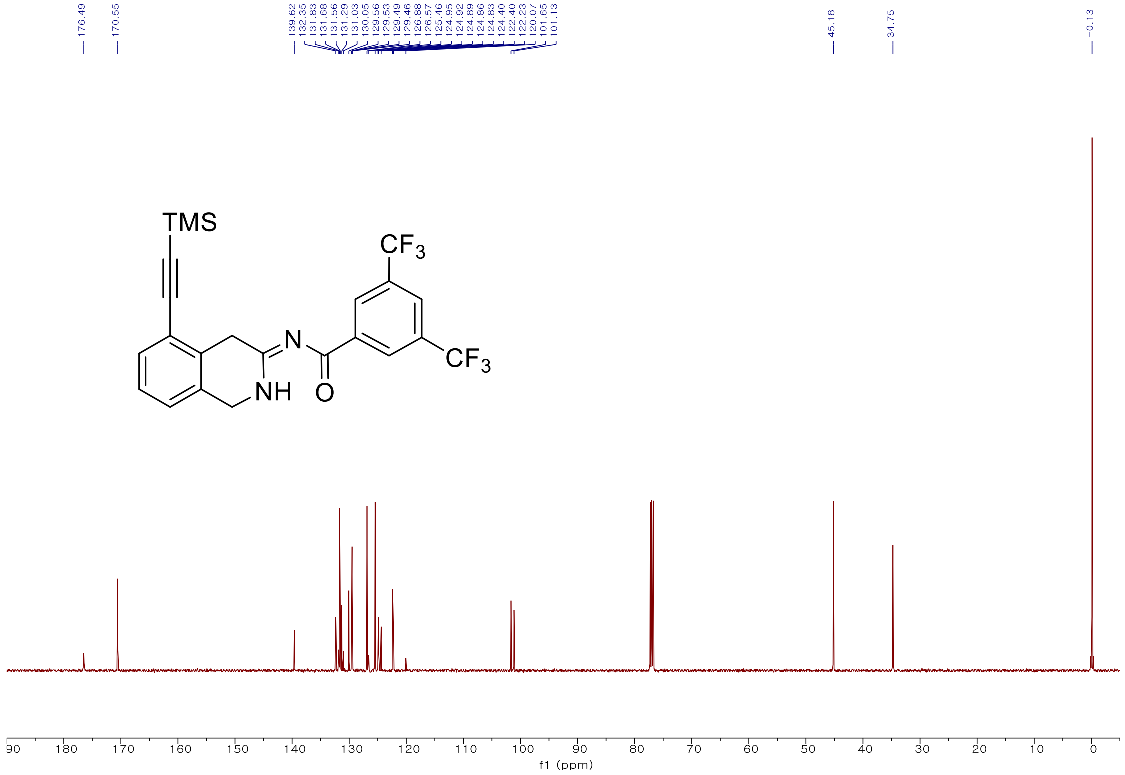

(*Z*)-3,5-bis(trifluoromethyl)-*N*-(5-((triisopropylsilyl)oxy)-1,4-dihydroisoquinolin-3(2*H*)-ylidene)benzamide (Scheme 3, 4o)

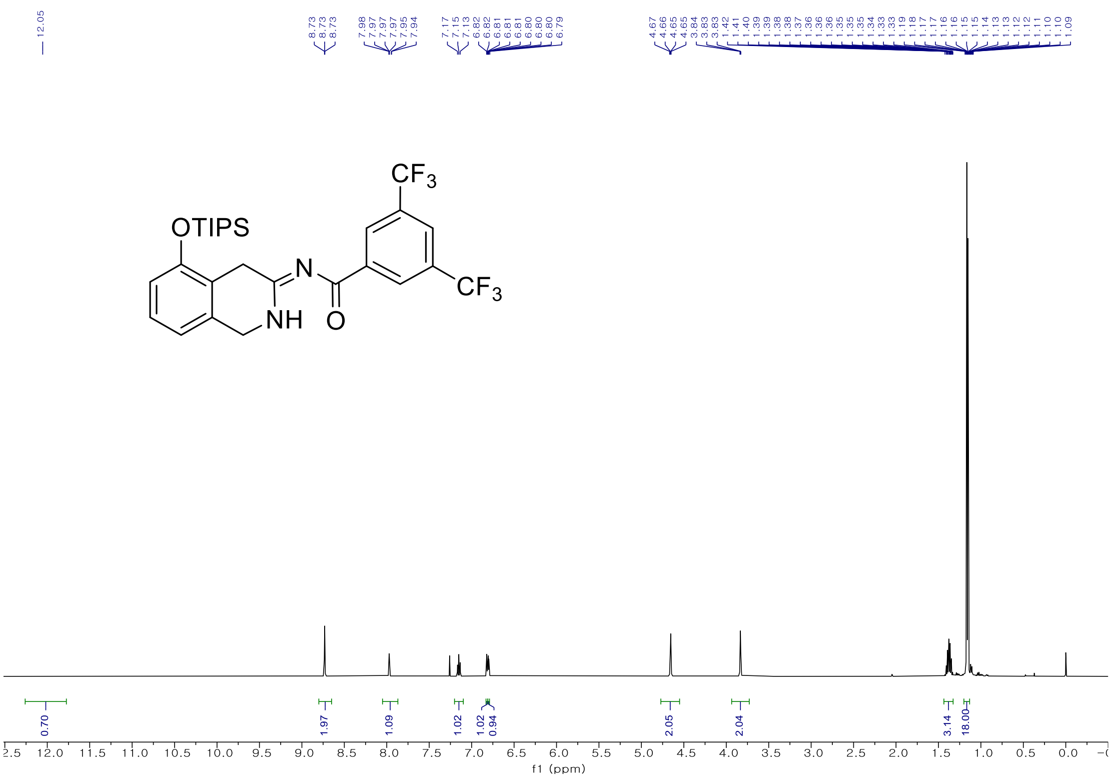

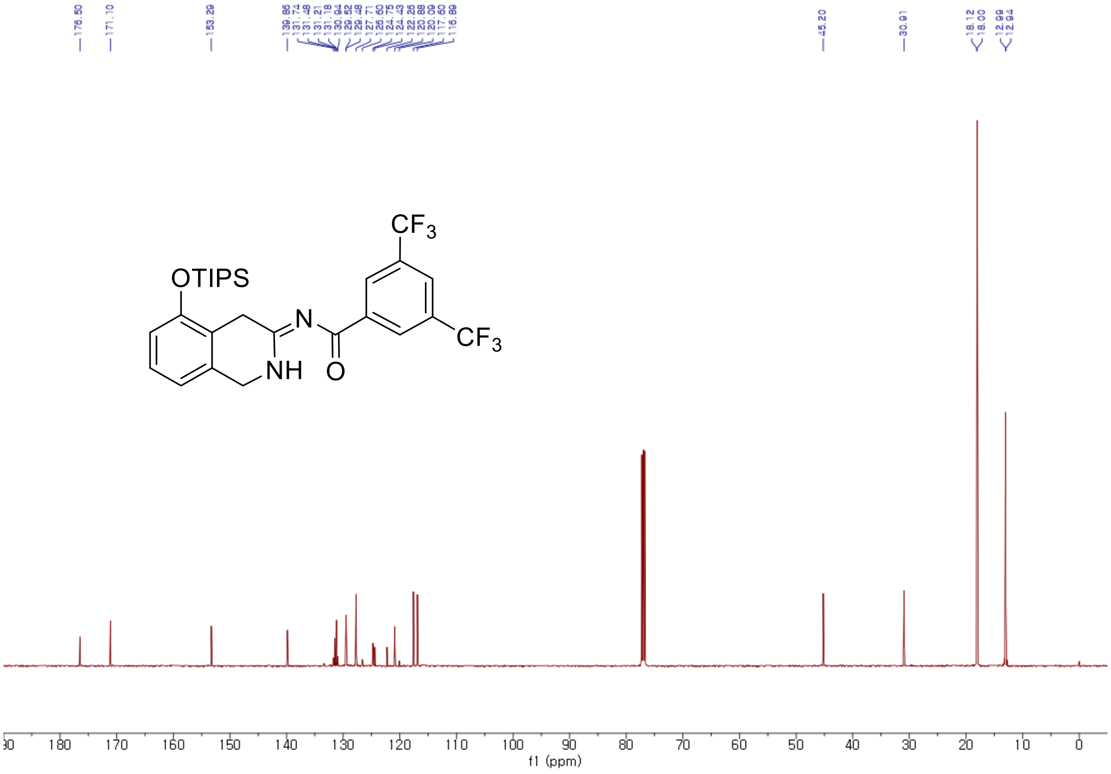

(*Z*)-3,5-Bis(trifluoromethyl)-*N*-(7-((triisopropylsilyl)oxy)-1,4-dihydroisoquinolin-3(2*H*)-ylidene)benzamide (Scheme 3, 4p)

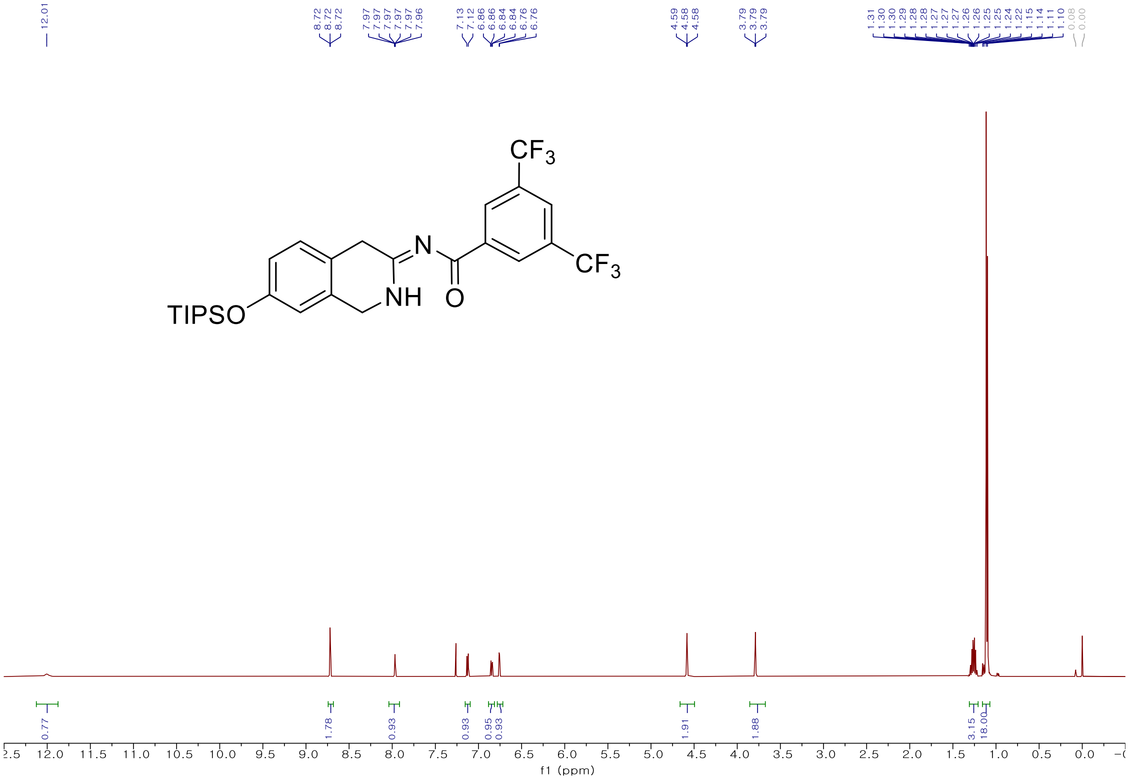

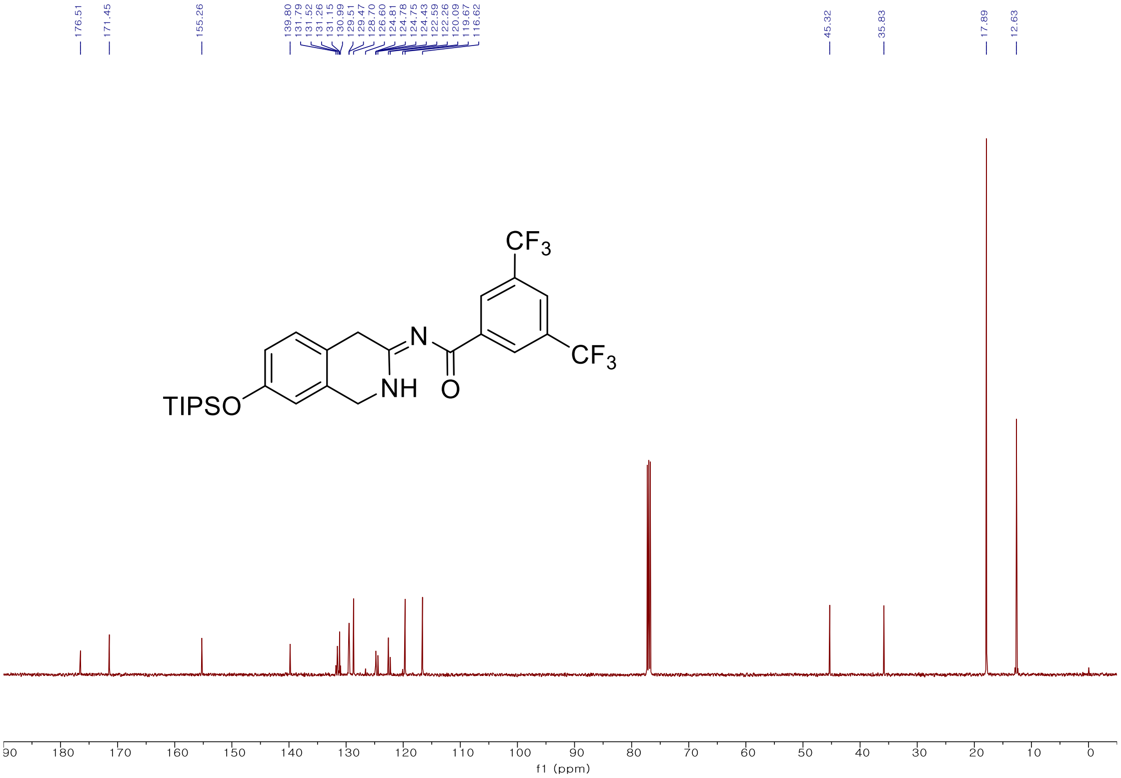

(*Z*)-*N*-(3,4-Dihydroquinolin-2(1*H*)-ylidene)benzamide (Scheme 4, 7a)

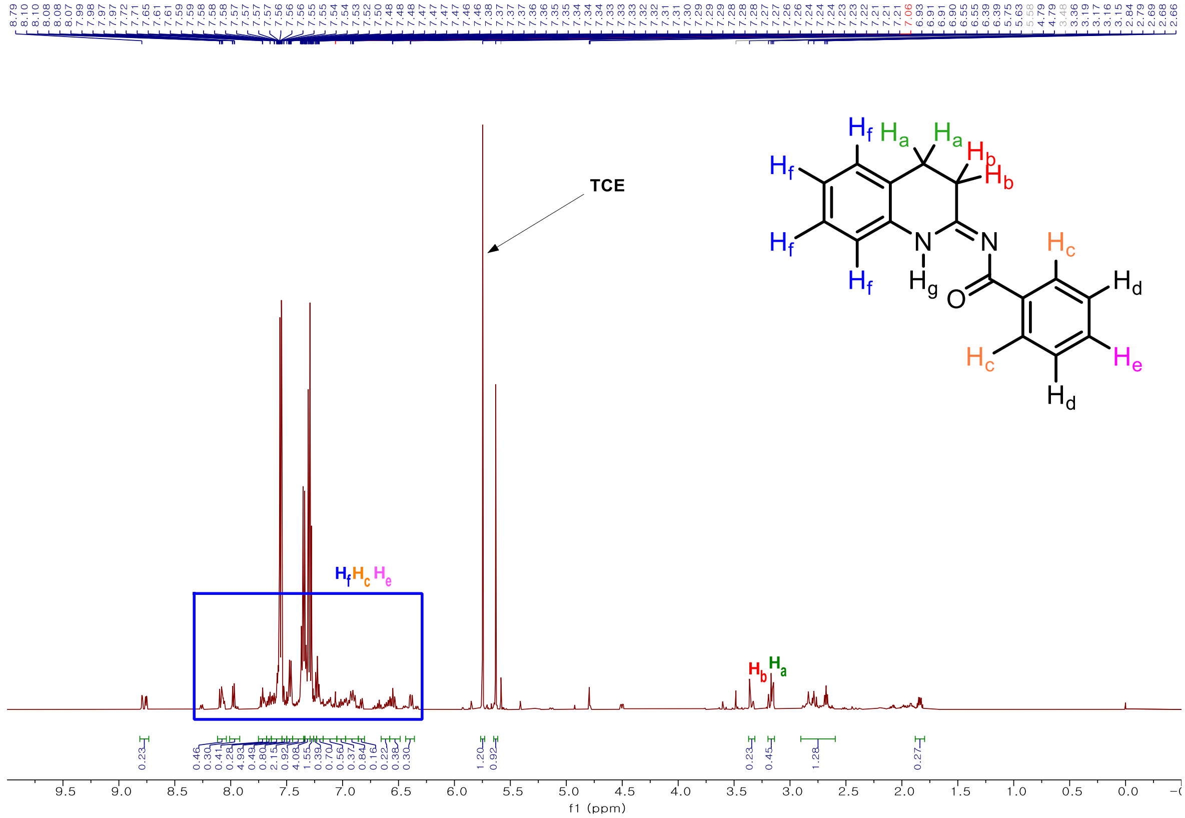

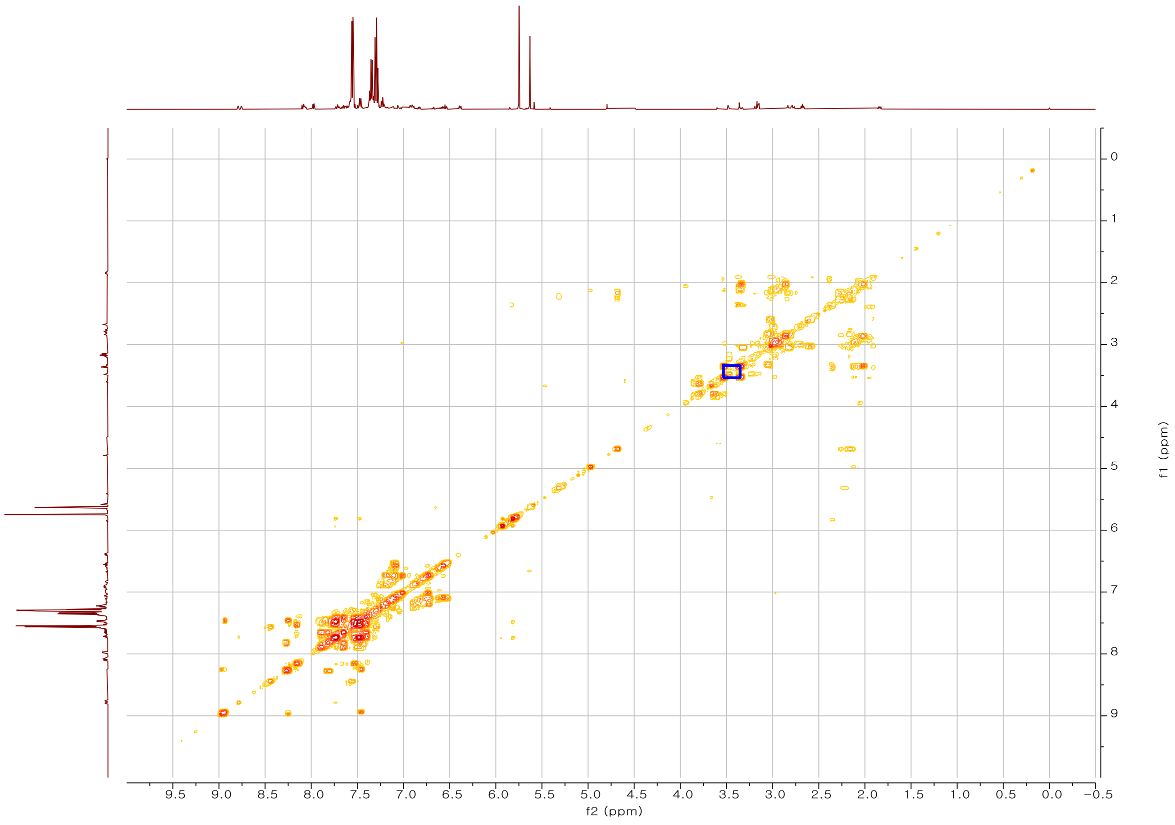

(*Z*)-4-Bromo-N-(3,4-dihydroquinolin-2(1*H*)-ylidene)benzamide (Scheme 4, 7b)

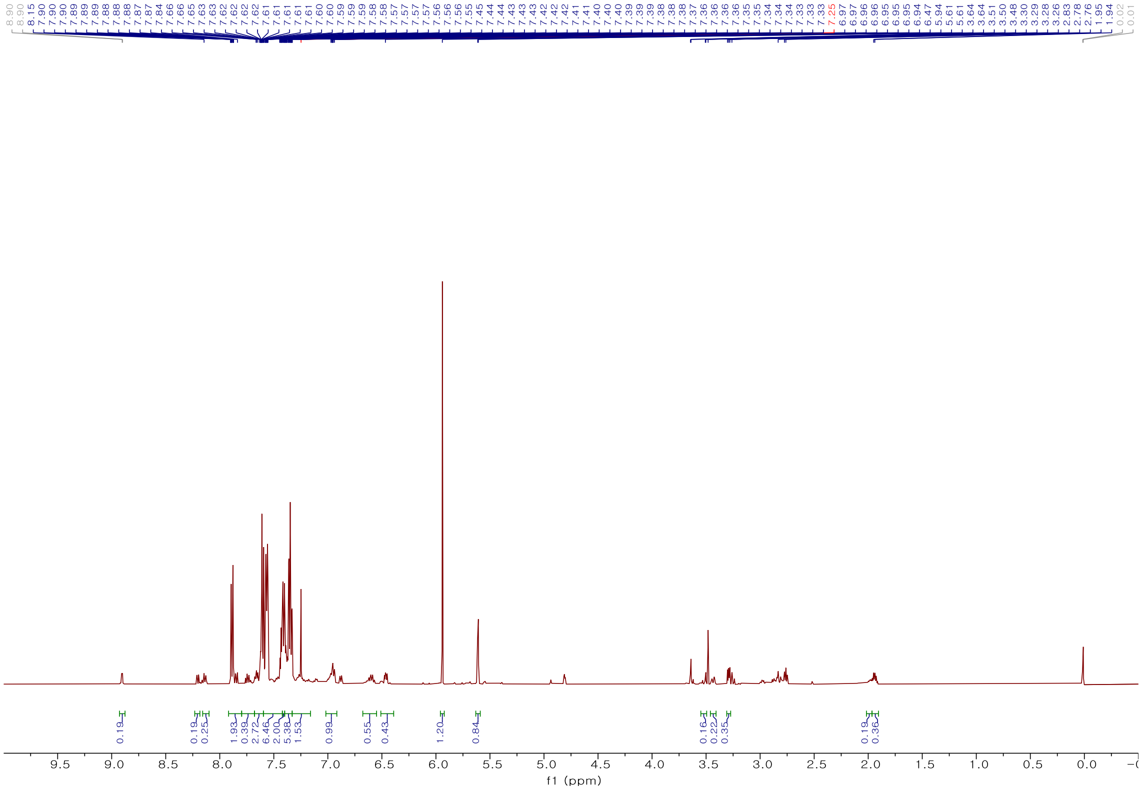

(*Z*)-4-Chloro-N-(3,4-dihydroquinolin-2(1*H*)-ylidene)benzamide (Scheme 4, 7c)

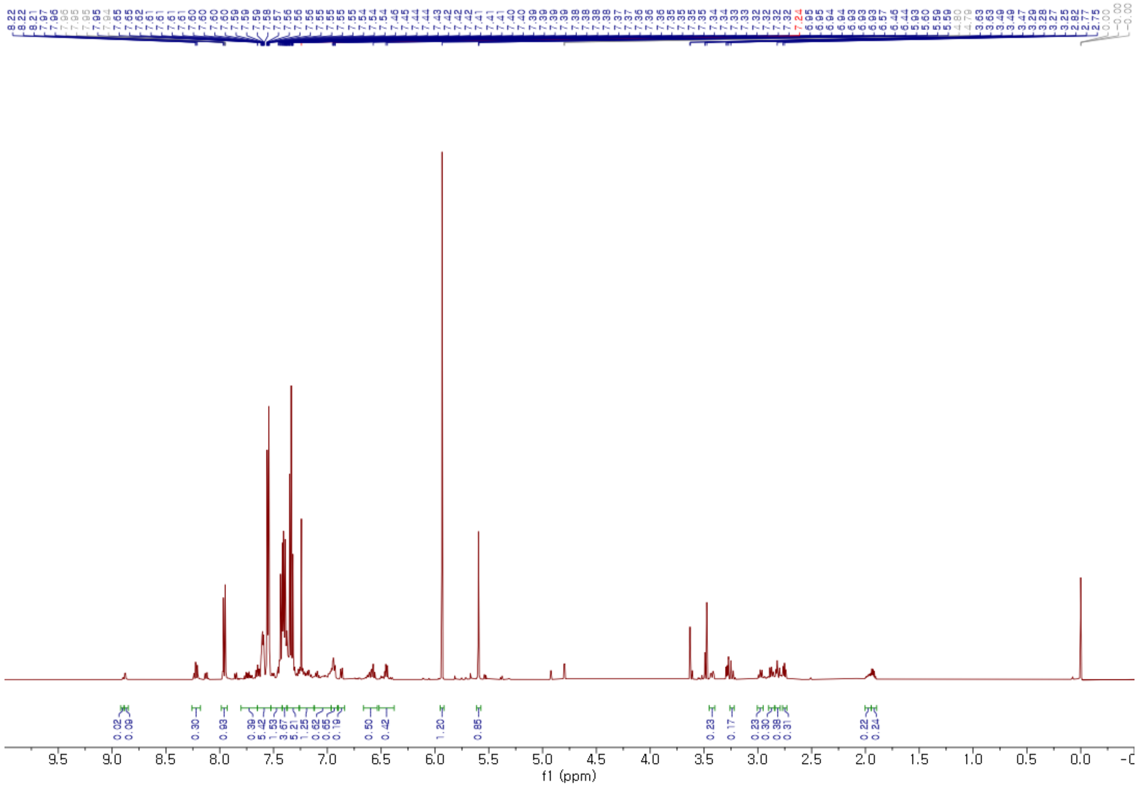

(*Z*)-*N*-(3,4-Dihydroquinolin-2(1*H*)-ylidene)-4-fluorobenzamide (Scheme 4, 7d)

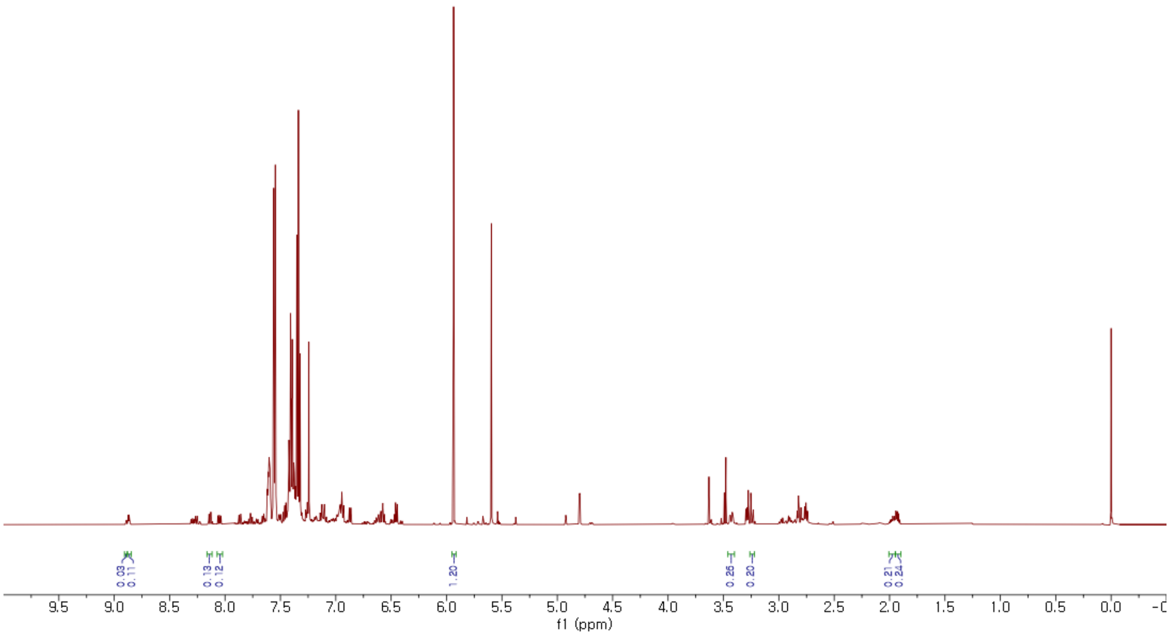

(*Z*)-*N*-(3,4-Dihydroquinolin-2(1*H*)-ylidene)-4-methoxybenzamide (Scheme 4, 7e)

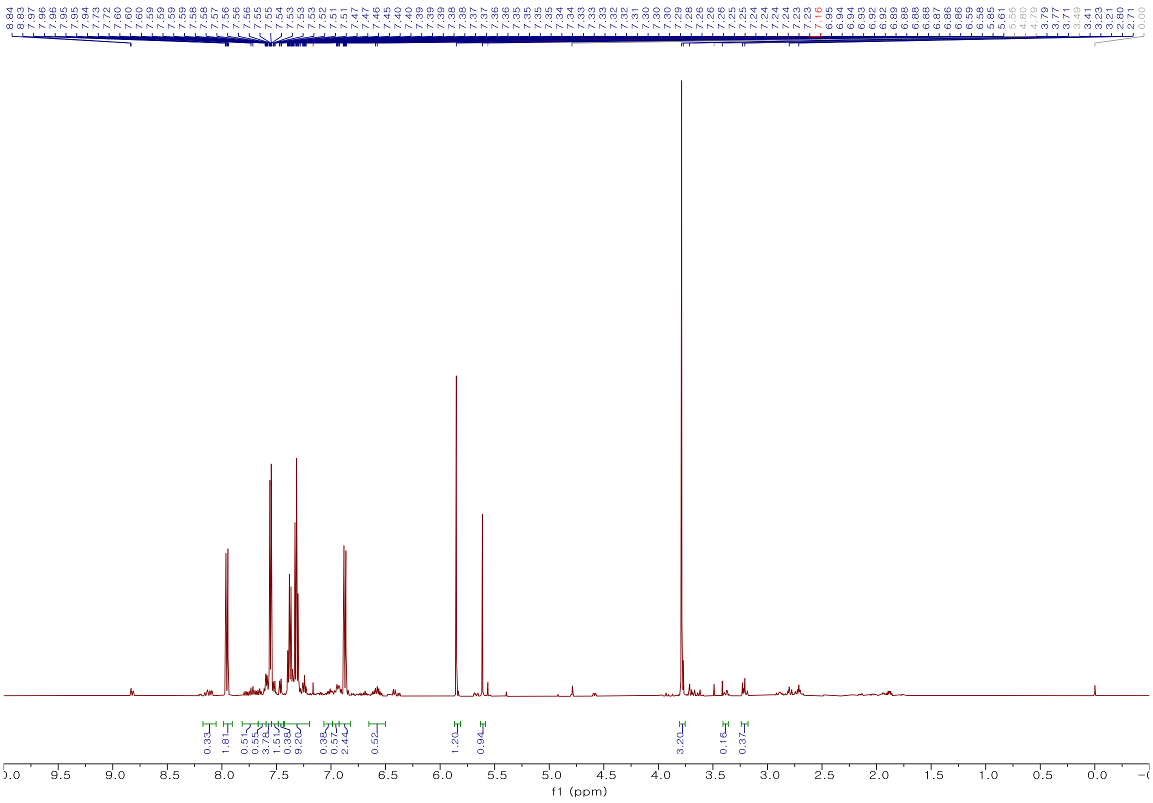

(*Z*)-*N*-(3,4-Dihydroquinolin-2(1*H*)-ylidene)-4-nitrobenzamide (Scheme 4, 7f)

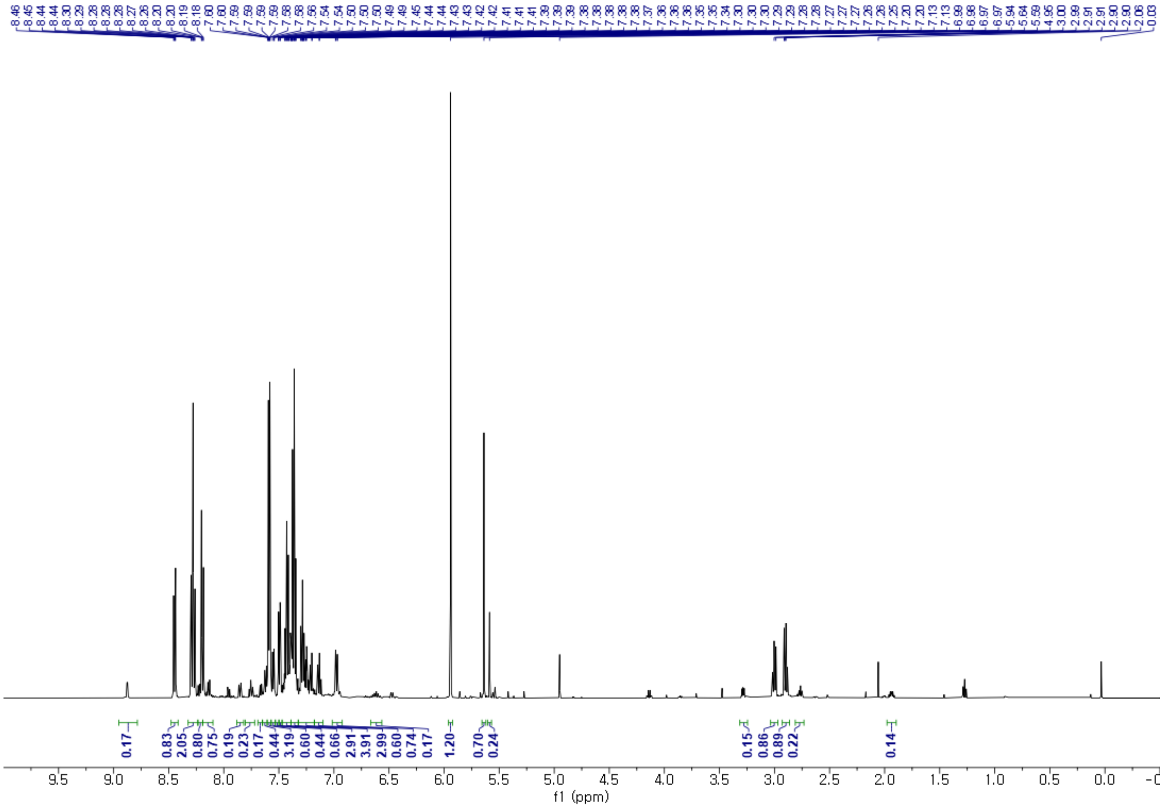

(*Z*)-*N*-(3,4-Dihydroquinolin-2(1*H*)-ylidene)-3,5-dinitrobenzamide (Scheme 4, 7g)

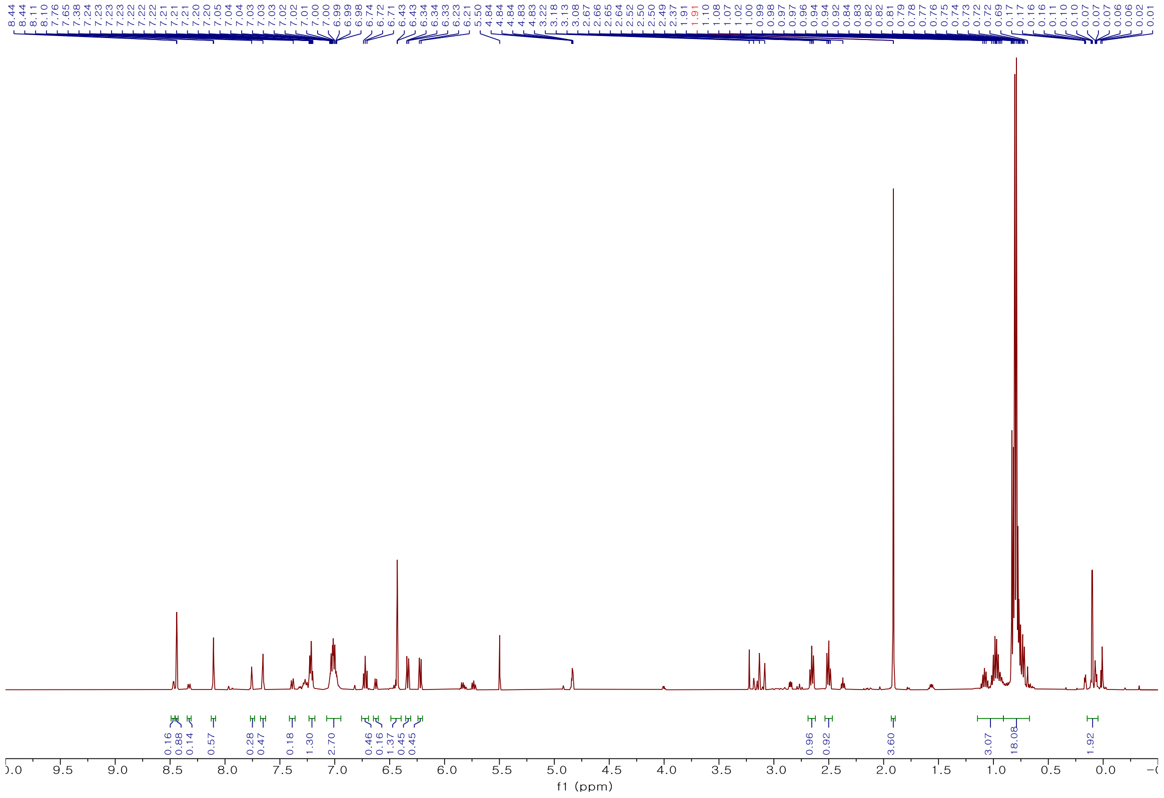

(*Z*)-*N*-(3,4-Dihydroquinolin-2(1*H*)-ylidene)-4-(trifluoromethyl)benzamide (Scheme 4, 7h)

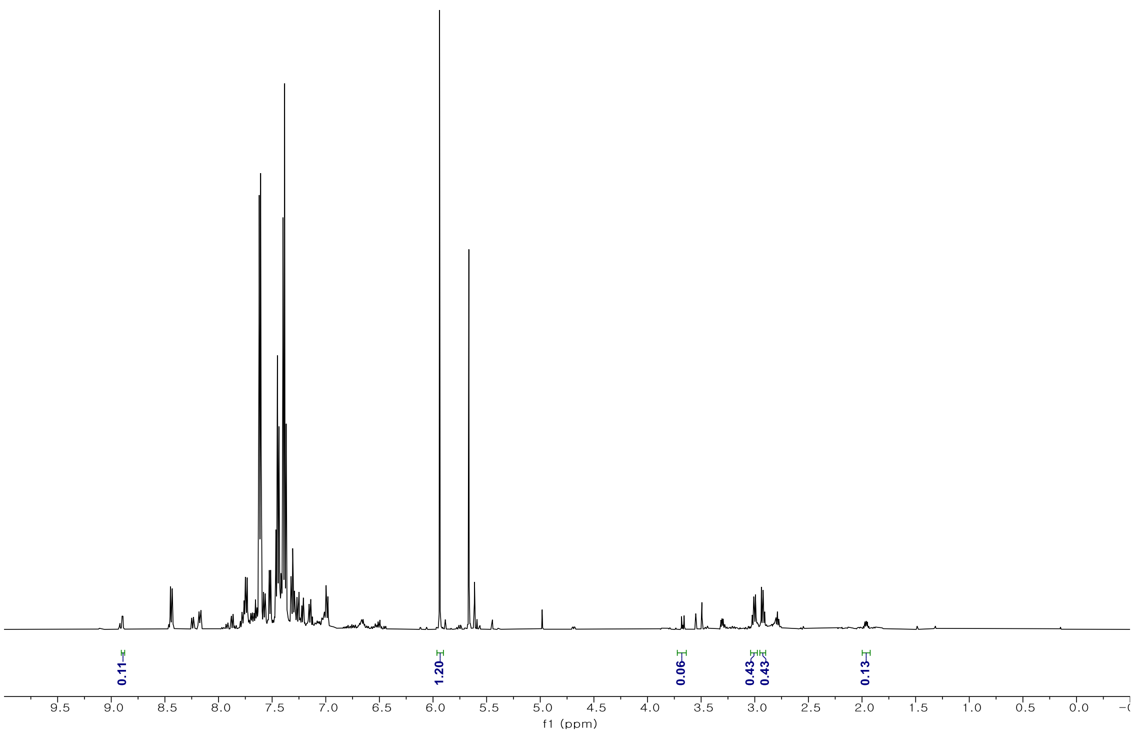

(*Z*)-*N*-(3,4-Dihydroquinolin-2(1*H*)-ylidene)-3,5-bis(trifluoromethyl)benzamide (Scheme 5, 7i)

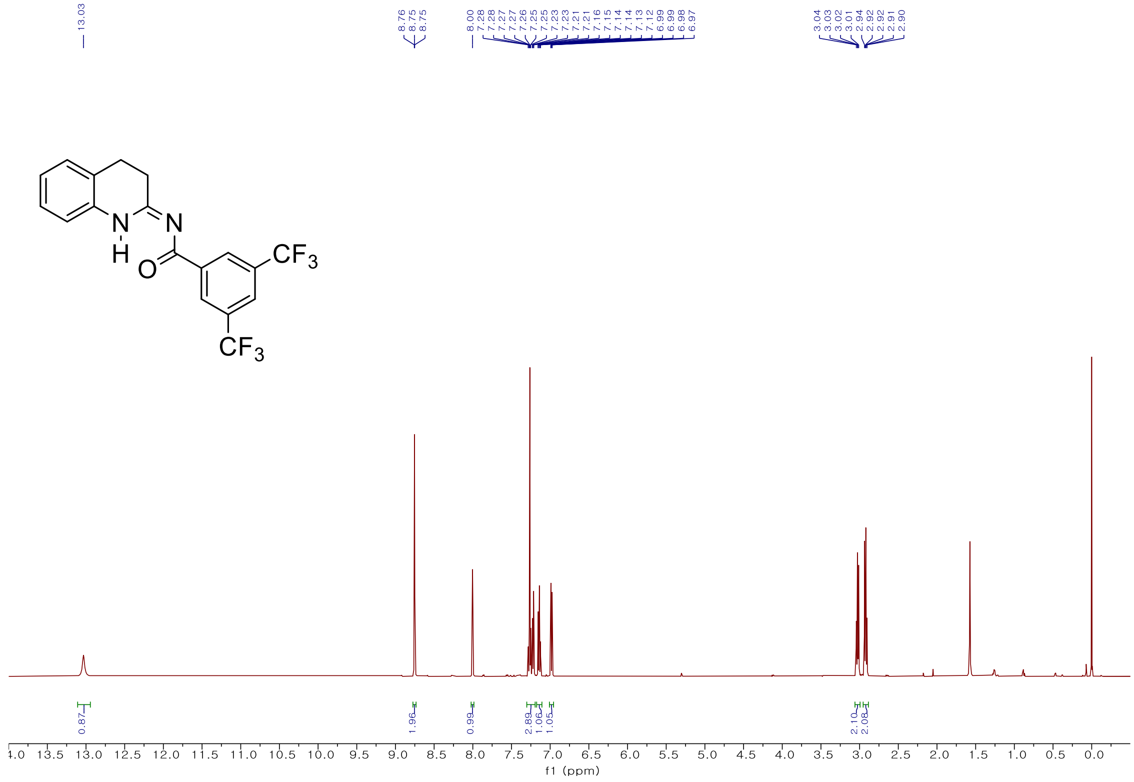

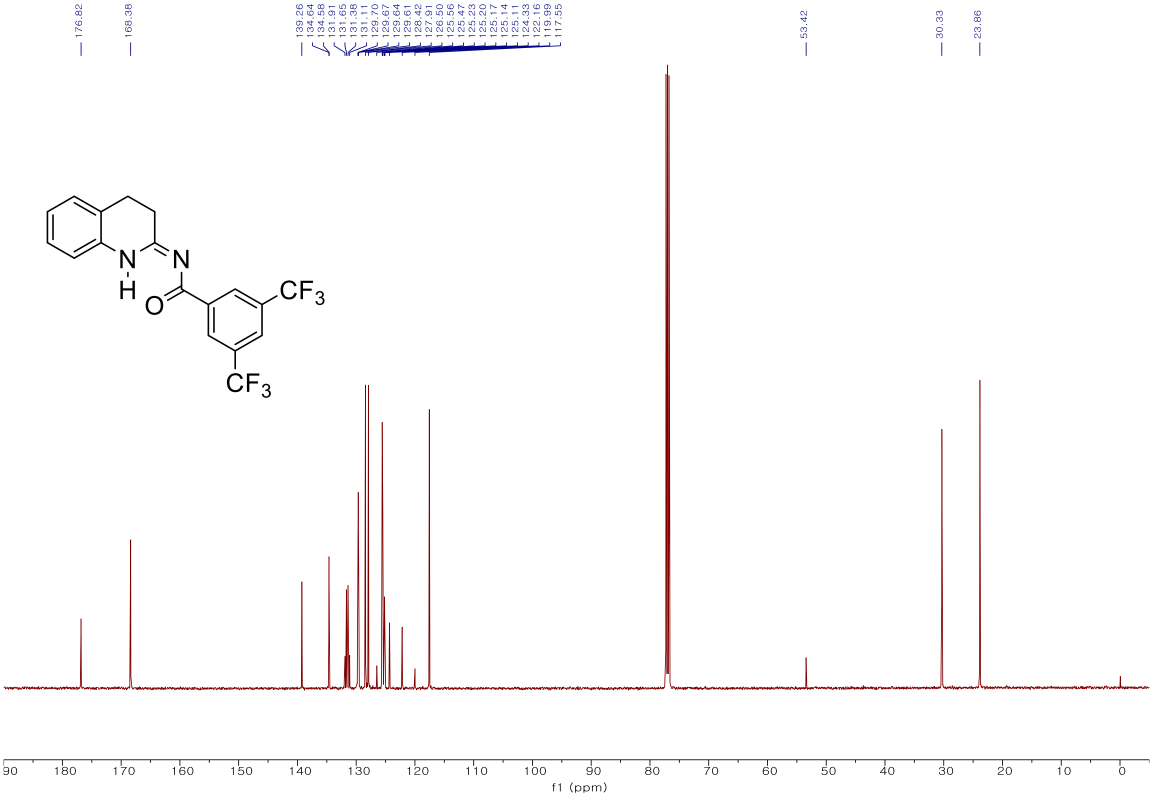

(*Z*)-*N*-(6-Methyl-3,4-dihydroquinolin-2(1*H*)-ylidene)-3,5-bis(trifluoromethyl)benzamide (Scheme 5, 7j)

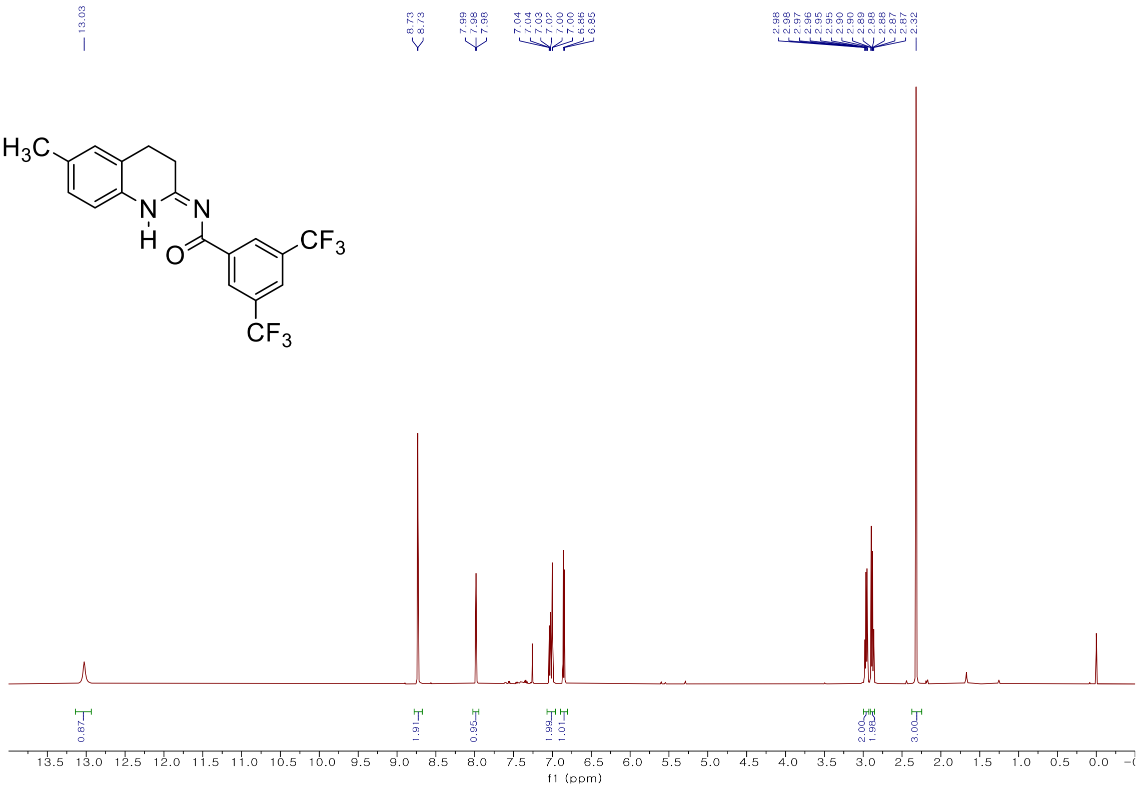

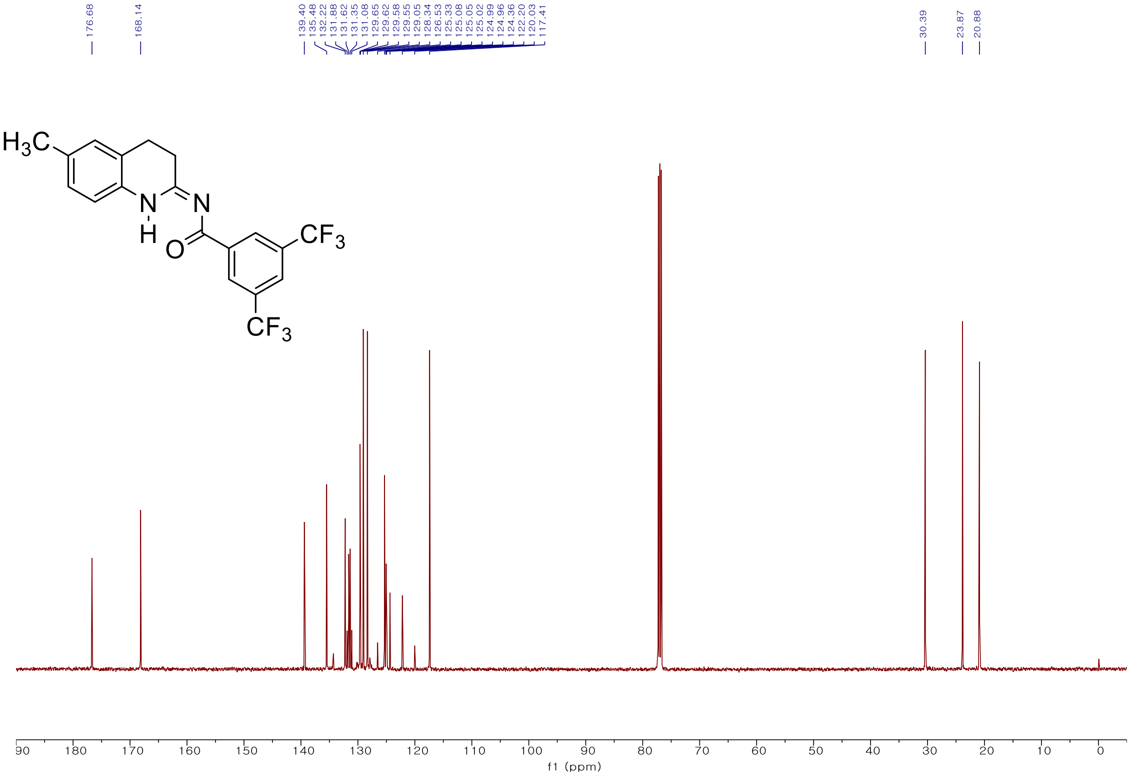

(*Z*)-3,5-Bis(trifluoromethyl)-*N*-(5-((triisopropylsilyl)oxy)-3,4-dihydroquinolin-2(1*H*)-ylidene)benzamide (Scheme 5, 7k)

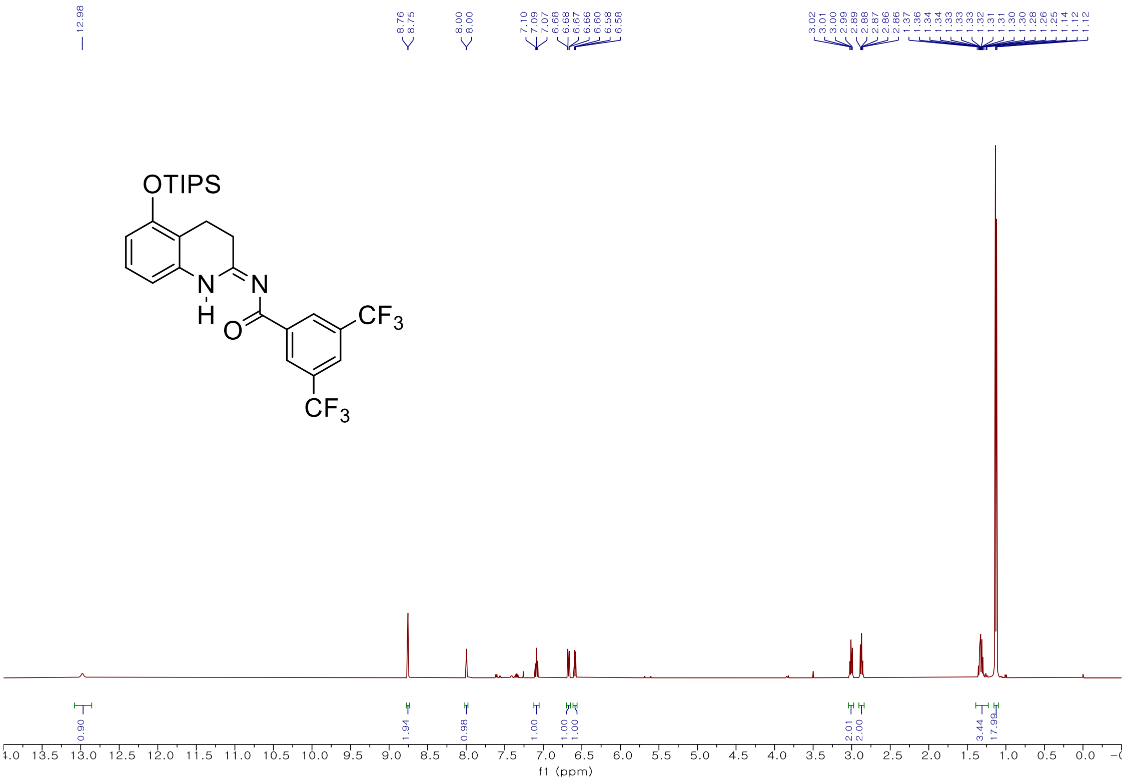

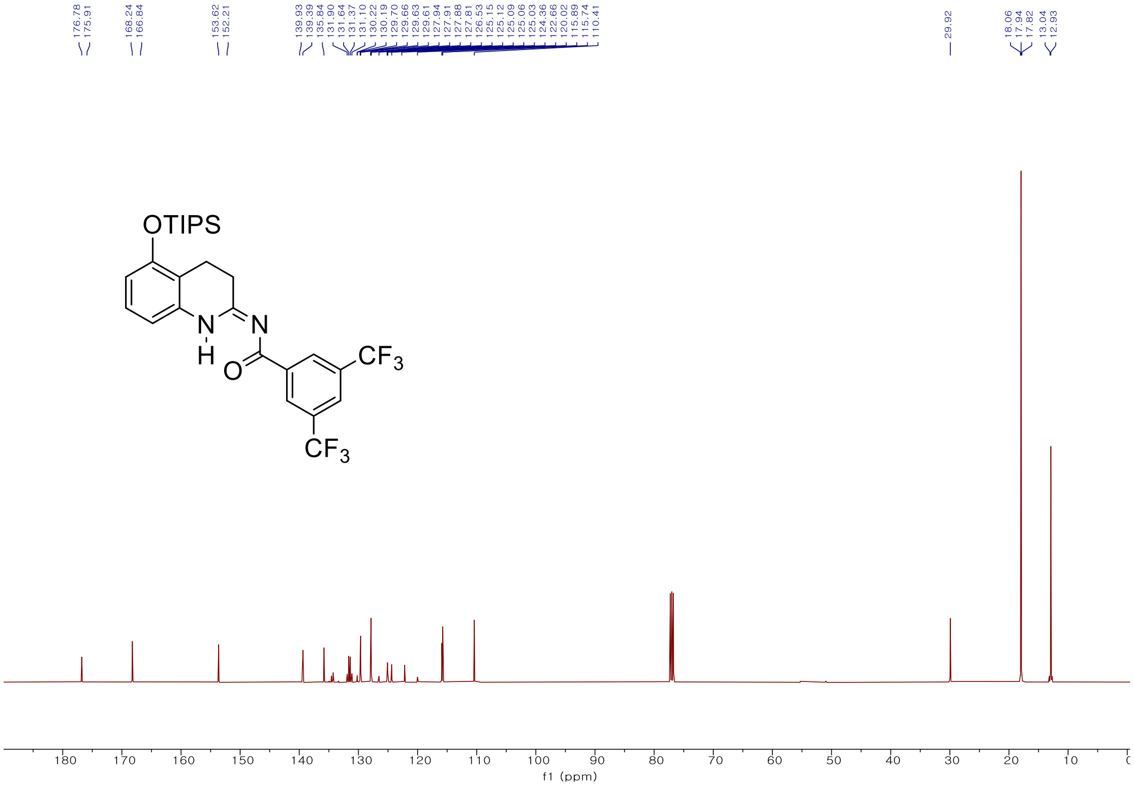

(*Z*)-3,5-Bis(trifluoromethyl)-*N*-(6-((triisopropylsilyl)oxy)-3,4-dihydroquinolin-2(1*H*)-ylidene)benzamide (Scheme 5, 7l)

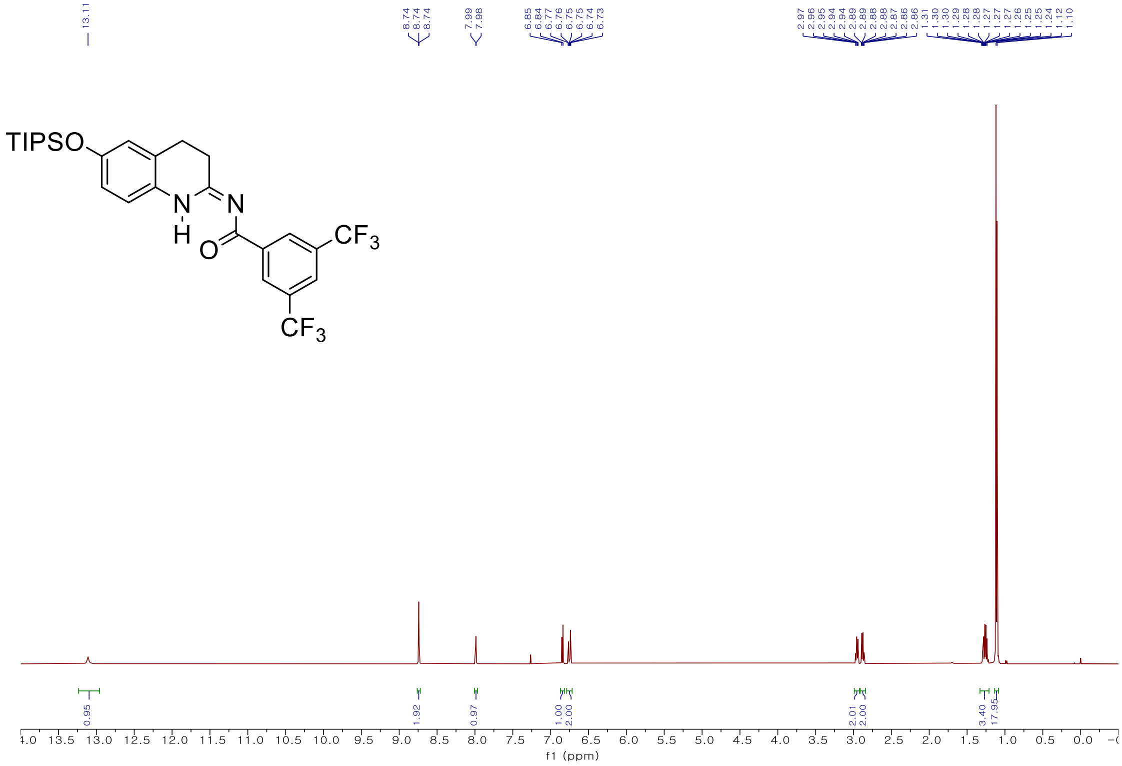

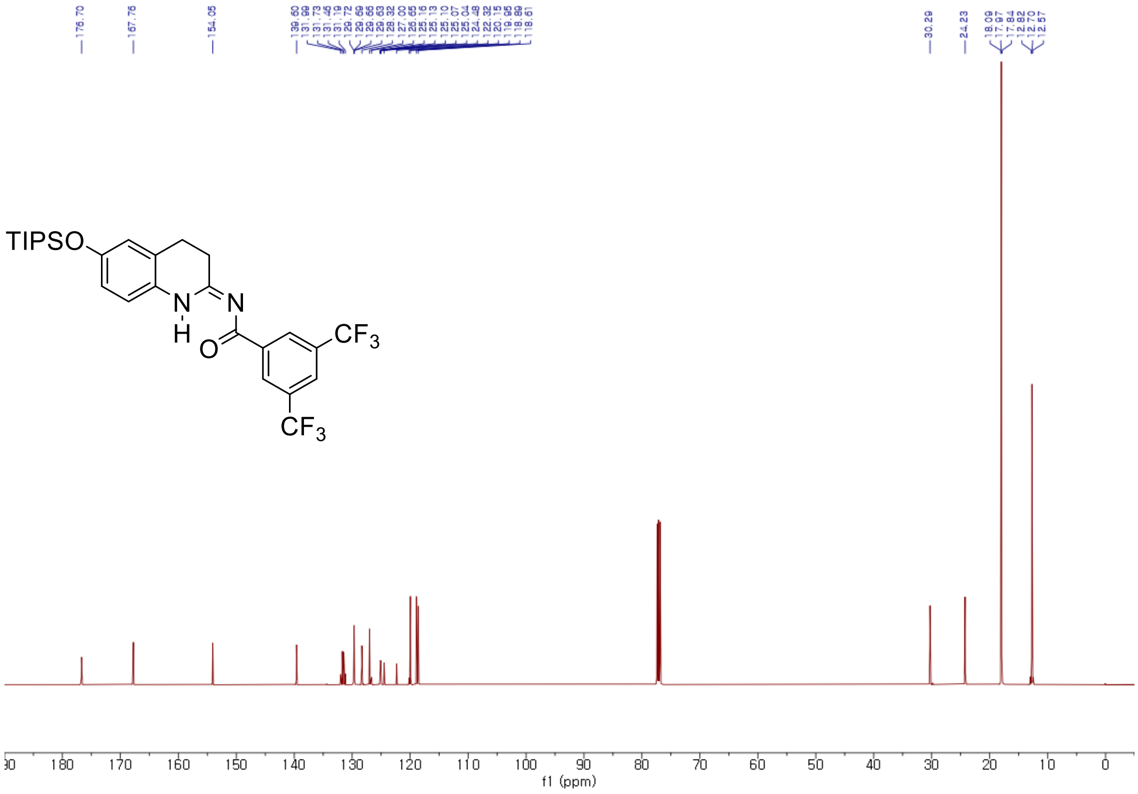

(*Z*)-*N*-(6-Methoxy-3,4-dihydroquinolin-2(1*H*)-ylidene)-3,5-bis(trifluoromethyl)benzamide (Scheme 5, 7m)

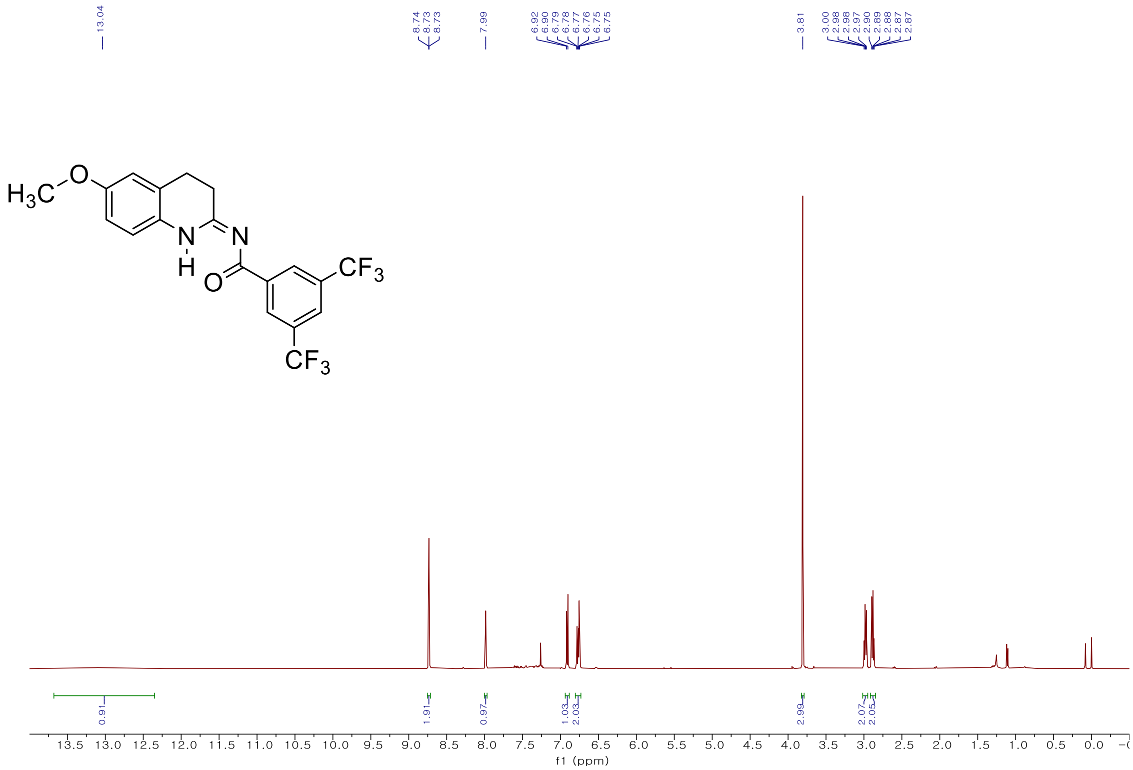

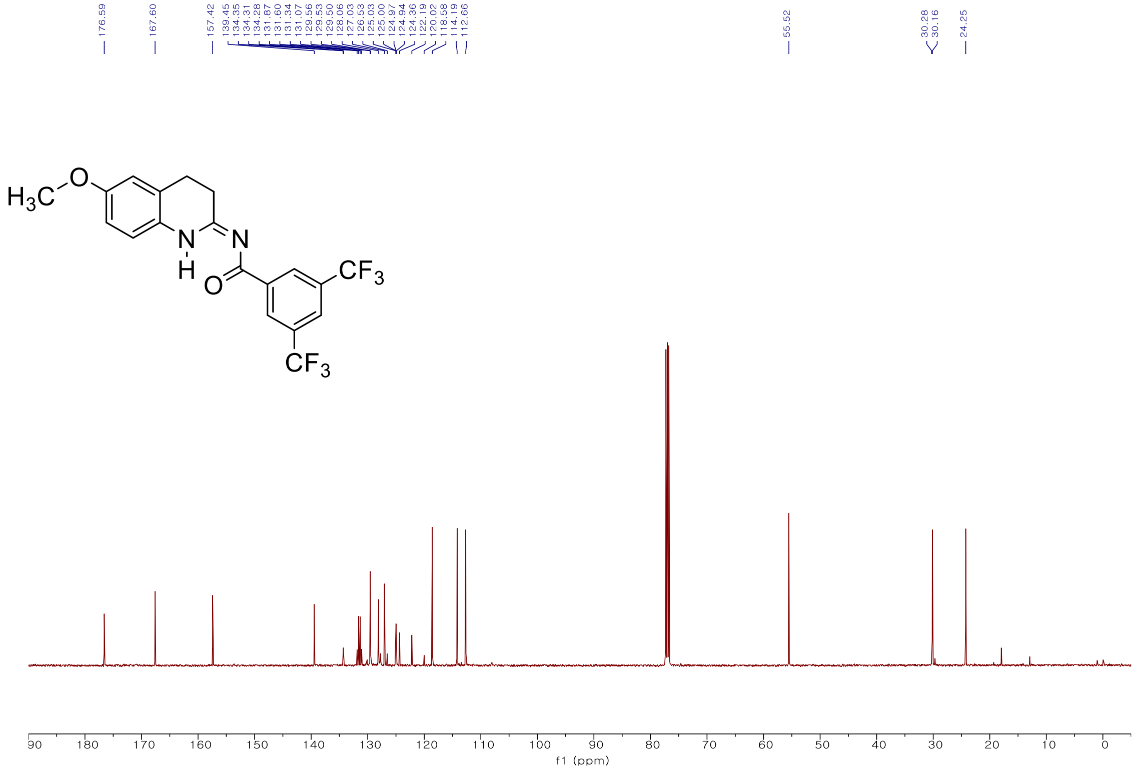

(*Z*)-*N*-(6-(Methoxymethoxy)-3,4-dihydroquinolin-2(1*H*)-ylidene)-3,5-bis(trifluoromethyl)benzamide (Scheme 5, 7n')

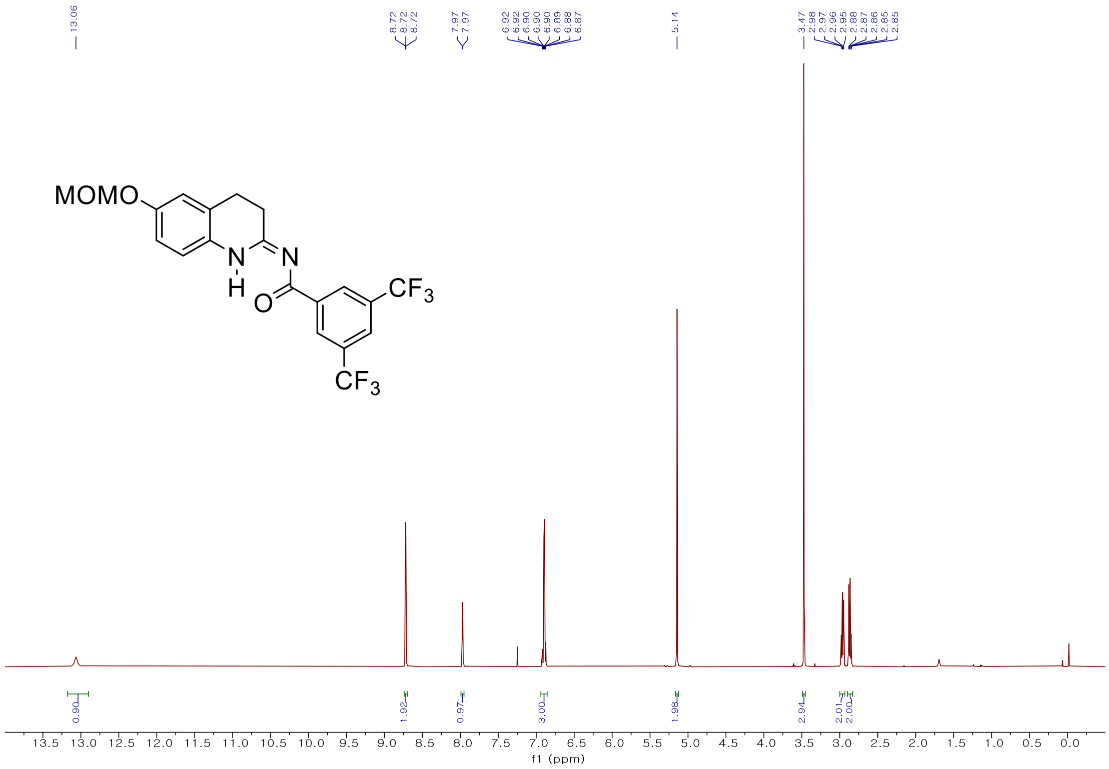

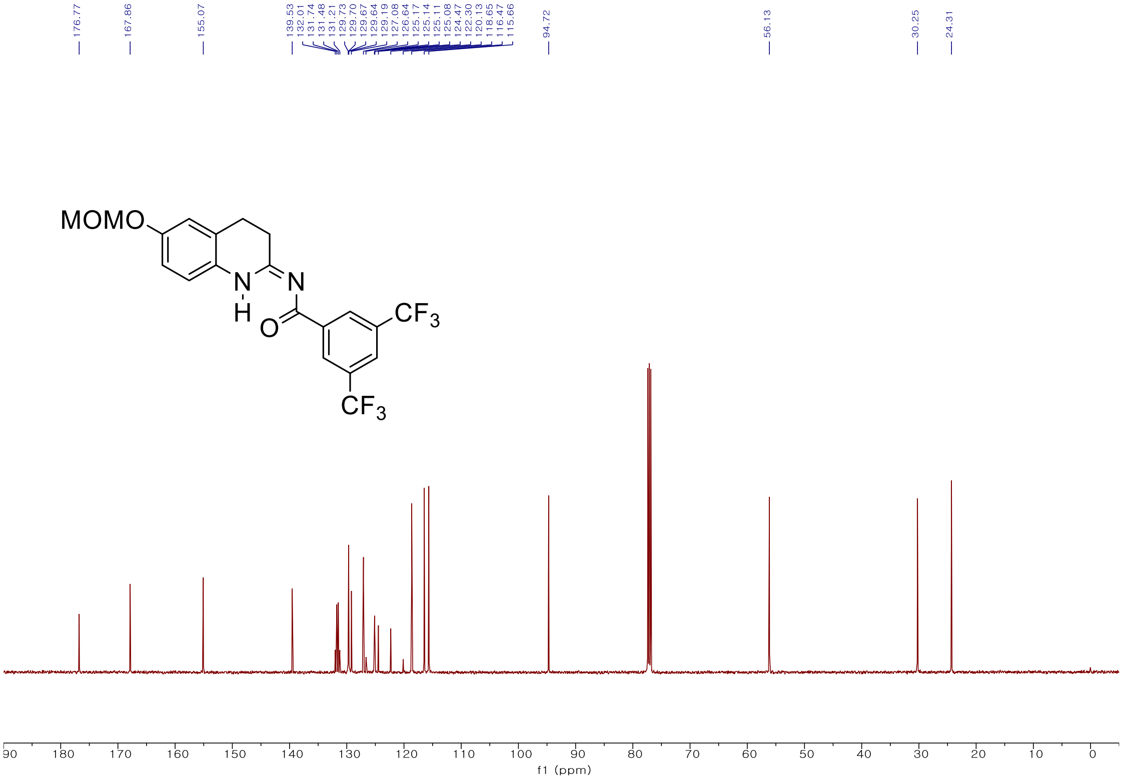

(*Z*)-*N*-(1,4-Dihydrobenzo[f]quinolin-3(2H)-ylidene)-3,5-bis(trifluoromethyl)benzamide (Scheme 5, 7o)

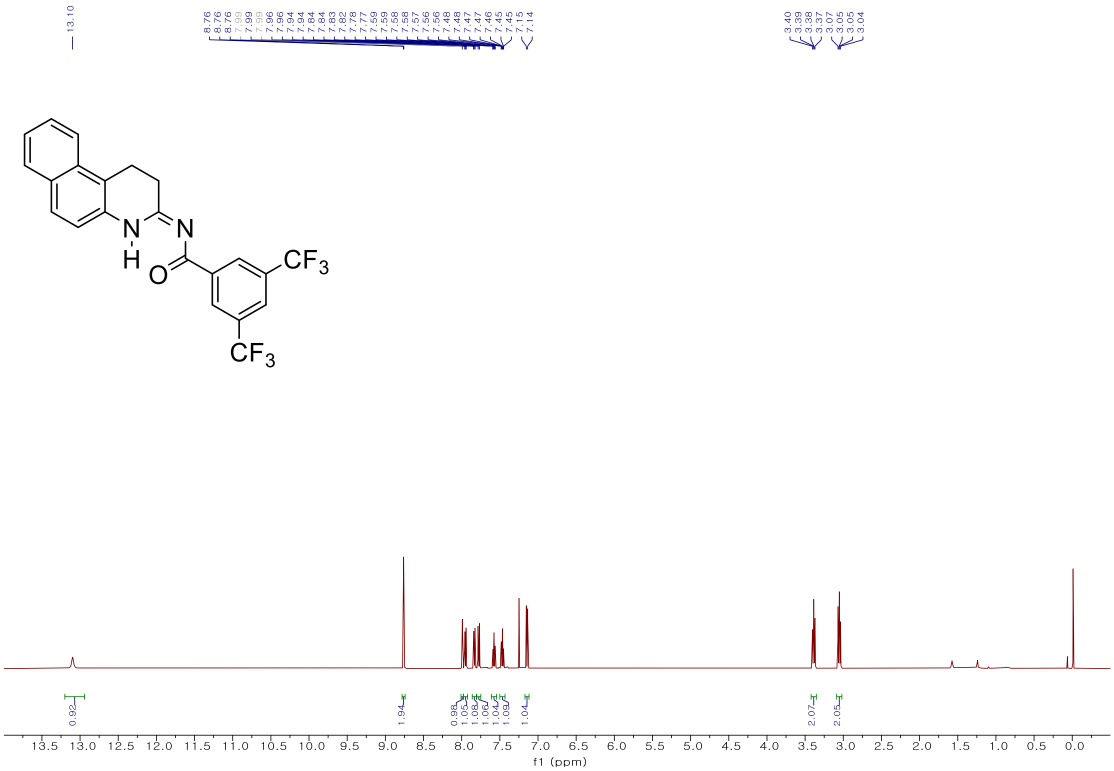

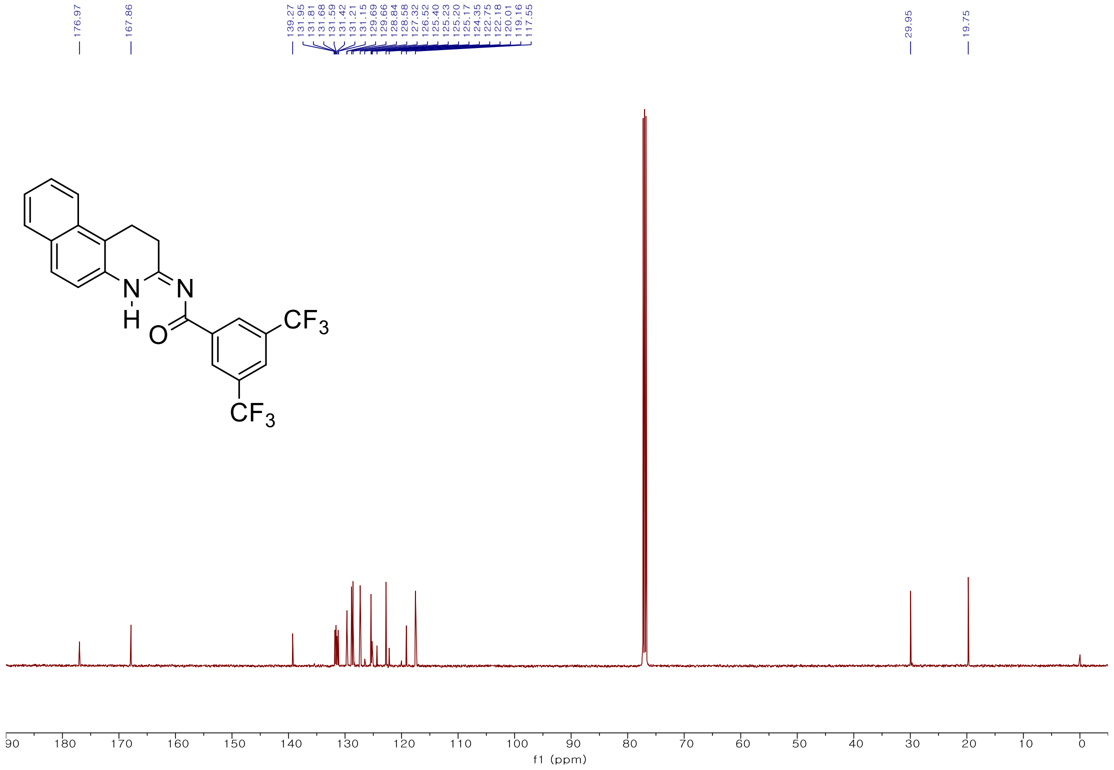

(*Z*)-*N*-(6-Phenyl-3,4-dihydroquinolin-2(1H)-ylidene)-3,5-bis(trifluoromethyl)benzamide (Scheme 5, 7p)

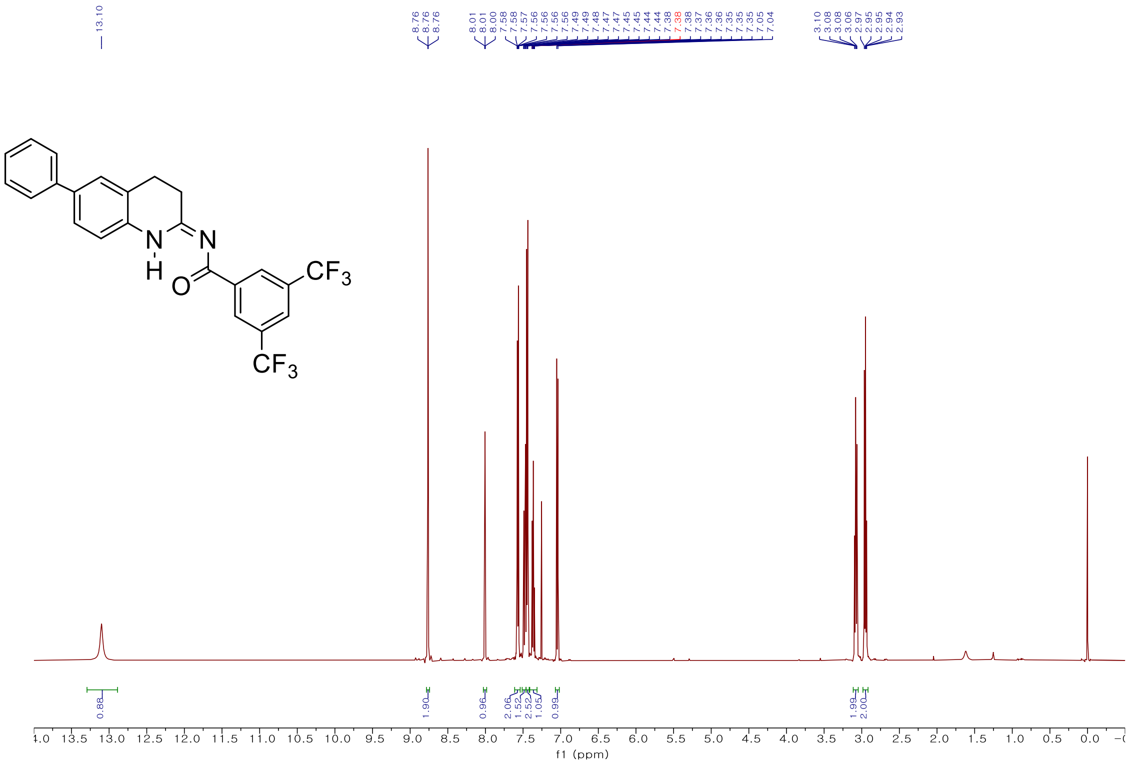

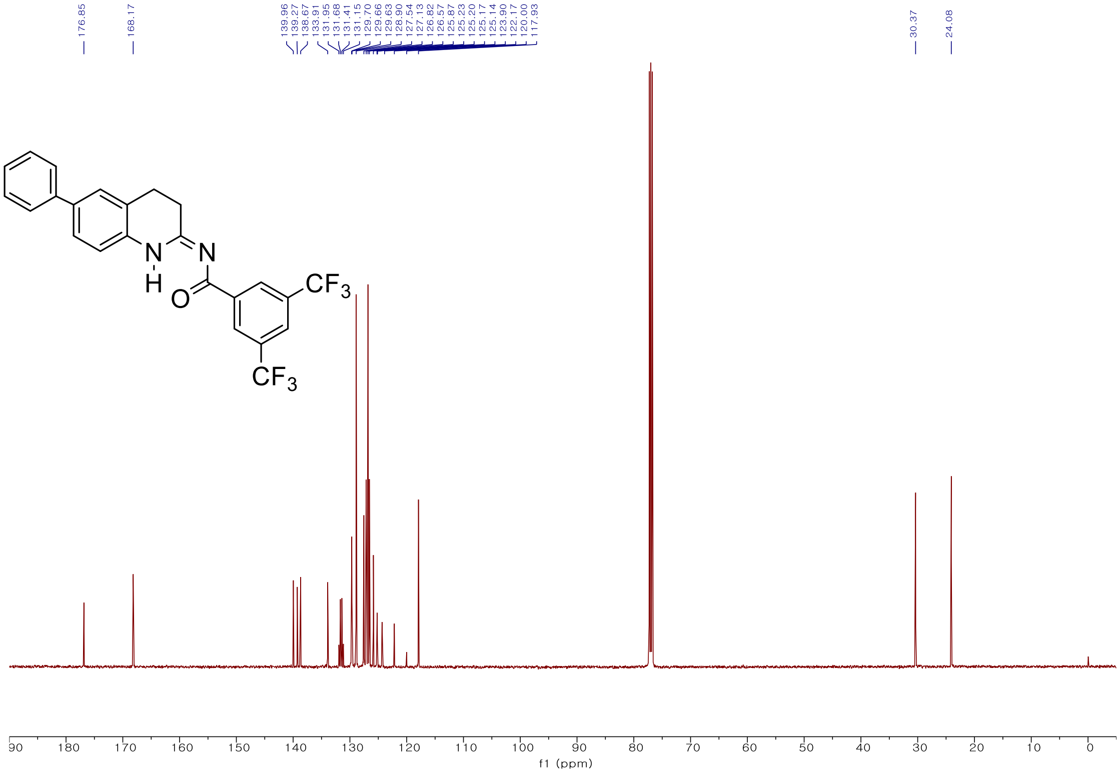

(*Z*)-*N*-(5-Bromo-3,4-dihydroquinolin-2(1*H*)-ylidene)-3,5-bis(trifluoromethyl)benzamide (Scheme 5, 7q)

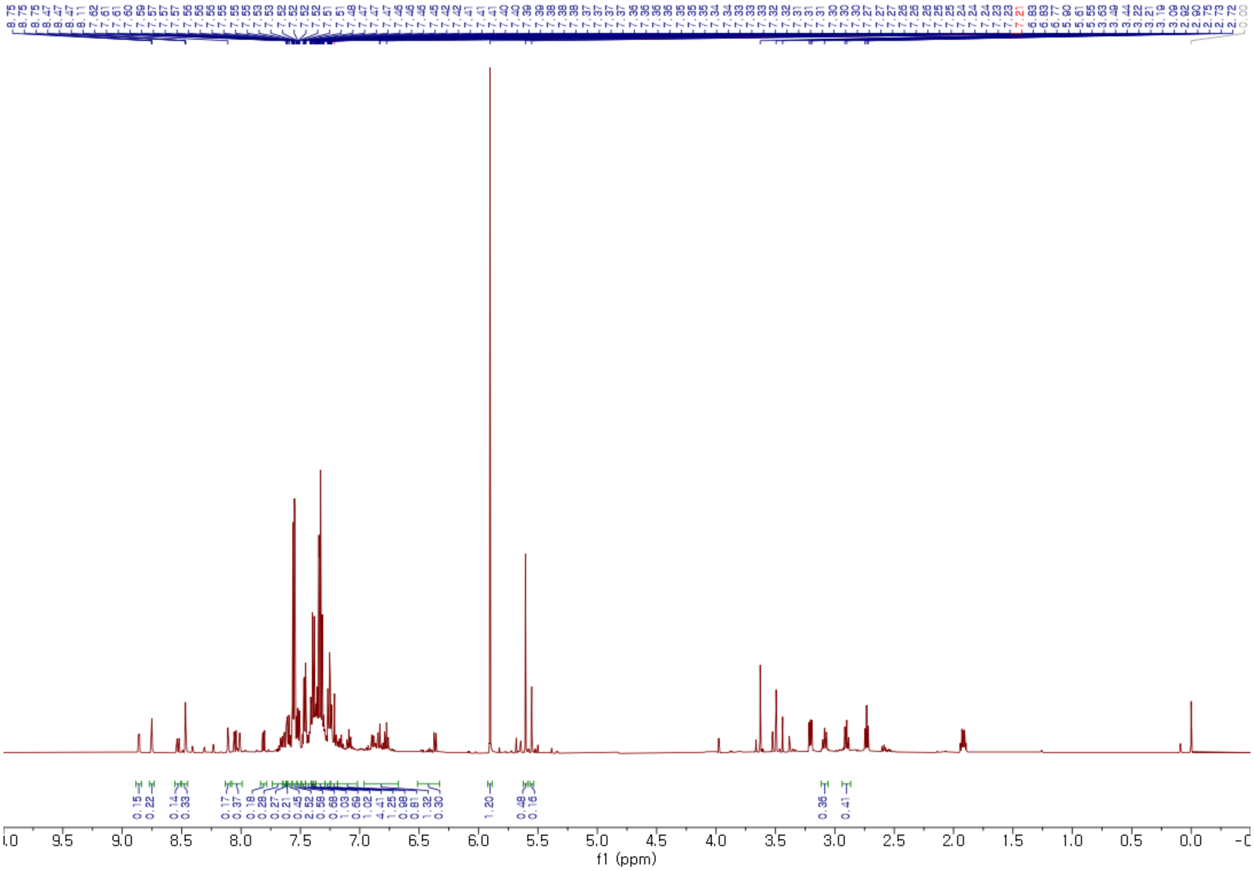

XI. Crystallographic Data

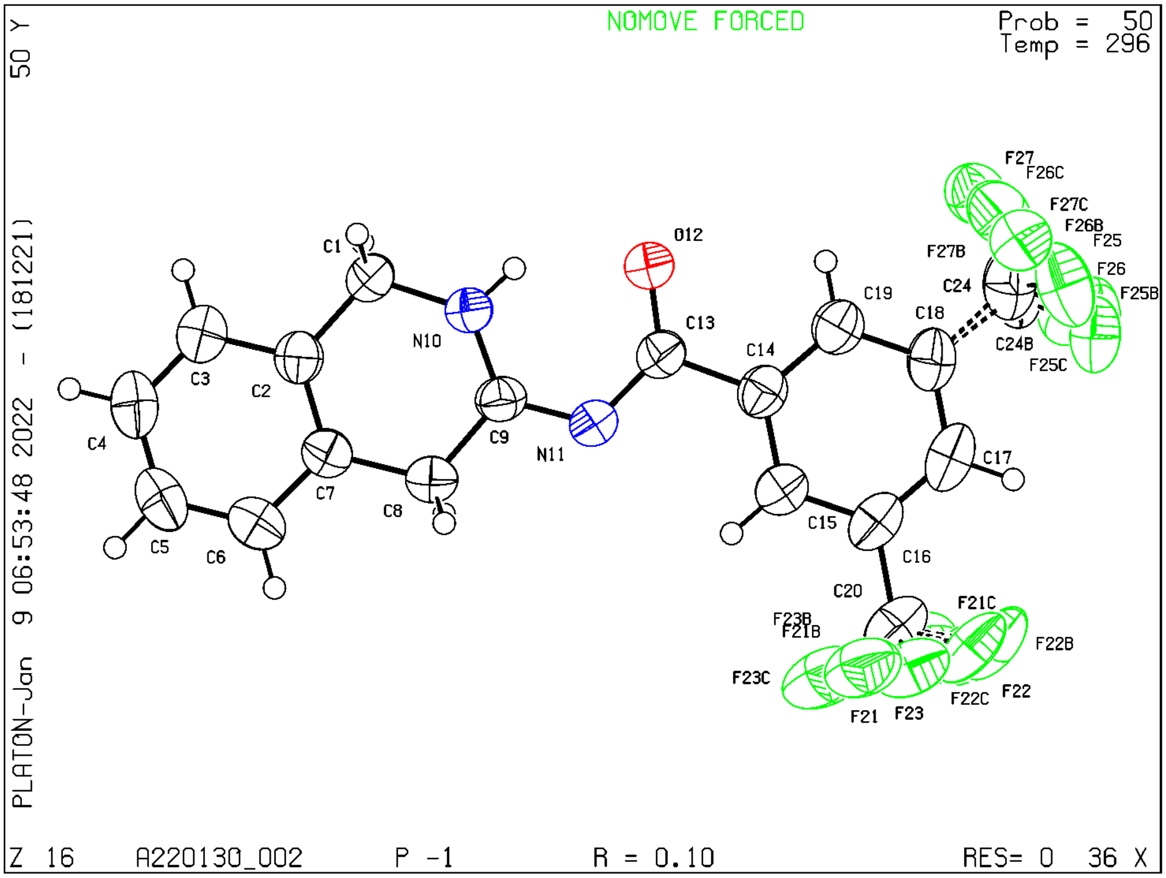

Figure S1. ORTEP illustration of **4i** with thermal ellipsoids drawn at 50% probability level.

Crystal data and structure refinement for **4i**.

Empirical formula	$C_{18} H_{12} F_6 N_2 O$	
Formula weight	386.30	
Temperature	296(2) K	
Wavelength	0.71073 Å	
Crystal system	Triclinic	
Space group	P -1	
Unit cell dimensions	a = 4.6672(16) Å	α = 69.968(9)°
	b = 13.241(4) Å	β = 83.993(10)°
	c = 14.417(5) Å	γ = 85.611(10)°
Volume	831.7(5) $Å^3$	
Z	2	
Density (calculated)	1.543 Mg/m^3	
Absorption coefficient	0.143 mm^{-1}	
F(000)	392	
Crystal size	0.288 x 0.121 x 0.032 mm^3	
Theta range for data collection	2.575 to 27.834°.	
Index ranges	–6<=h<=6, –17<=k<=17, –18<=l<=18	
Reflections collected	19264	
Independent reflections	3863 [R(int) = 0.0848]	
Completeness to theta = 25.242°	99.3 %	
Absorption correction	Semi-empirical from equivalents	
Max. and min. transmission	0.7456 and 0.6338	
Refinement method	Full-matrix least-squares on F^2	
Data / restraints / parameters	3863 / 611 / 379	
Goodness-of-fit on F^2	1.205	
Final R indices [I>2sigma(I)]	R1 = 0.1035, wR2 = 0.1856	
R indices (all data)	R1 = 0.1769, wR2 = 0.2084	
Largest diff. peak and hole	0.169 and –0.174 e·$Å^{-3}$	

Atomic coordinates (x 10^4) and equivalent isotropic displacement parameters ($Å^2$ x 10^3) for **4i**. U(eq) is defined as one third of the trace of the orthogonalized U^{ij} tensor.

	x	y	z	U(eq)
C(1)	281(6)	3836(2)	9609(2)	56(1)
C(2)	-252(6)	2887(2)	9327(2)	52(1)
C(3)	-2200(7)	2149(3)	9932(2)	70(1)
C(4)	-2753(9)	1272(3)	9699(3)	93(1)
C(5)	-1410(9)	1117(3)	8862(3)	89(1)
C(6)	507(7)	1841(2)	8258(2)	68(1)
C(7)	1108(6)	2741(2)	8476(2)	50(1)
C(8)	3166(7)	3548(2)	7802(2)	63(1)
C(9)	3848(6)	4429(2)	8145(2)	49(1)
N(10)	2487(5)	4515(2)	8963(2)	52(1)
N(11)	5827(5)	5101(2)	7577(2)	51(1)
O(12)	5649(4)	6158(2)	8585(1)	67(1)
C(13)	6527(6)	5935(2)	7827(2)	51(1)
C(14)	8627(6)	6668(2)	7084(2)	52(1)
C(15)	9770(6)	6457(2)	6237(2)	55(1)
C(16)	11645(6)	7160(3)	5557(2)	61(1)
C(17)	12376(7)	8068(3)	5709(2)	69(1)
C(18)	11234(7)	8289(2)	6550(2)	62(1)
C(19)	9382(6)	7586(2)	7238(2)	57(1)
C(20)	12850(10)	6925(4)	4646(3)	81(1)
F(21)	11250(30)	6242(15)	4373(12)	76(4)
F(22)	13080(60)	7778(10)	3824(8)	110(5)
F(23)	15340(30)	6401(18)	4706(11)	90(4)
F(21B)	11020(30)	6880(30)	4105(10)	104(5)
F(22B)	14830(50)	7695(13)	4098(14)	98(5)
F(23B)	14580(60)	5960(15)	4945(14)	99(5)
F(21C)	11300(50)	7619(18)	3899(12)	93(5)
F(22C)	15560(30)	7190(20)	4428(13)	93(5)
F(23C)	12580(70)	5970(14)	4702(14)	113(6)
C(24)	11720(40)	9342(13)	6700(13)	84(6)
F(25)	14510(30)	9330(12)	6900(20)	102(5)

F(26)	11310(50)	10179(13)	5874(10)	89(5)
F(27)	10110(50)	9461(15)	7504(11)	86(5)
C(24B)	12090(30)	9302(10)	6648(10)	75(4)
F(25B)	14710(20)	9596(9)	6199(10)	84(3)
F(26B)	10200(30)	10086(8)	6296(15)	108(4)
F(27B)	12150(50)	9196(10)	7607(7)	103(4)
C(24C)	11940(30)	9275(11)	6783(12)	85(5)
F(25C)	13280(50)	10011(11)	5996(8)	112(4)
F(26C)	9700(20)	9825(10)	7037(17)	101(4)
F(27C)	13720(30)	9026(10)	7527(10)	84(3)

Bond lengths [Å] and angles [°] for **4i**.

C(1)-N(10)	1.450(3)
C(1)-C(2)	1.492(4)
C(1)-H(1A)	0.9700
C(1)-H(1B)	0.9700
C(2)-C(7)	1.388(4)
C(2)-C(3)	1.388(4)
C(3)-C(4)	1.364(5)
C(3)-H(3)	0.9300
C(4)-C(5)	1.372(5)
C(4)-H(4)	0.9300
C(5)-C(6)	1.369(4)
C(5)-H(5)	0.9300
C(6)-C(7)	1.387(4)
C(6)-H(6)	0.9300
C(7)-C(8)	1.503(4)
C(8)-C(9)	1.480(4)
C(8)-H(8A)	0.9700
C(8)-H(8B)	0.9700
C(9)-N(10)	1.316(3)
C(9)-N(11)	1.336(3)
N(10)-H(10)	0.86(3)
N(11)-C(13)	1.345(3)
O(12)-C(13)	1.248(3)
C(13)-C(14)	1.505(4)
C(14)-C(19)	1.386(4)
C(14)-C(15)	1.388(4)
C(15)-C(16)	1.384(4)
C(15)-H(15)	0.9300
C(16)-C(17)	1.367(4)
C(16)-C(20)	1.495(5)
C(17)-C(18)	1.387(4)
C(17)-H(17)	0.9300
C(18)-C(19)	1.381(4)
C(18)-C(24B)	1.486(13)

C(18)-C(24)	1.519(16)
C(18)-C(24C)	1.522(15)
C(19)-H(19)	0.9300
C(20)-F(21B)	1.233(14)
C(20)-F(23C)	1.25(2)
C(20)-F(23)	1.301(14)
C(20)-F(22C)	1.315(15)
C(20)-F(22)	1.329(12)
C(20)-F(21C)	1.383(18)
C(20)-F(21)	1.392(14)
C(20)-F(22B)	1.398(15)
C(20)-F(23B)	1.42(2)
C(24)-F(26)	1.340(12)
C(24)-F(25)	1.359(13)
C(24)-F(27)	1.362(12)
C(24B)-F(26B)	1.307(12)
C(24B)-F(25B)	1.342(12)
C(24B)-F(27B)	1.344(12)
C(24C)-F(26C)	1.320(14)
C(24C)-F(25C)	1.348(14)
C(24C)-F(27C)	1.361(14)

N(10)-C(1)-C(2)	113.6(2)
N(10)-C(1)-H(1A)	108.8
C(2)-C(1)-H(1A)	108.8
N(10)-C(1)-H(1B)	108.8
C(2)-C(1)-H(1B)	108.8
H(1A)-C(1)-H(1B)	107.7
C(7)-C(2)-C(3)	120.0(3)
C(7)-C(2)-C(1)	121.7(2)
C(3)-C(2)-C(1)	118.3(3)
C(4)-C(3)-C(2)	120.1(3)
C(4)-C(3)-H(3)	119.9
C(2)-C(3)-H(3)	119.9
C(3)-C(4)-C(5)	120.3(3)
C(3)-C(4)-H(4)	119.8

C(5)-C(4)-H(4)	119.8
C(6)-C(5)-C(4)	120.1(3)
C(6)-C(5)-H(5)	119.9
C(4)-C(5)-H(5)	119.9
C(5)-C(6)-C(7)	120.8(3)
C(5)-C(6)-H(6)	119.6
C(7)-C(6)-H(6)	119.6
C(6)-C(7)-C(2)	118.6(3)
C(6)-C(7)-C(8)	120.9(3)
C(2)-C(7)-C(8)	120.6(2)
C(9)-C(8)-C(7)	116.5(2)
C(9)-C(8)-H(8A)	108.2
C(7)-C(8)-H(8A)	108.2
C(9)-C(8)-H(8B)	108.2
C(7)-C(8)-H(8B)	108.2
H(8A)-C(8)-H(8B)	107.3
N(10)-C(9)-N(11)	124.3(2)
N(10)-C(9)-C(8)	119.4(2)
N(11)-C(9)-C(8)	116.3(2)
C(9)-N(10)-C(1)	127.8(2)
C(9)-N(10)-H(10)	113(2)
C(1)-N(10)-H(10)	119(2)
C(9)-N(11)-C(13)	119.9(2)
O(12)-C(13)-N(11)	127.9(2)
O(12)-C(13)-C(14)	118.0(2)
N(11)-C(13)-C(14)	114.1(2)
C(19)-C(14)-C(15)	119.4(3)
C(19)-C(14)-C(13)	119.2(3)
C(15)-C(14)-C(13)	121.4(3)
C(16)-C(15)-C(14)	120.0(3)
C(16)-C(15)-H(15)	120.0
C(14)-C(15)-H(15)	120.0
C(17)-C(16)-C(15)	120.5(3)
C(17)-C(16)-C(20)	120.1(3)
C(15)-C(16)-C(20)	119.4(3)
C(16)-C(17)-C(18)	119.9(3)

C(16)-C(17)-H(17)	120.0
C(18)-C(17)-H(17)	120.0
C(19)-C(18)-C(17)	120.0(3)
C(19)-C(18)-C(24B)	123.5(6)
C(17)-C(18)-C(24B)	116.5(6)
C(19)-C(18)-C(24)	117.8(7)
C(17)-C(18)-C(24)	122.0(7)
C(19)-C(18)-C(24C)	116.4(7)
C(17)-C(18)-C(24C)	123.6(7)
C(18)-C(19)-C(14)	120.2(3)
C(18)-C(19)-H(19)	119.9
C(14)-C(19)-H(19)	119.9
F(23C)-C(20)-F(22C)	110.9(12)
F(23)-C(20)-F(22)	106.6(8)
F(23C)-C(20)-F(21C)	110.1(13)
F(22C)-C(20)-F(21C)	107.1(9)
F(23)-C(20)-F(21)	99.5(9)
F(22)-C(20)-F(21)	102.6(8)
F(21B)-C(20)-F(22B)	109.0(10)
F(21B)-C(20)-F(23B)	111.5(14)
F(22B)-C(20)-F(23B)	103.2(9)
F(21B)-C(20)-C(16)	114.5(7)
F(23C)-C(20)-C(16)	113.5(7)
F(23)-C(20)-C(16)	115.8(7)
F(22C)-C(20)-C(16)	110.5(8)
F(22)-C(20)-C(16)	115.2(7)
F(21C)-C(20)-C(16)	104.5(8)
F(21)-C(20)-C(16)	115.2(5)
F(22B)-C(20)-C(16)	110.0(8)
F(23B)-C(20)-C(16)	108.0(8)
F(26)-C(24)-F(25)	107.6(14)
F(26)-C(24)-F(27)	111.9(14)
F(25)-C(24)-F(27)	105.2(14)
F(26)-C(24)-C(18)	110.9(14)
F(25)-C(24)-C(18)	108.1(12)
F(27)-C(24)-C(18)	112.7(13)

F(26B)-C(24B)-F(25B)	109.4(11)
F(26B)-C(24B)-F(27B)	105.6(12)
F(25B)-C(24B)-F(27B)	107.5(12)
F(26B)-C(24B)-C(18)	110.8(10)
F(25B)-C(24B)-C(18)	112.7(10)
F(27B)-C(24B)-C(18)	110.6(10)
F(26C)-C(24C)-F(25C)	103.6(12)
F(26C)-C(24C)-F(27C)	106.2(13)
F(25C)-C(24C)-F(27C)	106.3(13)
F(26C)-C(24C)-C(18)	115.6(12)
F(25C)-C(24C)-C(18)	112.1(12)
F(27C)-C(24C)-C(18)	112.3(11)

Symmetry transformations used to generate equivalent atoms:

Anisotropic displacement parameters (Å ^{2} x 10^3) for A22013O_002. The anisotropic displacement factor exponent takes the form: $-2\pi^2[h^2a^{*2}U^{11} + ... + 2 h k a^* b^* U^{12}]$

	U^{11}	U^{22}	U^{33}	U^{23}	U^{13}	U^{12}
C(1)	58(2)	65(2)	49(2)	-23(1)	8(1)	-17(1)
C(2)	48(2)	52(2)	54(2)	-16(1)	-5(1)	-4(1)
C(3)	72(2)	66(2)	70(2)	-23(2)	11(2)	-18(2)
C(4)	110(3)	67(2)	99(3)	-26(2)	23(2)	-40(2)
C(5)	104(3)	62(2)	109(3)	-41(2)	10(2)	-29(2)
C(6)	78(2)	58(2)	75(2)	-33(2)	-2(2)	-3(2)
C(7)	51(2)	51(2)	51(2)	-19(1)	-6(1)	-2(1)
C(8)	74(2)	68(2)	53(2)	-31(2)	7(2)	-18(2)
C(9)	50(2)	56(2)	43(1)	-20(1)	-2(1)	-2(1)
N(10)	53(2)	59(2)	51(1)	-27(1)	6(1)	-13(1)
N(11)	56(2)	53(1)	45(1)	-18(1)	5(1)	-8(1)
O(12)	79(2)	71(1)	59(1)	-35(1)	18(1)	-27(1)
C(13)	52(2)	54(2)	45(2)	-16(1)	1(1)	-5(1)
C(14)	48(2)	56(2)	48(2)	-13(1)	-4(1)	-3(1)
C(15)	54(2)	58(2)	50(2)	-16(1)	-3(1)	-1(1)
C(16)	51(2)	72(2)	48(2)	-8(2)	2(1)	-2(2)
C(17)	54(2)	71(2)	63(2)	1(2)	4(2)	-12(2)
C(18)	58(2)	52(2)	66(2)	-6(2)	-4(2)	-7(2)
C(19)	58(2)	57(2)	53(2)	-13(1)	-4(1)	-3(2)
C(20)	73(3)	103(3)	56(2)	-17(2)	10(2)	-8(3)
F(21)	67(6)	97(9)	76(8)	-48(7)	0(5)	-1(6)
F(22)	137(13)	127(7)	49(4)	-13(4)	11(8)	-2(10)
F(23)	82(7)	114(12)	68(7)	-31(8)	2(5)	18(8)
F(21B)	99(7)	154(14)	58(7)	-36(10)	-15(5)	10(12)
F(22B)	88(10)	97(8)	75(9)	5(6)	34(7)	4(7)
F(23B)	110(12)	97(9)	84(8)	-33(6)	15(7)	18(8)
F(21C)	108(10)	104(10)	55(6)	-12(7)	-17(7)	15(10)
F(22C)	57(6)	125(14)	72(9)	-11(9)	24(6)	3(9)
F(23C)	125(13)	113(9)	84(10)	-27(7)	20(10)	37(11)
C(24)	83(8)	66(8)	93(8)	-14(8)	-3(8)	-14(8)
F(25)	88(8)	78(8)	140(12)	-29(9)	-31(10)	-21(6)

F(26)	91(11)	58(6)	98(8)	-6(6)	5(8)	6(8)
F(27)	112(10)	62(9)	81(8)	-23(6)	-3(8)	-5(8)
C(24B)	78(7)	58(6)	83(6)	-15(6)	-3(6)	-18(6)
F(25B)	67(5)	72(6)	99(7)	-10(5)	-3(5)	-20(4)
F(26B)	115(8)	54(5)	144(10)	-20(7)	-12(7)	10(5)
F(27B)	139(11)	82(6)	97(5)	-39(4)	0(8)	-26(8)
C(24C)	81(7)	63(7)	96(8)	-9(7)	0(7)	-14(7)
F(25C)	123(11)	70(7)	122(7)	-5(5)	11(8)	-41(7)
F(26C)	106(6)	58(7)	139(11)	-37(7)	-1(8)	-10(5)
F(27C)	84(7)	87(6)	86(6)	-36(5)	5(6)	-12(5)

Hydrogen coordinates (x 10^4) and isotropic displacement parameters ($Å^2$ x 10^3) for **4i**.

	x	y	z	U(eq)
H(1A)	-1505	4264	9599	68
H(1B)	850	3584	10281	68
H(3)	-3129	2253	10497	84
H(4)	-4048	776	10109	111
H(5)	-1802	519	8705	107
H(6)	1418	1727	7695	82
H(8A)	4957	3165	7695	75
H(8B)	2367	3867	7166	75
H(10)	3050(70)	5040(30)	9110(20)	78
H(15)	9275	5843	6127	66
H(17)	13638	8537	5248	83
H(19)	8639	7731	7806	69

Hydrogen bonds for **4i** [Å and °].

D-H...A	d(D-H)	d(H...A)	d(D...A)	<(DHA)
N(10)-H(10)...O(12)	0.86(3)	1.89(3)	2.593(3)	138(3)

Symmetry transformations used to generate equivalent atoms:
